# Photocatalytic Synthesis of Unprotected Sulfonimidamides and Their Application in Photochemical Nitrene Transfer Reactions

**DOI:** 10.1002/anie.202509870

**Published:** 2025-08-21

**Authors:** Ivan Sliusarevskyi, Jordan Diaz, Sudip Senapati, Ben J. Ebel, Nico J. Linnartz, Iris M. Oppel, Claire Empel, Philip Wai Hong Chan, Rene M. Koenigs

**Affiliations:** ^1^ RWTH Aachen University Institute of Organic Chemistry Landoltweg 1 52074 Aachen Germany; ^2^ Monash University School of Chemistry Clayton Victoria 3800 Australia; ^3^ University of Bayreuth Organic Chemistry II Universitätsstr. 30 95447 Bayreuth Germany; ^4^ RWTH Aachen University Institute of Inorganic Chemistry Landoltweg 1 52074 Aachen Germany

**Keywords:** Amination, Cycloaddition, Nitren, Photocatalysis, Sulfur

## Abstract

The selective incorporation of nitrogen into organic molecules remains a central challenge in modern synthetic chemistry, particularly when aiming to access complex, functionalized scaffolds under mild and sustainable conditions. In this report, we describe a photocatalytic method that employs hydroxylamine‐derived nitrene precursors and unprotected sulfinamides under mild conditions, enabling efficient nitrene transfer without the need for stoichiometric amounts of an oxidant. The resulting unprotected sulfonimidamides serve not only as target compounds but also as versatile intermediates for further nitrene‐transfer reactions, affording complex sulfur–nitrogen‐rich frameworks. Mechanistic studies support a photoinduced single‐electron transfer process involving a reactant complex between the photocatalyst and nitrene precursor. This work establishes a general and modular platform for accessing challenging architectures based on sulfonimidamides, expanding the toolkit for late‐stage functionalization and heteroatom‐rich molecule construction.

## Graphical abstract

A visible‐light‐driven, metal‐free nitrene transfer enables the efficient synthesis of unprotected sulfonimidamides from sulfinamides and hydroxylamine‐derived reagents, offering broad substrate scope, late‐stage functionalization, and access to complex sulfur–nitrogen scaffolds.

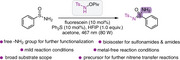

The ability to efficiently and selectively incorporate nitrogen into organic molecules is a fundamental goal in modern synthetic chemistry, with far‐reaching implications across various scientific disciplines.^[^ ^1^, ^2^, ^3^, ^4^, ^5^, ^6^, ^7^, ^8^, ^9^, ^10^, ^11^, ^12^, ^13^ ^]^ Developing new methods for constructing carbon–nitrogen bonds thus remains central to this pursuit. Among these, nitrene transfer reactions^[^ ^8^, ^9^, ^10^, ^11^, ^12^, ^13^ ^]^ have proven to be a powerful strategy (Scheme 1a); however, their reliance on harsh conditions, metal‐based reagents, and stoichiometric amounts of an oxidant has limited their practicality and sustainability. Recently, the use of visible light to drive nitrene transfer reactions has emerged as a promising alternative,^[^ ^14^, ^15^, ^16^, ^17^, ^18^, ^19^, ^20^, ^21^, ^22^, ^23^ ^]^ offering milder and more sustainable pathways for complex molecular transformations. These approaches typically involve reactive nitrene radical anions^[^ ^22^ ^]^ or free triplet nitrene^[^ ^14^, ^22^ ^]^ as key intermediates, enabling access to previously challenging transformations, including advanced cycloaddition reactions that extend beyond, for example, conventional aziridination chemistry (Scheme 1b).^[^ ^21^ ^]^

**Scheme 1:**
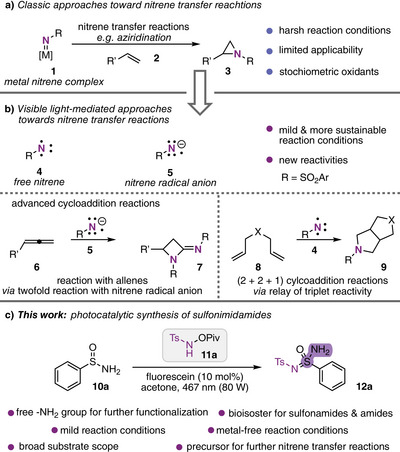
a) Classic nitrene transfer reactions. b) Light‐induced nitrene transfer reactions. c) Light‐induced synthesis of unprotected sulfonimidamides.

Building on our interest in photoinduced nitrene transfer reactions,^[^ ^21^, ^22^, ^23^ ^]^ we turned our attention to the synthesis of sulfonimidamides,^[^ ^24^, ^25^, ^26^, ^27^, ^28^, ^29^, ^30^, ^31^ ^]^ which are characterized by a sulfur–nitrogen double bond and an additional nitrogen substituent. Sulfonimidamides are widely recognized as bioisosteres of sulfonamides and amides, making them valuable in pharmaceutical and agrochemical research.^[^ ^24^, ^25^ ^]^ Among the available methods to construct these structures, the nitrene transfer to sulfinamides offers a direct and efficient route.^[^ ^26^, ^27^, ^28^, ^29^, ^30^, ^31^ ^]^ However, the synthesis of unprotected sulfonimidamides bearing a free ─NH_2_ group remains a significant challenge and requires multistep synthesis routes.^[^ ^13^, ^32^, ^33^ ^]^ Unprotected sulfonimidamides are particularly desirable as reagents as they can participate in consecutive nitrene transfer reactions or can be used as sulfonamide bioisosters and facilitate the further functionalization of molecular scaffolds.

To address this challenge, we considered the use of unprotected sulfinamides in the reaction with the hydroxylamine‐derived reagent **11a** (Scheme 1c). To our delight, the desired product could be obtained in moderate yield of 60% in the presence of the organic dye fluorescein as the photocatalyst (Table 1, entry 1). A strong dependency on the reaction solvent was made and acetone was identified as the optimum solvent for this transformation (Table 1, entry 2). In these experiments, we identified tosyl amide as the most prominent by‐product, which we assumed to be related to undesired decomposition of intermediate nitrene species. We therefore considered the use of diphenyl sulfide as an additive that can form an intermittent sulfilimine that was recently shown as an additive to improve nitrene transfer reactions by Song and co‐workers.^[^ ^17^ ^]^

**Table 1:** Optimization of the reaction conditions.

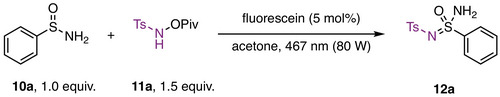		
Entry	Changes from above	Yield 12a / %
1	None	60
2	MeCN / DCM	22 / 21
3	+ Ph_2_S (10 mol%)	70
4	+ HFIP (1 equiv.)	87
5	+ Ph_2_S (10 mol%) +HFIP (1 equiv.)	95
6	No catalyst	*No reaction*
7	No light	*No reaction*
8	PhCO_2_NHOPiv instead of **11a**	*Dec. of nitrene precursor*
9	PivNHOPiv instead of **11a**	*Dec. of nitrene precursor*
10	PhINTs intead of **11a**	*Dec. of nitrene precursor*
11	+ TEMPO	*Dec. of nitrene precursor*

*Reaction conditions*: A mixture of **10a** (0.2 mmol, 1.0 equiv.), **11a** (1.5 equiv.) and fluorescein (5 mol%) in acetone (2 mL) was irradiated under argon atmosphere with 467 nm (80 W) light. Dec. = decomposition.

Furthermore, we considered the addition of slightly acidic HFIP as a proton source to the reaction to trap the basic species as it forms during the reaction and to help regenerate the fluorescein catalyst by protonation. Indeed, both additives individually gave a significant improvement in the product yield (Table 1, entries 3,4). When combining both additives, the reaction yield could be significantly improved to furnish the desired sulfonimidamide in excellent isolated yield of 95% (Table 1, entry 5). Here, it should be noted that no reaction occurred in the absence of light or the photocatalyst (Table 1, entries 6,7). We next examined the possibility of amide‐protected nitrenes and a sulfonyl‐protected iodinane as nitrene precursors (Table 1, entries 8–10) in this transformation, yet in all cases only the decomposition of the nitrene precursor was observed. In the presence of TEMPO as a radical trap, no reaction occurred (Table 1, entry 11). A sensitivity screen showed that deviations from the optimized reaction conditions still gave satisfactory yields of the reaction product. The most critical parameters were found to be the addition of water, longer wavelength, or performing the reaction in air (see Table S1 in the Supporting Information).

With the optimized conditions in hand, we proceeded to investigate the substrate scope of the transformation (Scheme 2). We began by exploring the influence of different sulfonyl protecting groups and were pleased to find that a broad range of sulfonyl‐protected nitrene precursors could be efficiently transferred to the sulfinamide under photocatalytic conditions (**12a**–**12m**) (Scheme 2a). Aromatic, heterocyclic, vinylic and aliphatic substituted sulfonyl nitrenes proved compatible with the present reaction conditions. In all cases, a good yield of the respective unprotected sulfonimidamide was obtained.

**Scheme 2:**
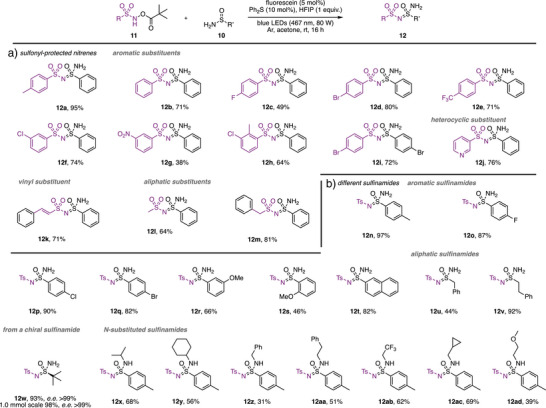
Investigations on the substrate scope of unprotected sulfonimidamides. a) evaluation of sulfonyl‐protected nitrenes. b) evaluation of sulfinamides.

Subsequent studies focused on the compatibility of various sulfinamide substrates. Aryl sulfinamides bearing substituents at all positions of the aromatic ring were generally well tolerated, affording the corresponding sulfonimidamides (**12n**–**12t**) in good yields. Notably, a slight decrease in yield was observed for the ortho‐substituted aryl sulfinamide derivative, likely due to steric hindrance. We further examined the reaction's applicability to aliphatic sulfinamides and *N*‐protected aryl sulfinamides. In both cases, the desired sulfonimidamides (Scheme 2b, **12u**–**12ad**) were obtained in excellent yields. Particularly, noteworthy was the successful conversion of a chiral Ellman auxiliary to the corresponding sulfonimidamide (**12w**) with high levels of stereoselectivity. Among the *N*‐protected aryl sulfinamides, we could demonstrate the compatibility with primary and secondary alkyl groups. In this case, different groups such as ethers (**12ad**), cyclopropanes (**12ac**), or trifluoromethyl groups (**12ab**). Despite the broad substrate tolerance, the methodology exhibits certain limitations. Specifically, aryl‐protected sulfinamides or *N,N*‐dialkylated sulfinamides proved unreactive under the optimized photocatalytic conditions, with no observable nitrene transfer (for details, please see ESI Figure S15 in the Supporting Information).

We next sought to evaluate the broader applicability of this methodology. As an initial step, we investigated its compatibility with more complex drug molecules. Derivatives of valdecoxib were employed both as the nitrene precursor (**12ae**) and as the sulfinamide reagent (**12af**). In both cases, the transformation proceeded smoothly to furnish the corresponding sulfonimidamides in good yields. Notably, the two products differ only in the positioning of the unprotected amine functionality and the pharmacophore, offering distinct vectors for introduction of further substituents. In a similar fashion, celecoxib could be applied as precursor for nitrene transfer to sulfilimine to give **12ag** (Scheme 3a).

**Scheme 3:**
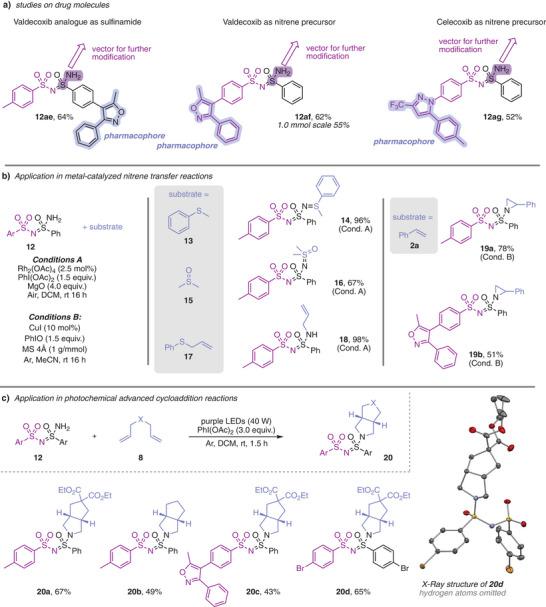
Applications of unprotected sulfonimidamides. a) Applications of drug molecules as nitrene precursors or sulfinamides. b) Applications of unprotected sulfonimidamides in metal‐catalyzed nitrene transfer reactions under oxidative conditions. c) Applications in photochemical cycloaddition reactions.

Building on our previous work,^[^ ^14^, ^21^, ^22^, ^23^ ^]^ we further investigated the synthetic utility of the unprotected amine‐bearing sulfonimidamides in downstream nitrene transfer reactions. In the first series of experiments, we explored the direct transfer of the entire sulfonimidamide unit onto various organosulfur substrates under oxidative conditions for in situ synthesis of the nitrene precursor and Rh_2_(OAc)_4_ as the nitrene transfer catalyst (Scheme 3b). Depending on the nature of the organosulfur compound used, this approach enables the efficient synthesis of diverse hybrid structures in high yields, e.g., mixed sulfilimine/sulfonimidamides, sulfoximine/sulfonimidamides, and sulfinamide/sulfonimidamides. These scaffolds, composed exclusively of sulfur and nitrogen atoms in the backbone, remain synthetically challenging yet highly desirable building blocks in modern sulfur–nitrogen chemistry. Further studies comprised the evaluation of the synthesis of sulfonimidamide‐substituted aziridines. In this case, iodosobenzene was used as an oxidant and copper iodide as catalyst to furnish the desired aziridines in good yield (Scheme 3b).

Finally, we explored these unprotected sulfonimidamides in a photochemical cycloaddition reaction with nonconjugated 1,6‐dienes. We got particularly intrigued by the question, which type of cycloaddition occurs when using the sulfonimidamide‐derived nitrene precursor as an aziridination or (2 + 1) cycloaddition, an Aza‐Pauson‐Khand or (2 + 2 + 1) cycloaddition, or an even more complex (2 + 2 + 3) cycloaddition may occur. In the latter case, both nitrogen atoms of the sulfonimidamide would engage in such cycloaddition reaction. To our delight, we could obtain a single reaction product in moderate to good yields using different 1,6‐dienes and nitrene precursors. Single crystal analysis was employed to confirm the structural assignment and provides unambigious proof of an exclusive (2 + 2 + 1) cycloaddition reaction (Scheme 3c).

We concluded our investigations with experimental studies on the reaction mechanism. The on/off experiment shows that no reaction occurs in the absence of light. A quantum yield of *Φ* = 0.14 further confirms the absence of radical chain mechanism (Scheme 4a). UV/Vis studies show that the nitrene transfer reagent **11a** does not absorb light in the (near‐)visible region. These studies further show a marked change of the absorption properties of fluorescein upon addition of 1 or 10 equiv. of the nitrene transfer reagent **11a** (Scheme 4b, for the change of visual appearance, please see ESI Figure S8). These studies suggest the formation of a weak reactant complex of **11a** with the fluorescein catalyst. This is further underlined in fluorescence quenching studies, where the temperature dependency indicates static quenching (Scheme 4c). Cyclic voltammetry further supports an irreversible electron transfer (*E*_1/2_ = –0.98 V, Scheme 4d).

**Scheme 4:**
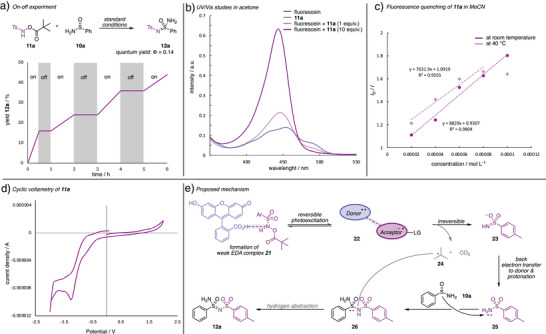
Experimental studies on the reaction mechanism and proposed reaction mechanism. a) On/off experiment. b) UV/Vis studies on the formation of a reactant complex. c) Temperature‐dependent fluorescence quenching for evaluation of static quenching. d) Cyclic voltammetry studies. e) Proposed reaction mechanism.

These observations led to the conclusion that the reaction mechanism proceeds through initial formation of a reactant complex (Scheme 4e).^[^ ^34^ ^]^ Single electron transfer leads to formation of the reactive nitrogen‐based intermediate **25** that undergoes addition reaction with sulfinamide **10a** and formation of the sulfonimidamide reaction product **12a**. The intermittency of radicals is further substantiated by a control reaction in the presence of TEMPO, where complete inhibition of the product formation was observed (see Table 1, entry 11). HRMS analysis of this reaction mixture shows the formation of TEMPO adducts (for details, please see ESI Figure S14).

In summary, we report a visible‐light‐enabled method for the synthesis of unprotected sulfonimidamides via direct nitrene transfer from hydroxylamine‐derived precursors to sulfinamides. This photocatalytic transformation proceeds under mild, metal‐free conditions and tolerates a broad range of substrates, including aryl and aliphatic sulfinamides, as well as complex drug molecules. The resulting sulfonimidamides serve as versatile intermediates for further functionalization, enabling access to challenging sulfur–nitrogen architectures as well as cycloaddition chemistry. Mechanistic studies support a photoinduced single‐electron transfer process involving a reactant complex between the photocatalyst and the nitrene precursor. This strategy offers a broadly applicable platform for sulfur–nitrogen bond construction and downstream diversification.

## Supporting Information

The authors have cited additional references within the Supporting Information.

## Conflict of Interests

The authors declare no conflict of interest.

## Supporting information



Supporting Information

## Data Availability

The data that support the findings of this study are available in the Supporting Information of this article.
