# Supplementary material for: Photocatalytic Synthesis of Unprotected Sulfonimidamides and Their Application in Photochemical Nitrene Transfer Reactions
Source: Angew Chem Int Ed Engl. 2025 Aug 21;64(39):e202509870. doi: 10.1002/anie.202509870 (PMC12455421; doi:10.1002/anie.202509870)

# Supporting Information

# Photocatalytic synthesis of unprotected sulfonimidamides and their application in photochemical nitrene transfer reactions

Ivan Sliusarevskyi,[a] Jordan Diaz,[b] Sudip Senapati,[c] Ben J. Ebel,[d] Nico J. Linnartz,[d] Iris M. Oppel,[d] Claire Empel,[a,c] Philip Wai Hong Chan*[b] and Rene M. Koenigs*[a,c]

[a] RWTH Aachen University, Institute of Organic Chemistry, Landoltweg 1, 52074 Aachen, Germany
[b] Monash University, School of Chemistry, Clayton, Victoria 3800, Australia
[c] University of Bayreuth, Organic Chemistry II, Universitätsstr. 30, 95447 Bayreuth, Germany
[d] RWTH Aachen University, Institute of Inorganic Chemistry, Landoltweg 1, 52074 Aachen, Germany

E-mail: phil.chan@monash.edu
E-mail: rene.koenigs@uni-bayreuth.de

## Table of Contents

1. General Information S1
2. Reaction Optimization S2
3. General Procedures S3
4. Stern-Volmer Fluorescence Quenching Studies S7
5. UV-Vis Experiment S11
6. Quantum Yield Determination S12
7. Cyclic Voltammetry Measurements S15
8. Radical Trapping Experiment S17
9. Incompatible Substrates S18
10. Crystallographic Studies S19
11 HPLC Data S21
12. Physical Data S23
13. References S43
14. NMR Spectra S44

# 1. General Information

Unless otherwise noted, all commercially available compounds were used as provided without further purification. Chemicals used in this manuscript were purchased from Sigma Aldrich, Alfa Aesar, Fluorochem, BLD Farm and Carl Roth. Solvents used in reactions were p.A. grade. Solvents for chromatography were technical grade and distilled prior to use. Analytical thin-layer chromatography (TLC) was performed on Macherey-Nagel silica gel aluminium plates with F-254 indicator, visualized by irradiation with UV light. Column chromatography was performed using silica gel Merck 60 (particle size 0.063 – 0.2 mm). Solvent mixtures are understood as volume/volume. $^{1}$H NMR, $^{13}$C NMR and $^{19}$F NMR were recorded on a Varian AV600/AV400 or an Agilent DD2 400 NMR spectrometer in $CDCl_3$ and DMSO-$d_6$. Data are reported in the following order: chemical shift (δ) in ppm; multiplicities are indicated br (broadened singlet), s (singlet), d (doublet), t (triplet), q (quartet), m (multiplet); coupling constants (*J*) are in Hertz (Hz). HRMS data were recorded on a ThermoFisher Scientific LTQ Orbitrap XL using ESI ionization or on a Finnigan MAT 95 using EI ionization at 70 eV. LEDs used in this manuscript were purchased from Kessil: KSPR160L PR160L Rating: 19VDC, 40W Max. 1$^{st}$ event

## 2. Reaction Optimization

**Table S1:** Reaction Optimization.

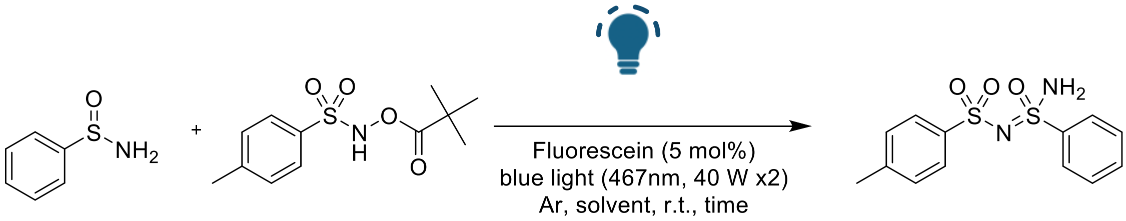


10a 11a 12a

| Entry | 10a:11a | Solvent | Additive | Time | Yield |
|---|---|---|---|---|---|
| 1 | 1:1.5 | MeCN | - | 16 h | 22%[a] |
| 2[b] | 1:1.5 | DCM | - | 16 h | 21%[a] |
| 3[c] | 1:1.5 | DCM | - | 16 h | 25%[a] |
| 4[d] | 1:1.5 | DCM | - | 16 h | 11% (23%[a]) |
| 5[e] | 1:1.5 | DCM | - | 16 h | 18%[a] |
| 6 | 1:1.5 | Acetone | - | 16 h | 44% (57%[a]) |
| 7 | 1:2 | Acetone | - | 48 h | 60%[a] |
| 8 | 1:2 | Acetone | - | 72 h | 48% (61%[a]) |
| 9[f] | 1:2 | Acetone | - | 24 h | 60%[a] |
| 10 | 1:2 | Acetone | MS (3Å) | 16 h | No reaction |
| 11 | 1:2 | Acetone | HFIP (1 equiv.) | 16 h | 87% |
| 12 | 1:2 | Acetone | HFIP (2 equiv.) | 16 h | 77% |
| 13 | 1:2 | Acetone | HFIP (3 equiv.) | 16 h | 68% (76%[a]) |
| 14 | 1:2 | Acetone | $Ph_2S$ (10 mol%) | 16 h | 70% (76%[a]) |
| 15 | 1:2 | Acetone | Ph2S (1 equiv.) | 16 h | 83% |
| 16 | 1:2 | Acetone | $Ph_2S$ (10 mol%), HFIP (2 equiv.) | 16 h | 90% |
| 17 | 1:2 | Acetone | $Ph_2S$ (1 equiv.) HFIP(1 equiv.) | 16 h | 74% (90%[a]) |
| 18 | 1:2 | Acetone | $Ph_2S$ (1 equiv.), HFIP (3 equiv.) | 16 h | 67% (82%[a]) |
| 19 | 1:2 | Acetone | $Ph_2S$ (10 mol%), HFIP (3 equiv.) | 16 h | 87% |
| 20 | 1:2 | Acetone | $Ph_2S$ (10 mol%), HFIP (1 equiv.) | 16 h | **95% (99%[a])** |

*Reaction conditions:* **10a**, **11a**, Fluorescein (5 mol%), solvent (0.1 M wrt 1), rt. [a]Calculated yields were determined by 1H NMR integration against an internal standard (1,3,5-trimethoxybenzene), isolated yield in parentheses. [b]7.5mol% of Fluorescein was used. [c]10 mol% of Fluorescein was used. [d]20 mol% of Fluorescein was used. [e]5 mol% of 4-CzIPN was used. [f]After 6 h second portion of Fluorescein (5 mol%) was added

## 3. General Procedures

**General procedure for the preparation of *N*-unsubstituted sulfinamides (10) *via* methylsulfinates (GP1)[1]**

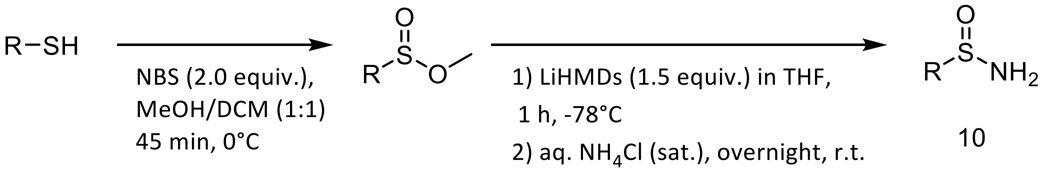


10

**Step 1:** *N*-bromo succinimide (10 mmol, 2.0 equiv.) was added to a solution of a thiol (5 mmol, 1.0 equiv.) in DCM/MeOH (1/1, V/V, 50 mL, 0.1 M) at 0 °C and stirred for 5 min. Subsequently, the reaction was warmed to room temperature and stirred for 45 min. Then, the mixture was quenched with saturated $NaHCO_3$ solution, washed with hot water, and the aqueous phase was extracted with DCM (3 × 15 mL). The combined organic layers were washed with brine, dried over $MgSO_4$ and concentrated under reduced pressure. The resulting methylsulfinate was used in the next step without further purification.

**Step 2:** A solution of a methylsulfinate in anhydrous THF (25 mL) was cooled to –78°C. LiHMDs (1.3 M in THF, 1.5 equiv.) was added dropwise while syringe under argon. The reaction mixture was subsequently stirred for 1 h at –78°C. Then, the reaction was warmed to room temperature and stirred for 30 min. An aqueous saturated $NH_4Cl$ solution (10 mL) was added slowly, and the mixture was stirred overnight. Subsequently, the reaction was diluted with EtOAc, and the aqueous phase was extracted with EtOAc (3 × 15 mL). The combined organic layers were washed with brine, dried over $MgSO_4$ and concentrated under reduced pressure. The crude product was purified by column chromatography or titration in $Et_2O$ to afford the pure sulfinamide.

**General procedure for the preparation of *N*-unsubstituted sulfinamides via methylsulfinates (10) (GP2)[2]**

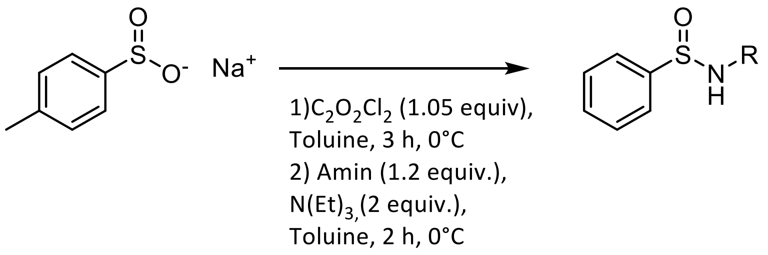


To a round bottomed flask was added sodium arylsulfinate (3.0 mmol, 1 equiv.) in anhydrous toluene (7 mL). After cooling the solution to 0 °C oxalyl chloride (3.15 mmol, 1.05 equiv.) was added dropwise. The reaction mixture was heated to room temperature during 3 h to generate sulfinyl chloride in situ. A second round bottomed flask was charged with the corresponding amine (3.6 mmol, 1.2 equiv.) and trimethylamine (6.0 mmol, 2 equiv.) in anhydrous toluene (20 mL). To this flask, the in situ generated sulfinyl chloride was added dropwise at 0 °C. The reaction mixture was stirred for 2 h at room temperature and then poured in $H_2O$ (40 mL) and extracted with EtOAc (20 mL) three times. The combined organic layers were dried over anhydrous $Na_2SO_4$ and concentrated in vacuo. The product was purified by silica column chromatography.

**General procedure for the preparation of *N*-(pivaloyloxy)sulfonamides (11) (GP3)[3]**

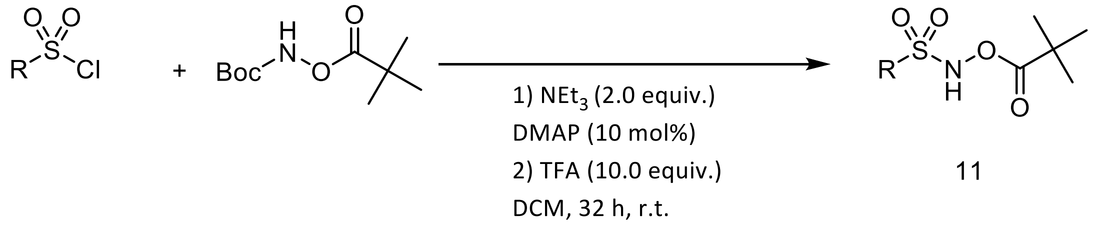


To a solution of BocNHOPiv (2.3 mmol, 1.0 equiv.) and DMAP (0.23 mmol, 10 mol%) in DCM (50 mL) was added $NEt_3$ (4.6 mmol, 2.0 equiv.) followed by the addition of sulfonyl chloride (2.5 mmol, 1.1 equiv.). The solution was left to stir at room temperature for 16 hours. To the solution TFA (23.0 mmol, 10.0 equiv.) was slowly added and the solution was continued to stir for 16 h. The reaction was then diluted with DCM and washed with once with 1 M HCl. The aqueous layer was extracted a further two times with DCM before the combined organic phases were washed with brine, dried over $MgSO_4$ and concentrated under reduced pressure.

## General procedure for the synthesis of unprotected sulfonimidamides (12) (GP4)

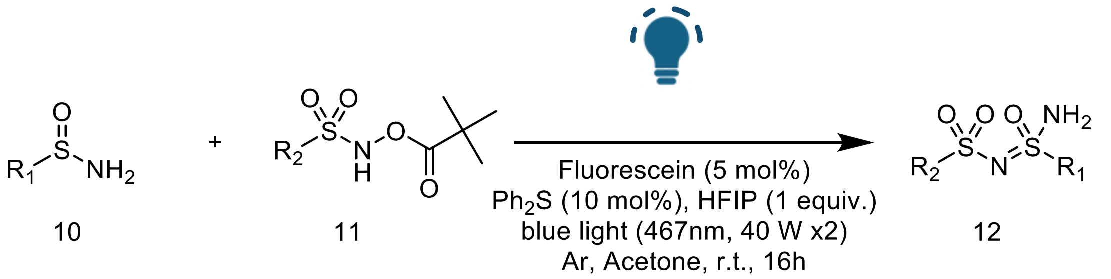


Test tube with **10** (0.2 mmol, 1.0 equiv. **11** (0.4 mmol, 2.0 equiv.) and Fluorescein (5 mol%), was degassed three times with Ar. After it HFIP (0.2 mmol, 1.0 equiv.) and Diphenyl sulfide (0.02 mmol, 0.1 equiv.) dissolved in 2 mL dry acetone was added to the test tube via syringe. The resulting reaction mixture was irradiated with blue light (2 x 40 W 467 nm) for 16 h. After finishing the reaction, the solvent was removed under reduced pressure and the product was purified by silica column chromatography DCM : MeOH : $NH_3$ as eluent.

## General procedure for (GP5)[4]

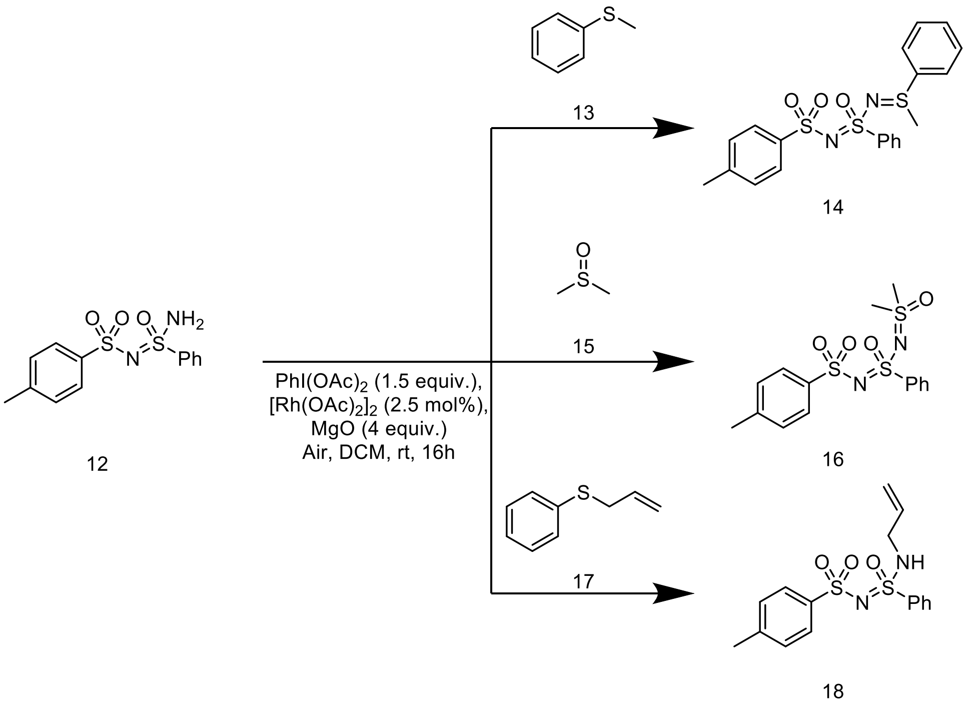


To the test tube with sulfur-containing compound (0.1 mmol), **12** (0.2 mmol 2 equiv.), (diacetoxyiodo)benzene (0.15 mmol 1.5 equiv.), magnesium oxide (0.4 mmol 4 equiv.) and $[Rh(OAc)_2]_2$ (2.5 mol%) was added 1 ml of dry DCM. The resulting reaction mixture was stirred for 16 h at room temperature. After finishing the reaction, the solvent was removed under reduced pressure and the product was purified by silica column chromatography using DCM : MeOH as eluent.

## General procedure for the synthesis of aziridines (19) (GP6)[5]

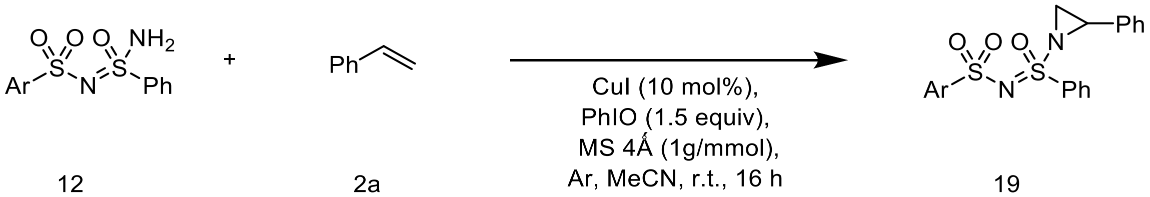


12 2a 19

Test tube with **12** (0.2 mmol 2 equiv), Iodosobenzene (0.15 mmol 1.5 equiv.), molecular sieves 4 Å (100mg) and CuI (10 mol%), was degassed three times with Ar. After it **2** (0.1 mmol, 1 equiv.) dissolved in 1 mL dry MeCN was added to the test tube via syringe. The resulting reaction mixture was steer for 16 h. After finishing the reaction, the solvent was removed under reduced pressure and the product was purified by silica column chromatography DCM : MeOH as eluent.

## General procedure for (2+2+1)-cycloaddition reactions (20) (GP7)[6]

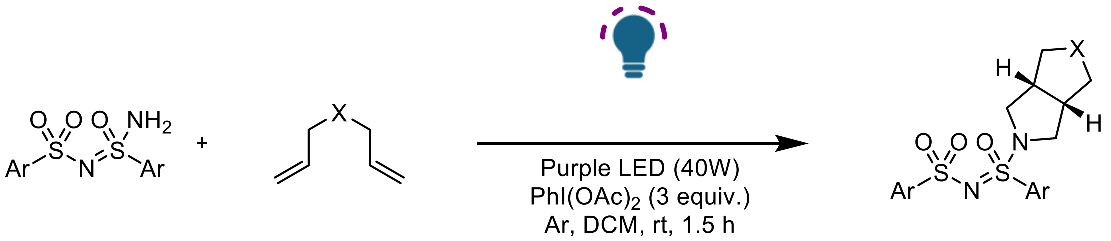


12 8 20

Test tube with **12** (0.3 mmol 3 equiv.) and (Diacetoxyiodo)benzene (0.3 mmol 3 equiv.) was degassed three times with Ar. After **8** (0.1 mmol, 1.0 equiv.) dissolved in 1 mL dry DCM was added to the test tube via syringe. The resulting reaction mixture was irradiated with Purple LED (40W) for 1.5 h. After finishing the reaction, the solvent was removed under reduced pressure and the product was purified by silica column chromatography *n*-Pentane : $Et_2O$ as eluent.

## 4. Stern-Volmer Fluorescence Quenching Studies

Fluorescence quenching experiments were performed on a Simazu RF-6000 Spectro Fluorophotometer. Fluorescein solutions were excited at 435 nm and emission intensity at 581 nm were collected. All the measurements were carried out mixing a solution of 2 ×10-7 M solution of Fluorescein in dry degassed acetonitrile and appropriate amount of quencher dissolved in 2 ×10-7 M solution of Fluorescein in a screw top 1.0 cm quartz cuvette. Samples were degassed three times then the emission spectra of the samples were collected. For a higher temperature measurement, each sample was heated in a water bath for 5 minutes. $I_0$ is the intensity without quencher, and I is the intensity with quencher. Plots were drawn according to the Stern-Volmer equation and $K_q$ were calculated.

Stern-Volmer equation: $I_0/I = 1+K_q[Q]$

**Emission quenching Studies with 10a and 11a**

Increasing amount of quencher were added to a solution of Fluorescein in MeCN.
After each addition emission spectra were recorded.

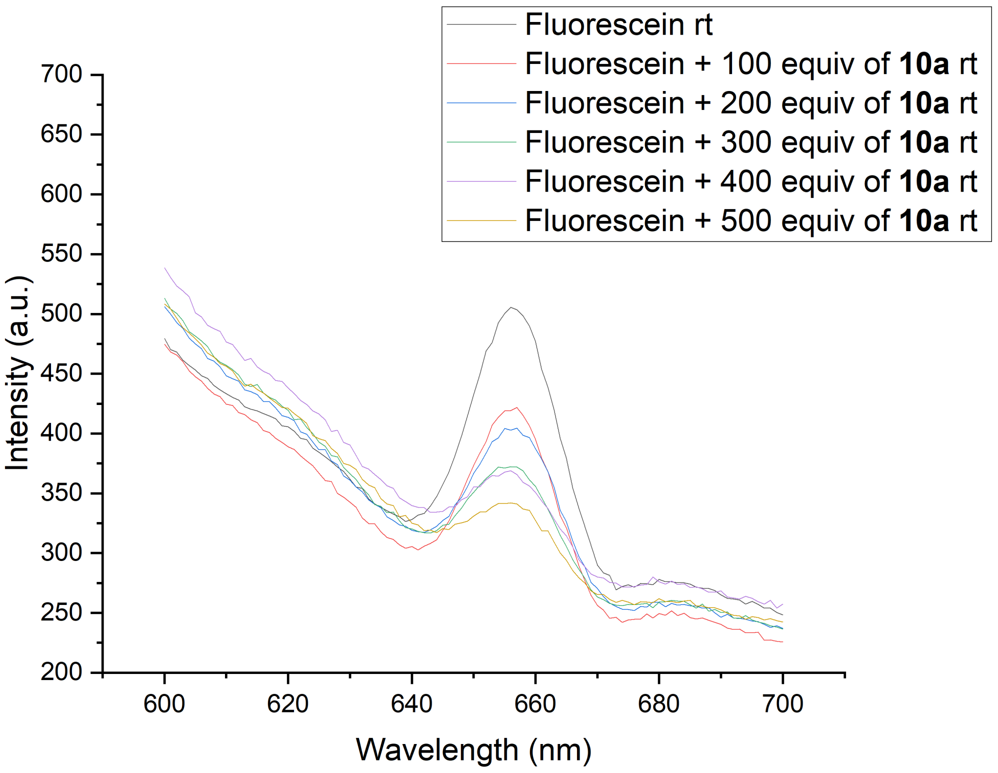


**Figure S1:** Fluorescence quenching with **10a** at rt.

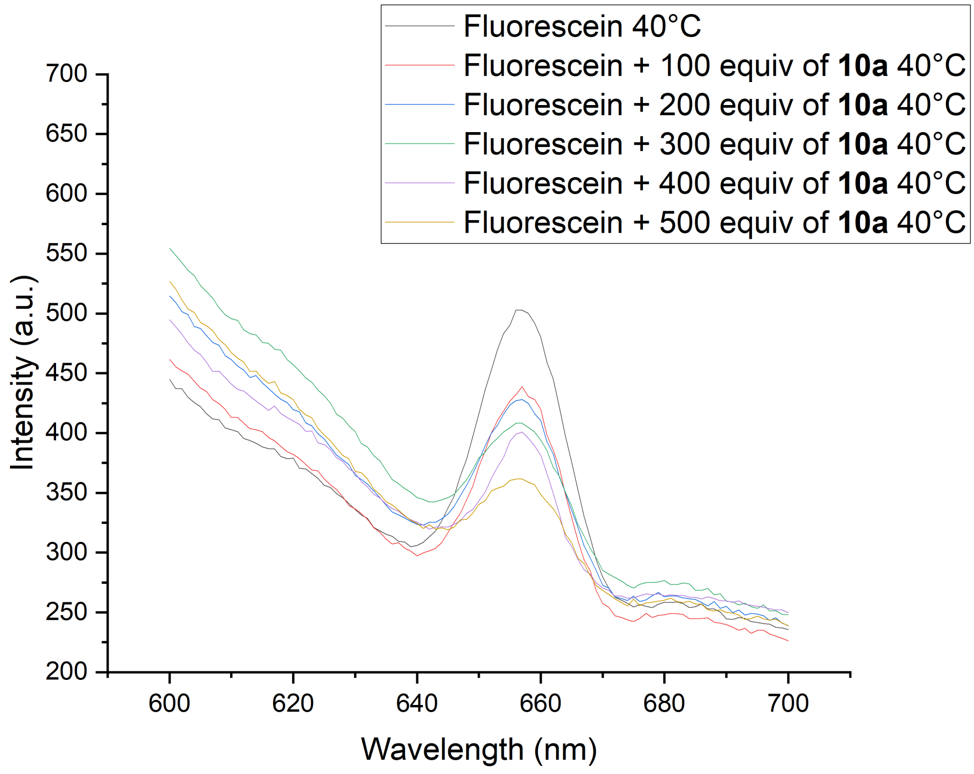


**Figure S2:** Fluorescence quenching with **10a** at 40 °C.

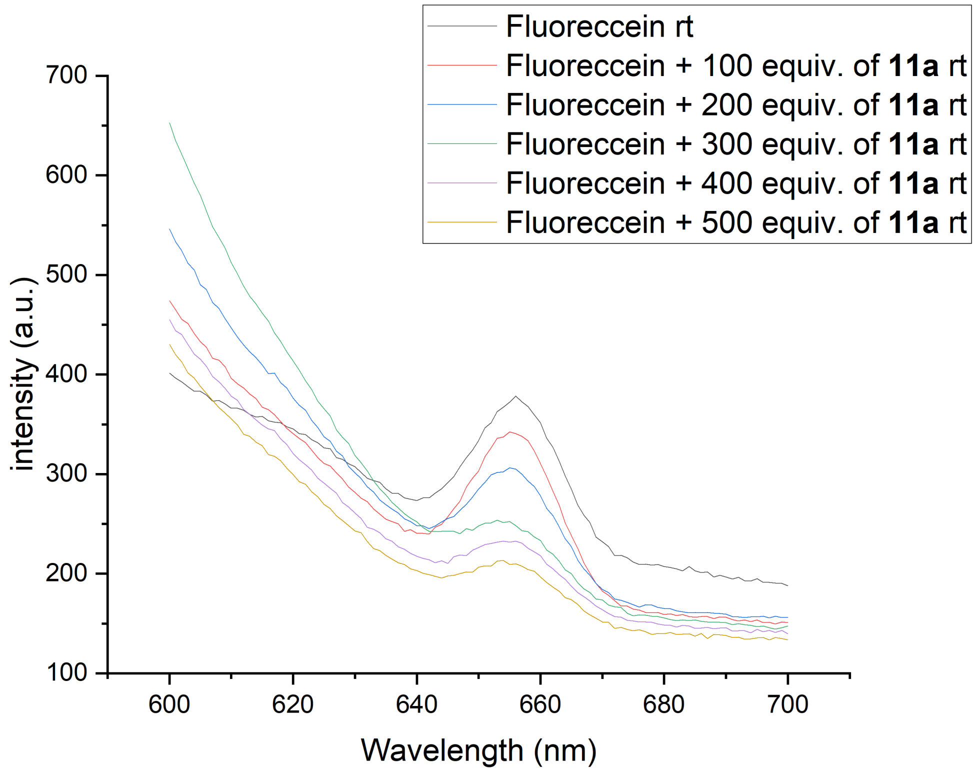


**Figure S3:** Fluorescence quenching with **11a** at rt.

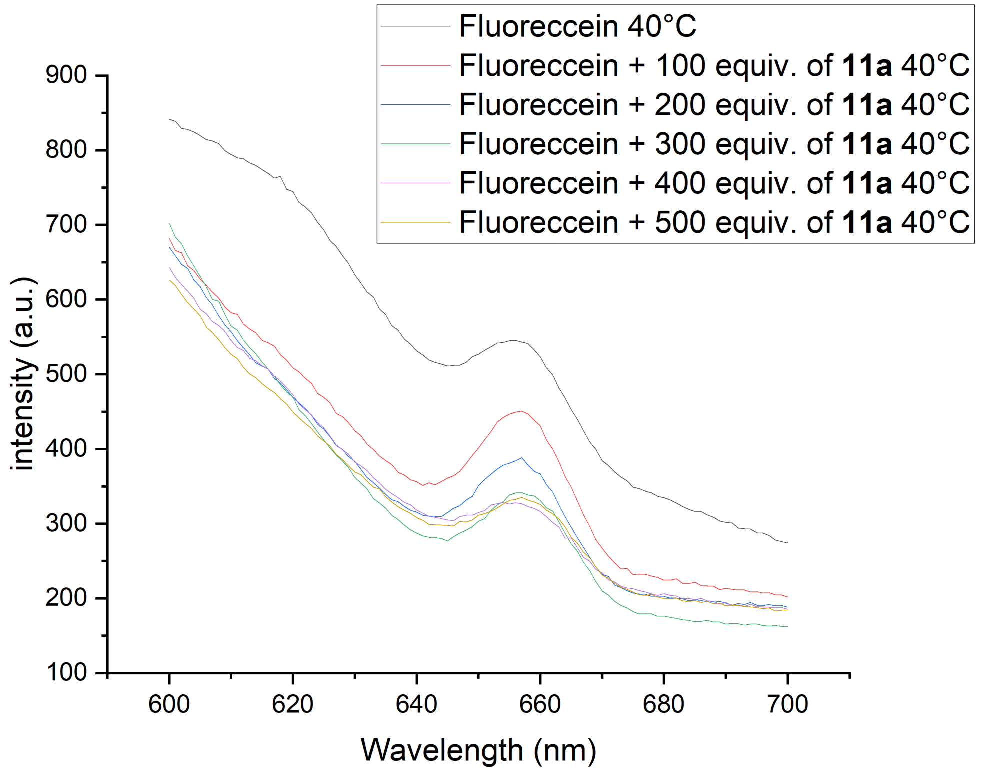


**Figure S4:** Fluorescence quenching with **11a** at 40 °C.

**Stern-Volmer plots**

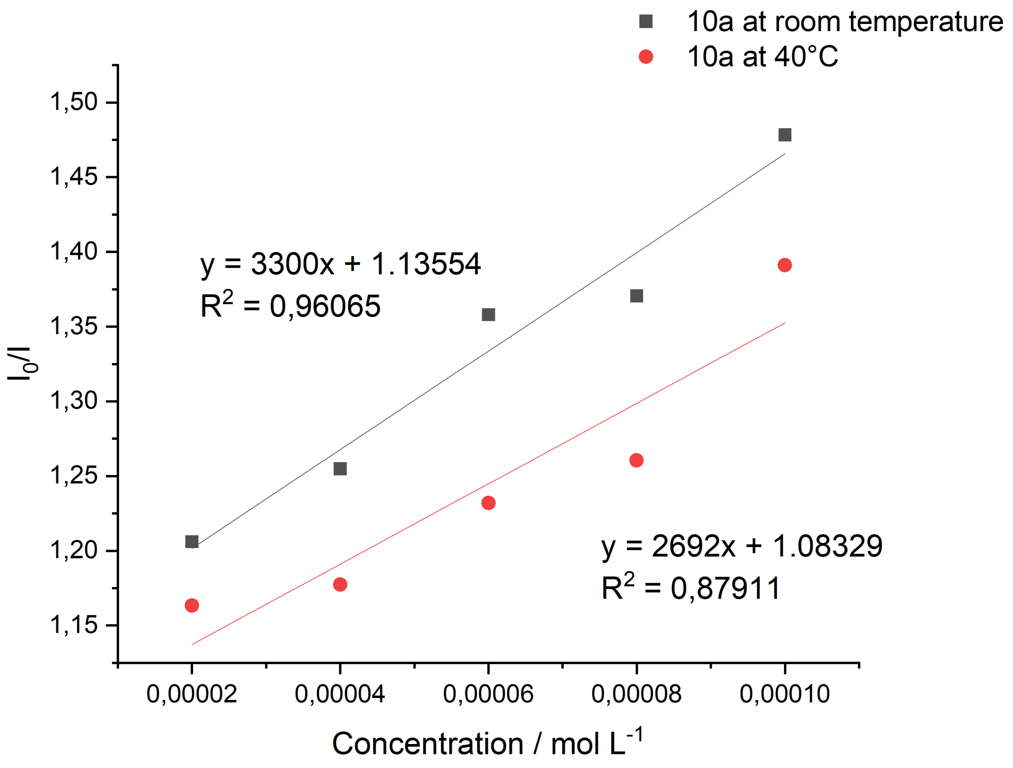


**Figure S5:** Stern-Volmer plot of with **10a**

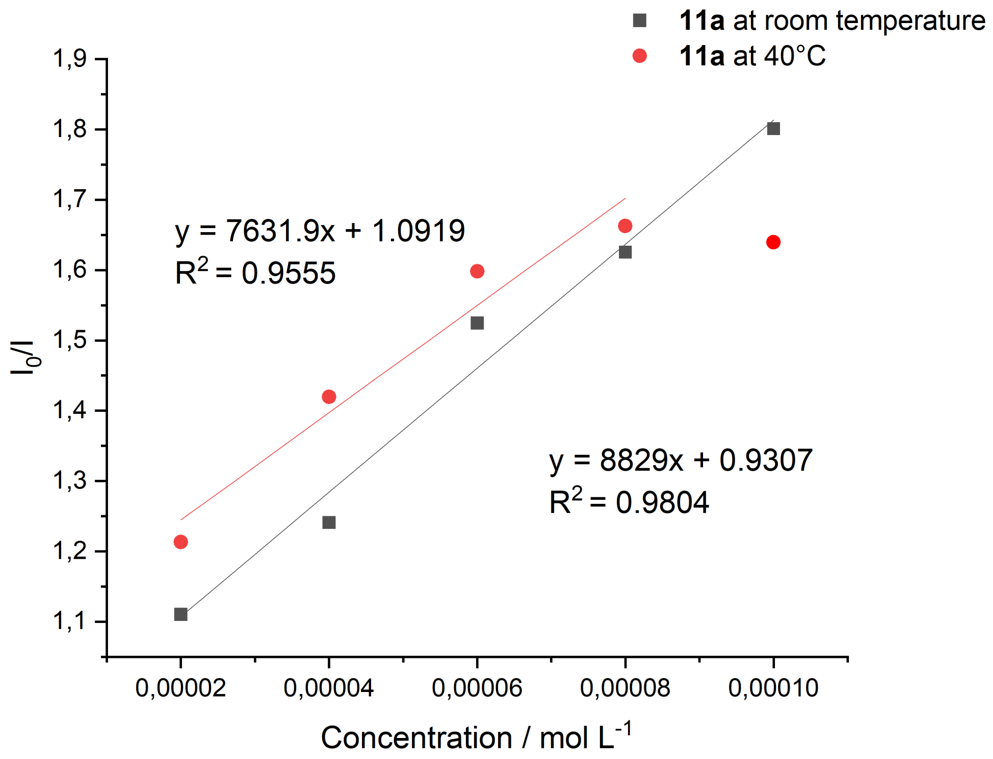


**Figure S6:** Stern-Volmer plot of with **11a**

The reported excited-state lifetime for Fluorescein acetonitrile (3.95 ns) was used for $K_q$ calculations.[7]

**Table S2:** Calculated $K_q$ values for **10a** and **11a**.

| Entry | Name | $K_{sv}$ $M^{-1}$ (Slope) | intersept | $K_q$ $_{10}{}^{12}$ $M^{-1}$ $s^{-1}$ |
|---|---|---|---|---|
| 1 | **10a** rt | *3300* | 1.135 | *0.8* |
| 2 | **10a** 40 °C | *2692* | 1.083 | *0.6* |
| 3 | **11a** rt | *8829* | 0.930 | *2.2* |
| 4 | **11a** 40 °C | *7632* | 1.178 | *1.9* |

## 5. UV-Vis experiment

Absorption experiments were performed on a Simazu UV-1900i UV-VIS Spectrophotometer. All the measurements were carried out with Fluorescein solutions concentration $10^{-3}$ M using dry degassed acetone in a 1.0 cm quartz cuvette. The experiment was performed to observe influence of **11a** on absorption properties of Fluorescein solution.

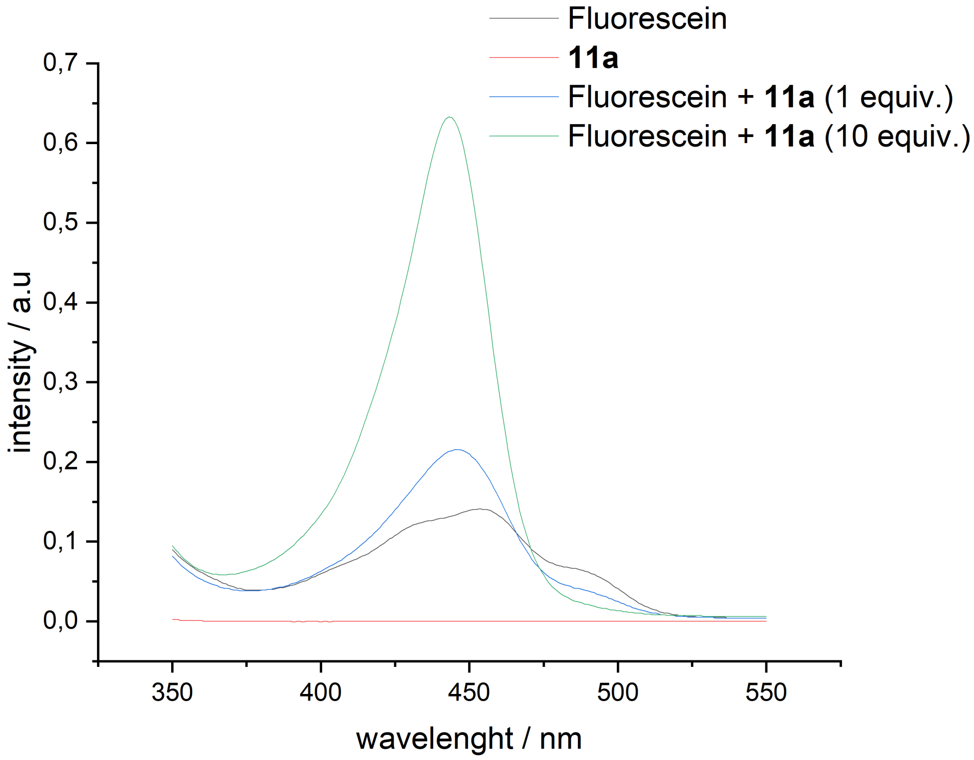


**Figure S7:** Absorption spectra of Fluorescein in acetone in presence of **11a**.

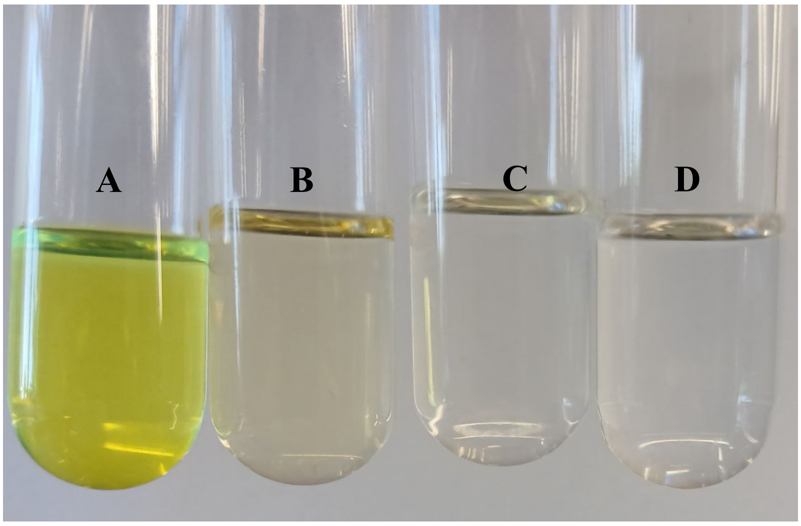


**Figure S8:** Formation of EDA complex **A**) Fluorescein ($c = 10^{-4}$ M) + 10 equiv. of **11a**, **B**) Fluorescein ($c = 10^{-4}$ M), **C**) **11a** ($c = 10^{-3}$ M), **D**) Acetonitrile.

## 6. Quantum Yield Calculation[8]

A ferrioxalate actinometer solution was prepared by following the Hammond variation of the Hatchard and Parker procedure[9] outlined in the Handbook of Photochemistry.[10] Ferrioxalate actinometer solution measures the decomposition of Fe(III) to Fe(II) ions, which are complexed by 1,10-phenanthroline and monitored by UV/Vis absorbance at 510 nm. The number of moles of Fe(II)-phenanthroline complex formed are directly proportional to moles of photons absorbed. The values of the quantum yield of potassium ferrioxalate are related to concentration and wavelength.
The solutions were prepared and stored in the dark (red light):
1. 0.012M Potassium ferrioxalate solution: 147.4 mg of potassium ferrioxalate (commercially available from Alfa Aesar) and 69.5 µL of sulfuric acid (96%) were added to a 25 mL volumetric flask and filled to the mark with HPLC grade water.
2. Phenanthroline solution: 100 mg of 1,10-phenanthroline in a 50 mL volumetric flask, filled to the mark with HPLC grade water (0.2% by weight).
3. Buffer solution: To a 100 mL volumetric flask 4.94 g of NaOAc and 1.0 mL of sulfuric acid (96%) were added and filled to the mark with HPLC grade water.
Reaction setup:

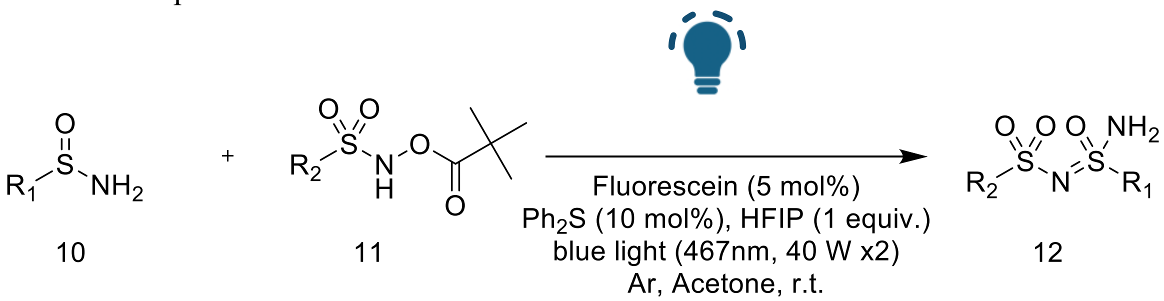


1. Reaction solution: To a test tube with **10** (0.1 mmol, 1.0 equiv. **11** (0.2 mmol, 2.0 equiv.) and Fluorescein (5 mol%), was degassed three times with Ar. After it HFIP (0.1 mmol, 1.0 equiv.) and Diphenyl sulfide (0.01 mmol, 0.1 equiv.) dissolved in 1 mL dry Acetone was added to the test tube via syringe. The test tube was irradiated with 2 x 40 W (467 nm) blue LEDs at a distance of ca. 4.5 cm (with cooling by the fan) for the required time. Three different reactions were set up and irradiated for different times: 20 min, 40 min and 60 min. The crude reaction mixture was analyzed by 1H NMR using mesitylene as an internal standard to calculate the moles of product after each irradiation.
2. Actinometer solutions: A test tube of the same dimensions as used for the reaction mixtures was loaded with 0.2 mL of actinometer solution and placed on the HP-LED the same light intensity as the reaction (without freeze-pump-thaw). Three different actinometer solutions were irradiated in sequence for 2 s, 4 s, 6 s and 8 s. To irradiate the Schlenk tube, it was placed on the holder with the light off and the light was turned on for the desired time. After each irradiation the actinometer solutions were carefully transferred into a 10 mL volumetric flask, then 0.5 mL of phenanthroline solution and 2.0 mL of buffer solution were added and the flask was filled up with water. The mixture was then analysed by UV-Vis spectroscopy (**Figure S9**).

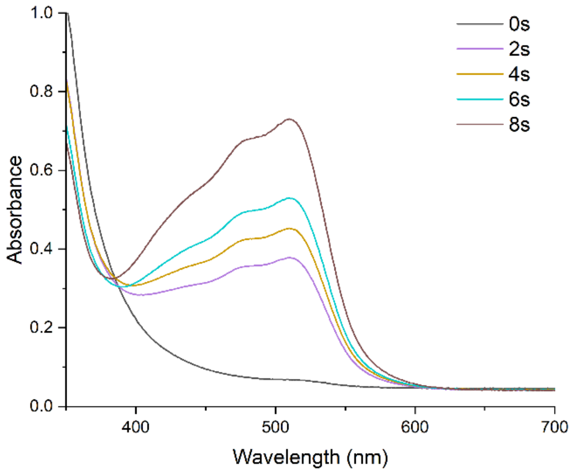


**Figure S9**: UV-Vis recorded spectra of the actinometer solutions irradiated for different periods of times.

The moles of Fe(II) formed for each sample are determined using Beers' Law:

$$n(Fe^{II}) = \frac{V_1 \,.\, V_3 \,.\, \Delta A(510\text{ nm})}{10^3 \,.\, l \,.\, V_2 \,.\varepsilon(510\text{ nm})}$$

$n(Fe^{II})$ = moles of $Fe^{II}$
$V_1$ = irradiated volume (0.2 mL)
$V_2$ = aliquot of the irradiated solution taken (0.2 mL)
$V_3$ = final volume after complexation (10 mL)
l = optical path-length of the irradiation cell (1 cm)
ΔA(510 nm) = optical difference in absorbance of the actinometer irradiated at 510 nm vs. dark
ε(510 nm) = extinction coefficient for $[Fe(phen)_3]^{2+}$ at 510 nm (11100 L $mol^{-1}$ $cm^{1}$)

The moles of Fe(II) formed (y-axis) are plotted as a function of time (x-axis) (**Figure S10**).

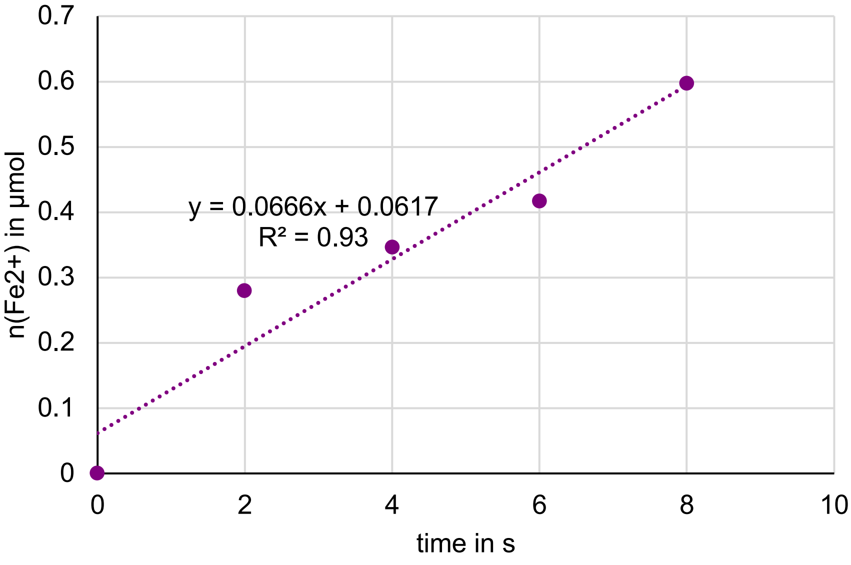


**Figure S10**: Plot of the moles of Fe(II) generated from the irradiation of the actinometer solutions against time.

The slope of this line was correlated to the moles of incident photons by unit of time (or photon flux) ($q^0_{n,p}$) using the following equation:

$$\Phi(\lambda) = \frac{dx / dt}{q^0_{n,p}\ [1 - 10^{-A(\lambda)}]}$$

$\Phi(\lambda)$ = quantum yield for the formation of Fe(II) = 1.11
dx/dt = change of a measurable quantity (spectral or any other property)
$[1-10^{-A(\lambda)}]$ = ratio of absorbed photons by the solution
$A(\lambda)$ = absorbance of the inspected species for the experiments at (467 nm). Actinometer = 0.068; reaction mixture = 0.411

This yields a photon flux $q^0_{n,p} = 3.546 \cdot 10^{-7}$ einstein $s^{-1}$.
Using the measured photon flux, the quantum yield can be determined by plotting the moles of product (y-axis) against the number of photons (x-axis) at different time intervals (**Figure S11**).

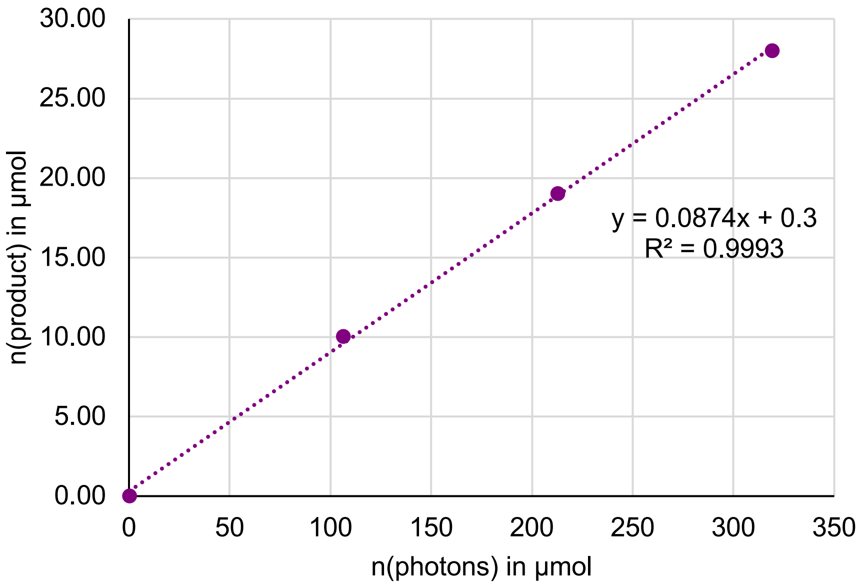


**Figure S11**: Plot of the moles of products generated from the irradiation of the reaction solutions, against the moles of photons absorbed by each reaction solution.
The slope of the linear regression is equal to:

$$dx / dt = \Phi(\lambda)\ [1 - 10^{-A(467\ nm)}]$$

This allows for the determination of the quantum yield which is calculated to be **Φ(467 nm) = 0.143.**

## 7. Cyclic voltammetry measurements

Cyclic voltammetry was measured on a Metrohm Autolab analyzer using a standard three- electrode cell configuration. A platinum working electrode was employed alongside a platinum wire counter electrode and an Ag/AgCl reference electrode. All the solutions were degassed by bubbling Ar prior to measurements. 5 mM solutions of the desired compounds were freshly prepared in dry SPS grade acetonitrile along with 0.1 M of tetrabutylammonium hexafluorophosphate (*n*-$Bu_4NPF_6$) as supporting electrolyte and were examined at a scan rate of 0.1 V $s^{-1}$. Solutions were kept under positive pressure of nitrogen during all the measurements. Potential values are given versus the $Ag/Ag^+$.

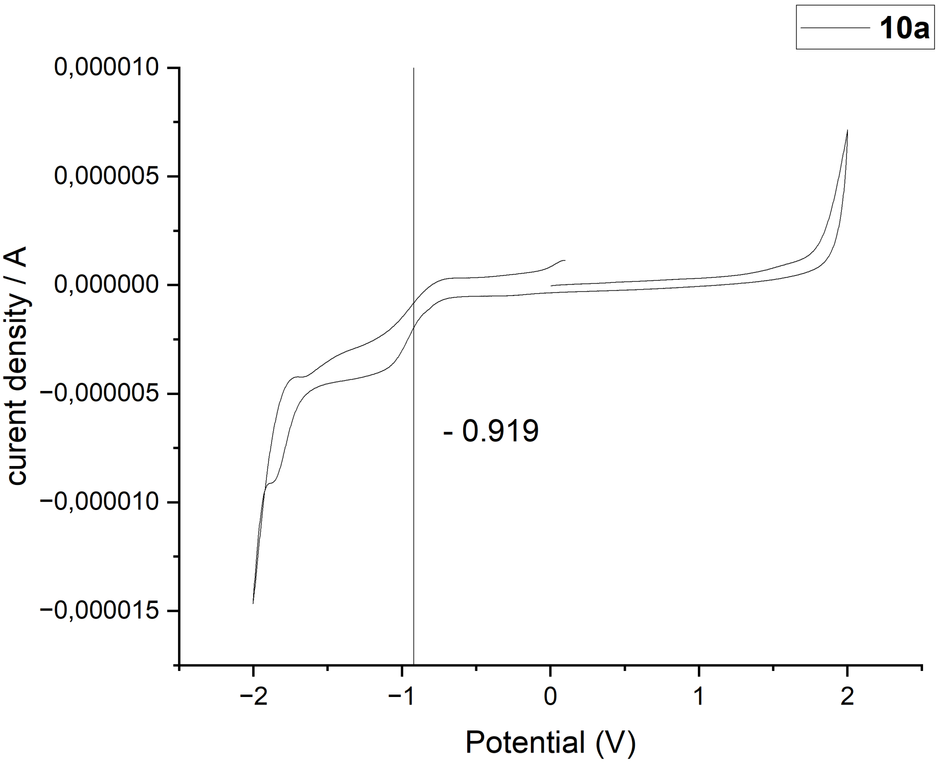


**Figure S12:** 0.005 M solution of **10a** were analysed in 0.1 M MeCN solution of $NBu_4PF_6$ used as electrolyte

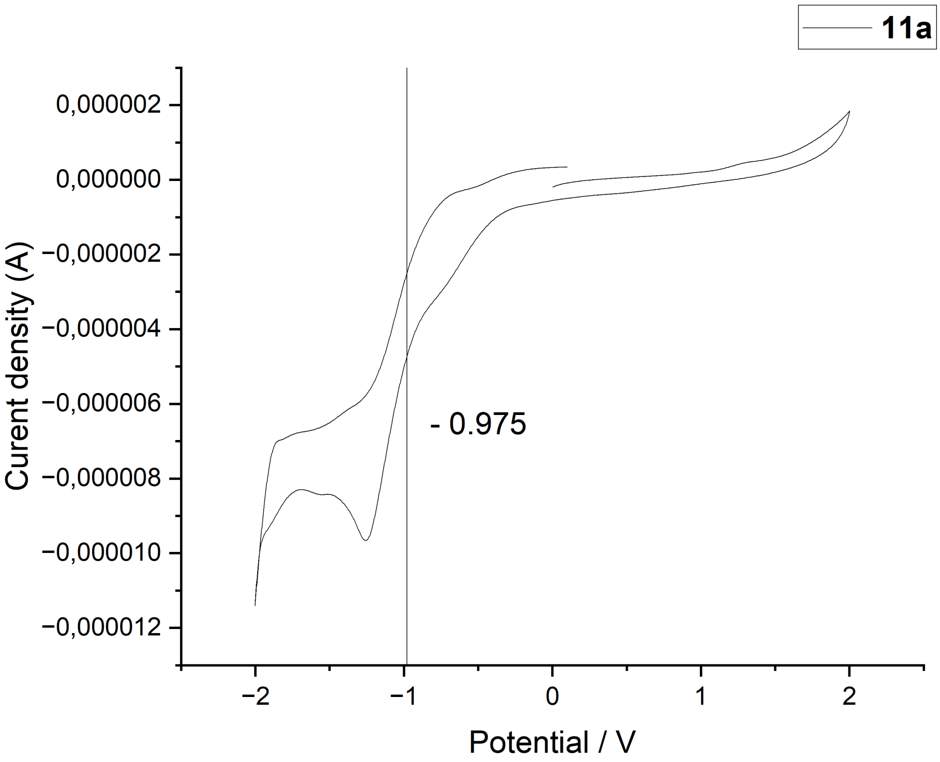


**Figure S13:** 0.005 M solution of **11a** were analysed in 0.1 M MeCN solution of $NBu_4PF_6$ used as electrolyte.

# 8. Radical Trapping Experiment

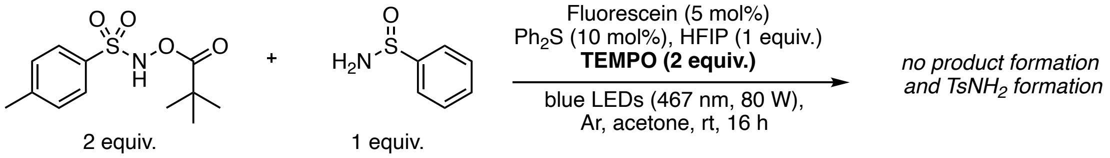


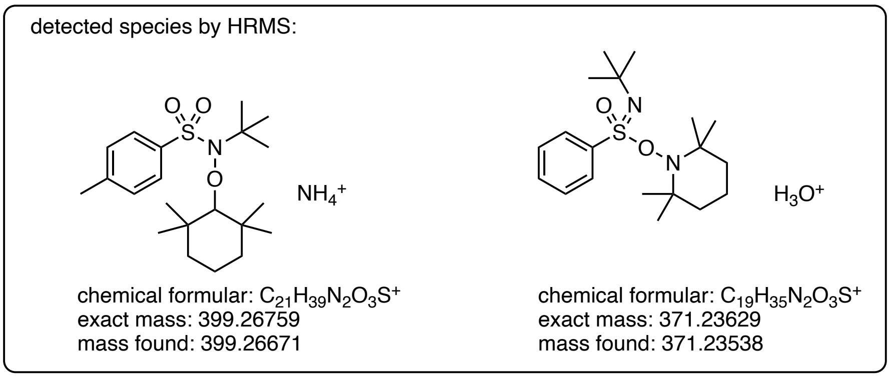


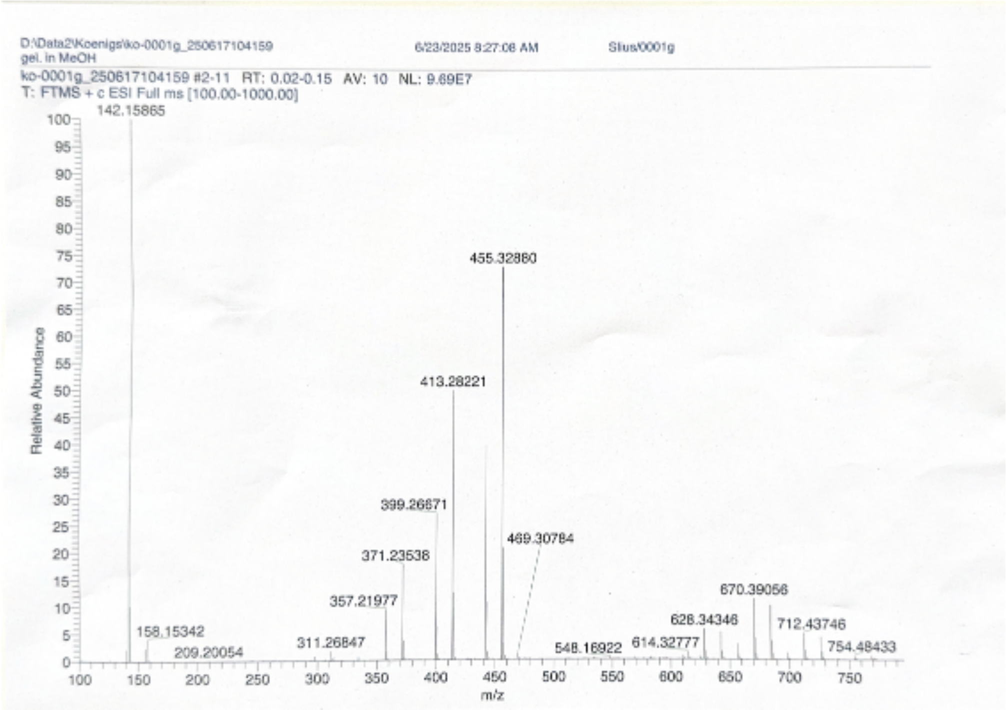


**Figure S14:** HRMS analysis of the crude reaction mixture.

## 9. Incompatible Substrates

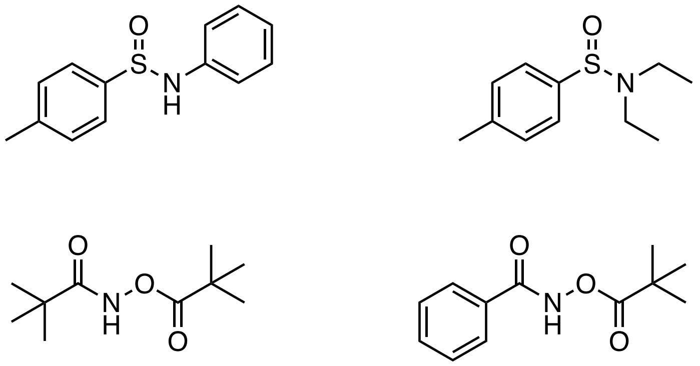

**Figure S15:** Incompatible substrates under standard reaction conditions.

## 10. Crystallographic studies

Crystallographic data, parameters and refinement results are listed in **Table S3**. Intensity data was collected on a STOE STADIVARI 4-circle diffractometer with a DECTRIS PILATUS 200K hybrid pixel counting detector in ω-scan mode with Mo-Kα radiation from a Xenocs Mo microfocus tube with multilayer optics. An Oxford Cryostream 800 was used to maintain the measuring temperature. Data was integrated using *INTEGRATE3D* and *X-Area*.[11] An absorption correction was performed using multiscan methods in *LANA*.[11] Structure solution was obtained using intrinsic phasing with *SHELXT*[12] and full least-squares refinements based on $F^2$ were performed using the *olex2.refine* algorithm.[13] Non-spherical refinement was performed with *NoSpherA2*[13] using the r2scan functional[15] and a triple-ζ basis set (def2-TZVP) in *ORCA6.0*.[16-20] Crystal structures were visualized using *Olex2*.[13] Additional crystallographic information can be found in **Table S3.**

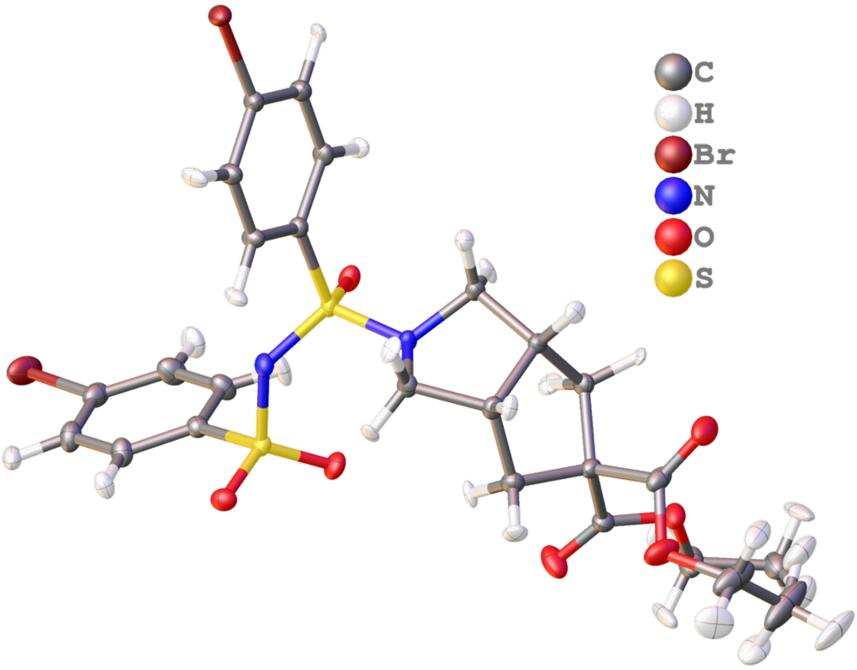


**Figure S16**: Asymmetric unit of ***Diethyl(3a*R*,6a*S*)-2-(4-bromo-*N*-((4-bromophenyl)sulfonyl)phenylsulfonimidoyl)hexahydrocyclopenta[*c*]pyrrole-5,5(1*H*)-dicarboxylate*** (**20d**). ADPs of heavy atoms are scaled to 50 % probability. Compound **12i** crystallizes in the monoclinic space group *P2₁/c* with Z = 4. Structure factors for non-spherical refinement were calculated with NoSpherA2. This allows for more accurate atomic positions, especially for hydrogen as well as ADPs for hydrogen. CCDC: 2443300

**Table S3**: Crystallographic data for **20d**.

| | |
|---|---|
| Identification code | **20d** |
| CCDC identifier | 2443300 |
| Empirical formula | $C_{25}H_{28}Br_2N_2O_7S_2$ |
| Formula weight | 692.449 |
| Temperature / K | 100(2) |
| Crystal system | monoclinic |
| Space group | $P2_1/c$ |
| *a* / Å | 11.1951(3) |
| *b* / Å | 11.1720(1) |
| *c* / Å | 22.0531(4) |
| *α* / ° | 90 |
| *β* / ° | 100.050(2) |
| *γ* / ° | 90 |
| Volume / $Å^3$ | 2715.90(9) |
| *Z* | 4 |
| $\rho_{calc}$ / $g/cm^3$ | 1.693 |
| $\mu$ / $mm^{-1}$ | 3.195 |
| *F*(000) | 1400.2 |
| Crystal size / $mm^3$ | 0.21 × 0.173 × 0.15 |
| Radiation | Mo Kα (λ = 0.71073) |
| $2\Theta$ range for data collection / ° | 4.78 to 58 |
| Index ranges | $-15 \le h \le 15$, $-15 \le k \le 14$, $-24 \le l \le 30$ |
| Reflections collected | 92687 |
| Independent reflections | 7212 [$R_{int}$ = 0.0408, $R_{sigma}$ = 0.0186] |
| Data/restraints/parameters | 7212/18/630 |
| Goodness-of-fit on $F^2$ | 1.050 |
| Final R indexes [I>=2σ (I)] | $R_1$ = 0.0232, $wR_2$ = 0.0498 |
| Final R indexes [all data] | $R_1$ = 0.0328, $wR_2$ = 0.0527 |
| Largest diff. peak/hole / e $Å^{-3}$ | 0.97/-0.40 |
| NoSpherA2 level of theory | r2scan, def2-TZVP |

## 11. HPLC Data

***N*-(Amino(*tert*-butyl)(oxo)-λ⁶-sulfaneylidene)-4-methylbenzenesulfonamide (12w)**

**Racemic mixture**

ADH, *n*-hexane/2-propanol = 90/10, flow rate = 0.75 mL/min, λ = 220 nm, temp = rt

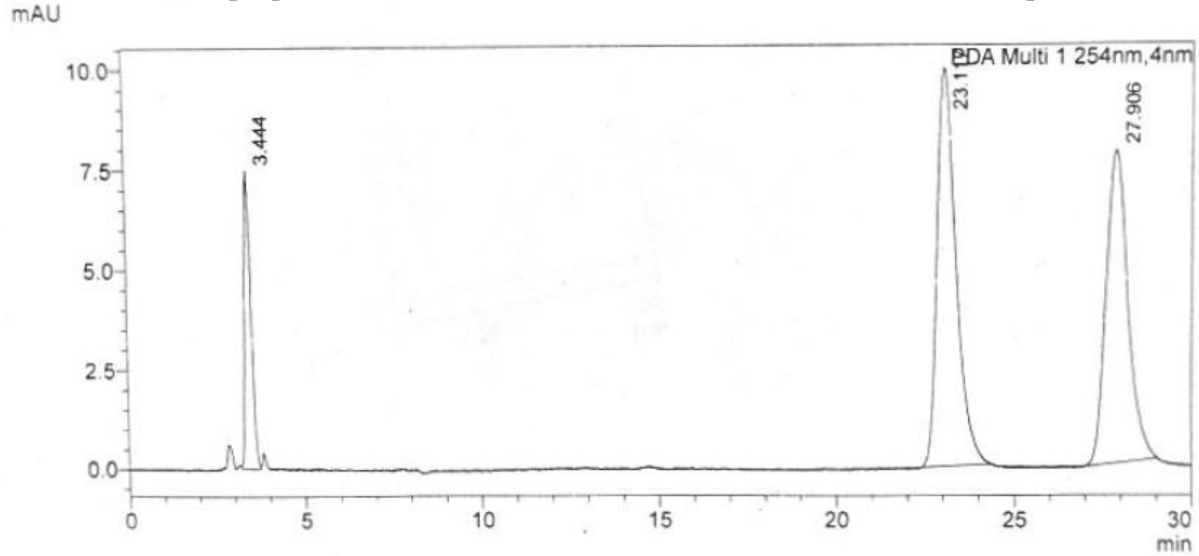


| Peak | Ret. Time | Area | Height | Height% | Area% |
|---|---|---|---|---|---|
| 1 | 3.444 | 93798 | 7455 | 29.652 | 11.875 |
| 2 | 23.117 | 362905 | 9909 | 39.416 | 45.944 |
| 3 | 27.906 | 333177 | 7777 | 30.933 | 42.181 |
| Total | | 789881 | 25141 | 100.000 | 100.000 |

**(*R*)-*N*-(amino(tert-butyl)(oxo)-$\lambda^6$-sulfaneylidene)-4-methylbenzenesulfonamide (12w)**

***R*– isomer**

ADH, *n*-hexane/2-propanol = 90/10, flow rate = 0.75 mL/min, λ = 220 nm, temp = rt

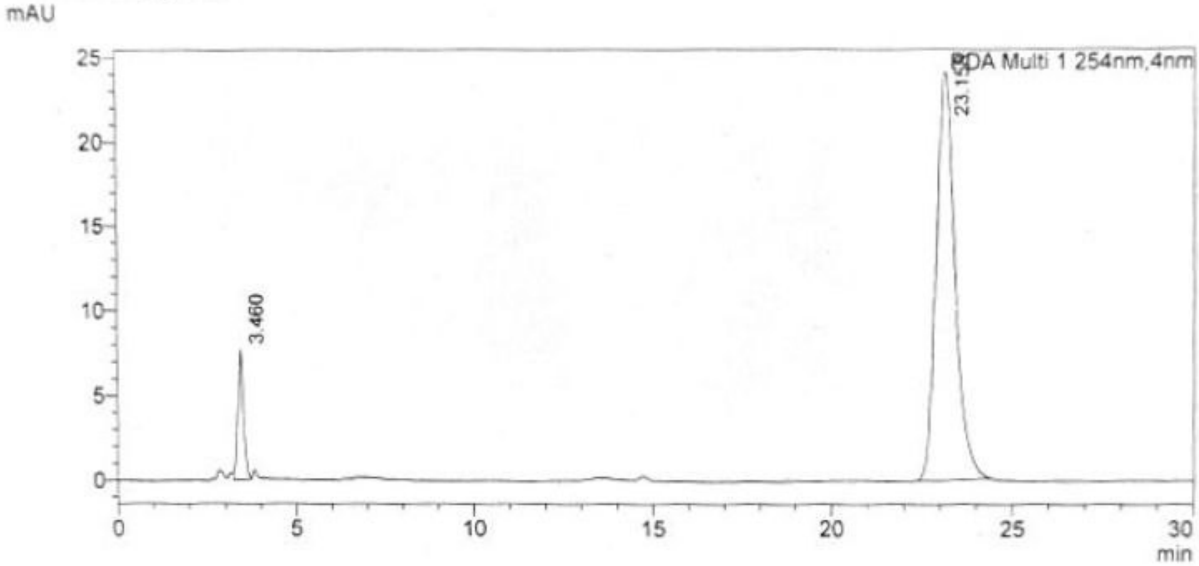


| Peak | Ret. Time | Area | Height | Height% | Area% |
|---|---|---|---|---|---|
| 1 | 3.460 | 82777 | 7646 | 24.170 | 8.677 |
| 2 | 23.158 | 871253 | 23989 | 75.830 | 91.323 |
| Total | | 954030 | 31635 | 100.000 | 100.000 |

## 12. Physical Data

### *N*-(Pivaloyloxy)-4-(5-(*p*-tolyl)-3-(trifluoromethyl)-1*H*-pyrazol-1-yl) benzenesulfonamide

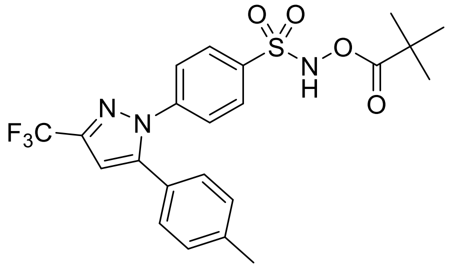

The title compound was synthesized according to the general procedure (GP-3) and was obtained after silica column chromatography in DCM as a yellow solid (42%, 500 mg).
**$^1$H NMR** (600 MHz, Chloroform-*d*): δ = 9.03 (s, 1H), 7.91 (d, *J* = 8.8 Hz, 2H), 7.53 (d, *J* = 8.7 Hz, 2H), 7.19 (d, *J* = 7.5 Hz, 2H), 7.09 (d, *J* = 8.2 Hz, 2H), 6.74 (s, 1H), 2.39 (s, 3H), 1.15 (s, 9H) ppm.
**$^{13}$C NMR** (151 MHz, Chloroform-*d*): δ = 176.5, 145.3, 144.4 (q, *J* = 38.8 Hz), 143.9, 139.9, 134.7, 129.87, 129.83, 128.7, 125.6, 125.2, 120.9 (q, *J* = 269.4 Hz), 106.7, 26.7, 21.3 ppm.
**$^{19}$F NMR** (564 MHz, $CDCl_3$): δ = -62.57 ppm.
**HRMS** (ESI): *m/z*: $[M + Na]^+$ Calcd. for $C_{22}H_{22}F_3N_3O_4SNa^+$: 504.11753; Found: 504.11831.

### *N*-(Amino(oxo)(phenyl)- λ$^6$-sulfaneylidene)-4-methylbenzenesulfonamide

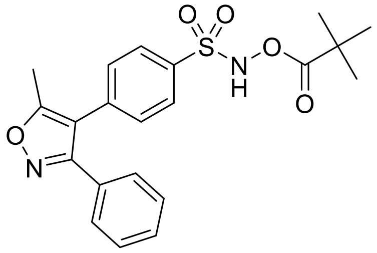

The title compound was synthesized according to the general procedure (GP-3) and was obtained after silica column chromatography in DCM as a yellow solid (62%, 640 mg).
**$^1$H NMR** (600 MHz, DMSO-*d*$_6$): δ = 11.19 (s, 1H), 7.88 (d, *J* = 8.5 Hz, 2H), 7.51 (d, *J* = 8.4 Hz, 2H), 7.47 – 7.43 (m, 1H), 7.37 (t, *J* = 7.8 Hz, 2H), 7.32 (d, *J* = 1.1 Hz, 1H), 7.30 (d, *J* = 1.5 Hz, 1H), 2.46 (s, 3H), 1.07 (s, 9H) ppm.
**$^{13}$C NMR** (151 MHz, DMSO-*d*$_6$): δ = 174.5, 168.3, 161.0, 136.1, 136.0, 130.8, 130.3, 129.2, 129.1, 128.6, 128.6, 114.4, 27.0, 11.8 ppm.
**HRMS** (ESI): *m/z*: $[M + Na]^+$ Calcd. for $C_{21}H_{22}N_2O_5SNa^+$: 437.11416; Found: 437.11382.

**4-(5-Methyl-3-phenylisoxazol-4-yl)benzenesulfinamide**

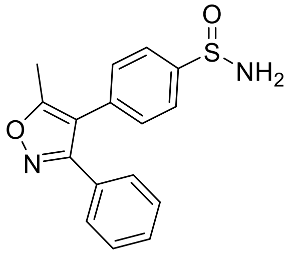

The title compound was synthesized according to the literature.[21]. To a water solution of sulfochloride (1 g, 1.0 equiv.), Sodium sulfite (0.76 g, 2.0 equiv.) and Sodium bicarbonate (0.5 g, 2.0 equiv.) was added. Equipped with reflux condenser, and heat 80 ° C for 4 h. Then, water was dried with addition of toluene on vacuum. The residue was washed with hot methanol. The solution was dried on high vacuum. After this the residue was suspended in THF and Oxalyl chloride (0.63 ml, 2.5 equkiv.) at 0 °C was added. After stirring for 1 hour at 20 °C, the reaction mixture was added into an aqueous ammoniak solution (50 mL, 25%) at 0 °C. The reaction mixture was stirred at 20 °C for 1 hour and then concentrated in vacuo. The residue was purified by column chromatography (*n*-hexane : ethyl acetate – 4:1) to obtain a yellow solid (42%, 380 mg).

$^1$**H NMR** (600 MHz, Chloroform-*d*): δ = 7.75 (d, *J* = 8.4 Hz, 2H), 7.42 – 7.35 (m, 3H), 7.35 – 7.29 (m, 4H), 4.43 (s, 2H), 2.47 (s, 3H) ppm.

$^{13}$**C NMR** (151 MHz, Chloroform-*d*): δ = 167.0, 161.1, 145.7, 133.5, 130.2, 129.6, 128.65, 128.63, 128.4, 125.9, 114.7, 11.7 ppm.

**HRMS** (ESI): *m/z*: $[M + Na]^+$ Calcd. for $C_{16}H_{15}N_2O_2SNa^+$: 299.08487; Found: 299.08431.

***N*-(Amino(oxo)(phenyl)- λ$^6$-sulfaneylidene)-4-methylbenzenesulfonamide (12a)**

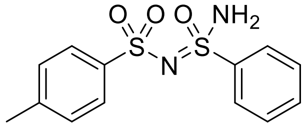

The title compound **12a** was synthesized according to the general procedure (GP-4), and was obtained after silica column chromatography (DCM : Methanol/$NH_3$ – 40:1 – 20:1 – 9:1) as a yellow solid (95%, 59 mg).

$^1$**H NMR** (600 MHz, DMSO-*d*$_6$): δ = 8.07 (s, 2H), 7.85 – 7.73 (m, 2H), 7.71 – 7.60 (m, 1H), 7.60 – 7.47 (m, 4H), 7.24 (d, *J* = 8.5 Hz, 2H), 2.33 (s, 3H) ppm.

$^{13}$**C NMR** (151 MHz, DMSO-*d*$_6$): δ = 142.7, 142.3, 141.5, 133.2, 129.4, 129.4, 126.9, 126.43, 21.3 ppm.

**HRMS** (ESI): *m/z*: $[M + Na]^+$ Calcd. for $C_{13}H_{14}N_2O_3S_2Na^+$: 333.03380; Found: 333.03362.

**_N_-(Amino(oxo)(phenyl)- λ[6]-sulfaneylidene)benzenesulfonamide (12b)**

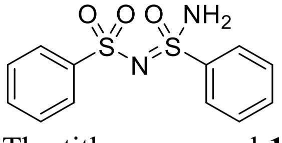


The title compound **12b** was synthesized according to the general procedure (GP-4), and was obtained after silica column chromatography (DCM : Methanol/$NH_3$ – 40:1 – 20:1 – 9:1) as a yellow solid (71%, 42 mg).
$^{1}$**H NMR** (600 MHz, DMSO-$d_6$): δ = 8.12 (s, 2H), 7.78 (dd, *J* = 8.4, 1.3 Hz, 2H), 7.68 – 7.60 (m, 3H), 7.53 (ddd, *J* = 9.1, 8.1, 7.1 Hz, 3H), 7.45 (dd, *J* = 8.4, 7.1 Hz, 2H) ppm.
$^{13}$**C NMR** (151 MHz, DMSO-$d_6$): δ = 144.1, 142.5, 133.3, 132.2, 129.4, 129.1, 126.9, 126.3 ppm.
**HRMS** (ESI): *m/z*: $[M]^+$ Calcd. for $C_{12}H_{12}N_2O_3S_2Na^+$: 319.01816; Found: 319.01857.

**_N_-(Amino(oxo)(phenyl)- λ[6]-sulfaneylidene)-4-fluorobenzenesulfonamide (12c)**

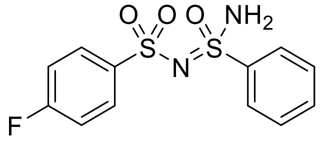


The title compound **12c** was synthesized according to the general procedure (GP-4), and was obtained after silica column chromatography (DCM : Methanol/$NH_3$ – 40:1 – 20:1 – 9:1) as a yellow solid (48%, 30 mg).
$^{1}$**H NMR** (400 MHz, DMSO-$d_6$): δ = 8.14 (s, 2H), 7.76 (d, *J* = 7.7 Hz, 2H), 7.72 – 7.58 (m, 3H), 7.53 (t, *J* = 7.6 Hz, 2H), 7.26 (t, *J* = 8.9 Hz, 2H) ppm.
$^{13}$**C NMR** (151 MHz, DMSO-$d_6$): δ = 164.1 (d, *J* = 249.8 Hz), 142.3, 140.5, 133.4, 129.4, 129.3 (d, *J* = 9.9 Hz), 127.0, 116.1 (d, *J* = 23.0 Hz) ppm.
$^{19}$**F NMR** (564 MHz, DMSO-$d_6$): δ = -107.99 (ddd, *J* = 14.1, 8.9, 5.2 Hz) ppm.
**HRMS** (ESI): *m/z*: $[M + Na]^+$ Calcd. for $C_{12}H_{11}N_2O_3FS_2Na^+$: 337.00873; Found: 337.00912.

**_N_-(Amino(oxo)(phenyl)- λ[6]-sulfaneylidene)-4-bromobenzenesulfonamide (12d)**

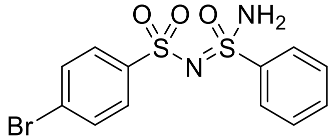


The title compound **12d** was synthesized according to the general procedure (GP-4), and was obtained after silica column chromatography (DCM : Methanol/$NH_3$ – 40:1 – 20:1 – 9:1) as a yellow solid (80%, 60 mg).
$^{1}$**H NMR** (600 MHz, DMSO-$d_6$): δ = 8.20 (s, 2H), 7.78 (dd, *J* = 8.5, 1.3 Hz, 2H), 7.68 – 7.62 (m, 3H), 7.61 – 7.50 (m, 4H) ppm.
$^{13}$**C NMR** (151 MHz, DMSO-$d_6$): δ = 143.3, 142.2, 133.4, 132.1, 129.5, 128.5, 127.0, 125.9 ppm.
**HRMS** (ESI): *m/z*: $[M + Na]^+$ Calcd. for $C_{12}H_{11}N_2O_3BrS_2Na^+$: 396.92867; Found: 396.92933.

**N-(Amino(oxo)(phenyl)-λ⁶-sulfaneylidene)-4-(trifluoromethyl) benzenesulfonamide (12e)**

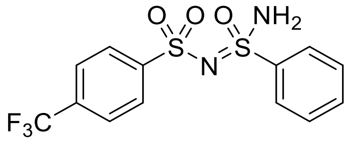

The title compound **12e** was synthesized according to the general procedure (GP-4), and was obtained after silica column chromatography (DCM : Methanol/$NH_3$ – 40:1 – 20:1 – 9:1) as a yellow solid (71%, 52 mg).

**$^1$H NMR** (600 MHz, DMSO-$d_6$): δ = 8.27 (s, 2H), 7.84 (q, $J$ = 8.6 Hz, 4H), 7.77 (dd, $J$ = 8.3, 1.1 Hz, 2H), 7.64 – 7.60 (m, 1H), 7.55 – 7.51 (m, 2H) ppm.

**$^{13}$C NMR** (151 MHz, DMSO-$d_6$): δ = 147.6, 141.9, 133.5, 132.0 (q, $J$ = 32.2 Hz), 129.5, 127.4, 127.0, 126.3 (q, $J$ = 4.1 Hz), 124.0 (q, $J$ = 272.5 Hz), 118.4 ppm.

**$^{19}$F NMR** (564 MHz, DMSO-$d_6$): δ = -61.62 ppm.

**HRMS** (ESI): *m/z*: $[M + Na]^+$ Calcd. for $C_{15}H_{11}N_2O_3F_3S_2Na^+$: 387.00554; Found: 387.00527.

**N-(Amino(oxo)(phenyl)-λ⁶-sulfaneylidene)-3-chlorobenzenesulfonamide (12f)**

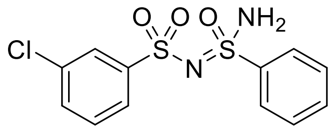

The title compound **12f** was synthesized according to the general procedure (GP-4), and was obtained after silica column chromatography (DCM : Methanol/$NH_3$ – 40:1 – 20:1 – 9:1) as a yellow solid (74%, 48 mg).

**$^1$H NMR** (600 MHz, DMSO-$d_6$): δ = 8.24 (s, 2H), 7.77 (d, $J$ = 7.4 Hz, 2H), 7.66 – 7.62 (m, 1H), 7.60 (dd, $J$ = 7.7, 1.7 Hz, 2H), 7.57 – 7.52 (m, 2H), 7.52 – 7.47 (m, 2H) ppm.

**$^{13}$C NMR** (151 MHz, DMSO-$d_6$): δ = 145.7, 141.8, 133.6, 133.5, 132.2, 131.3, 129.4, 127.0, 126.0, 125.1 ppm.

**HRMS** (ESI): *m/z*: $[M + Na]^+$ Calcd. for $C_{12}H_{11}N_2O_3ClS_2Na^+$: 352.97918; Found: 352.97900.

**N-(Amino(oxo)(phenyl)-λ⁶-sulfaneylidene)-3-nitrobenzenesulfonamide (12g)**

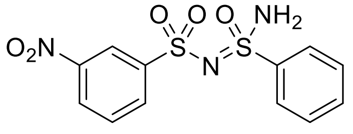

The title compound **12g** was synthesized according to the general procedure (GP-4), and was obtained after silica column chromatography (DCM : Methanol/$NH_3$ – 40:1 – 20:1 – 9:1) as a yellow solid (38%, 26 mg).

**$^1$H NMR** (600 MHz, DMSO-$d_6$): δ = 8.40 – 8.35 (m, 1H), 8.32 (s, 3H), 8.22 (t, $J$ = 2.0 Hz, 1H), 8.07 (d, $J$ = 8.1 Hz, 1H), 7.81 – 7.70 (m, 4H), 7.60 (t, $J$ = 7.4 Hz, 1H), 7.52 (t, $J$ = 7.7 Hz, 3H) ppm.

**$^{13}$C NMR** (151 MHz, DMSO-$d_6$): δ = 147ppm.8, 145.2, 141.5, 133.6, 132.6, 131.4, 129.5, 127.0, 126.9, 121.1 ppm.

**HRMS** (ESI): *m/z*: $[M + Na]^+$ Calcd. for $C_{12}H_{11}N_3O_5S_2Na^+$: 364.00323; Found: 364.00388.

**$N$-(Amino(oxo)(phenyl)-$\lambda^6$-sulfaneylidene)-3-chloro-2-methylbenzenesulfonamide (12h)**

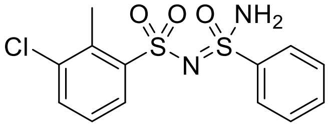


The title compound **12h** was synthesized according to the general procedure (GP-4), and was obtained after silica column chromatography (DCM : Methanol/$NH_3$ – 40:1 – 20:1 – 9:1) as a yellow solid (76%, 52 mg).

**$^1$H NMR** (600 MHz, DMSO-$d_6$): δ = 8.15 (s, 0H), 7.69 (d, $J$ = 7.6 Hz, 1H), 7.63 – 7.53 (m, 1H), 7.48 (t, $J$ = 7.8 Hz, 1H), 7.21 (t, $J$ = 8.0 Hz, 0H), 2.56 (s, 1H) ppm.

**$^{13}$C NMR** (151 MHz, DMSO-$d_6$): δ = 144.1, 141.6, 135.6, 134.4, 133.4, 132.9, 129.3, 127.2, 126.8, 126.7, 17.2 ppm.

**HRMS** (ESI): $m/z$: $[M + Na]^+$ Calcd. for $C_{13}H_{13}N_2O_3ClS_2Na^+$: 366.99483; Found: 366.99465.

**$N$-(Amino(4-bromophenyl)(oxo)-λ6-sulfaneylidene)-4-bromobenzenesulfonamide (12i)**

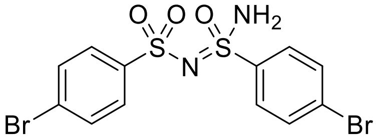


The title compound **12i** was synthesized according to the general procedure (GP-4), and was obtained after silica column chromatography (DCM : Methanol/$NH_3$ – 40:1 – 20:1 – 9:1) as a yellow solid (72%, 66 mg).

**$^1$H NMR** (600 MHz, DMSO-$d_6$): δ = 8.32 (s, 2H), 7.79 – 7.72 (m, 2H), 7.71 – 7.62 (m, 4H), 7.60 – 7.52 (m, 2H) ppm.

**$^{13}$C NMR** (151 MHz, DMSO-$d_6$): δ = 143.0, 141.4, 132.5, 132.2, 129.0, 128.5, 127.4, 126.1 ppm.

**HRMS** (ESI): $m/z$: $[M + H]^+$ Calcd. for $C_{12}H_{11}N_2O_3S_2Br_2^+$: 452.85724; Found: 452.85817.

**$N$-(Amino(oxo)(phenyl)-$\lambda^6$-sulfaneylidene)pyridine-3-sulfonamide (12j)**

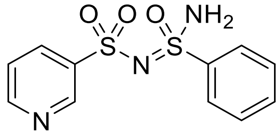


The title compound **12j** was synthesized according to the general procedure (GP-4), and was obtained after silica column chromatography (DCM : Methanol/$NH_3$ – 40:1 – 20:1 – 9:1) as a yellow solid (76%, 45 mg).

**$^1$H NMR** (600 MHz, DMSO-$d_6$): δ = 8.78 (s, 1H), 8.71 (d, $J$ = 5.2 Hz, 1H), 8.27 (s, 2H), 8.02 – 7.97 (m, 1H), 7.82 – 7.75 (m, 2H), 7.67 – 7.61 (m, 1H), 7.59 – 7.52 (m, 2H), 7.50 (dd, $J$ = 8.0, 4.8 Hz, 1H) ppm.

**$^{13}$C NMR** (151 MHz, DMSO-$d_6$): δ = 152.8, 147.0, 141.9, 140.2, 134.3, 133.6, 129.5, 127.0, 124.3 ppm.

**HRMS** (ESI): $m/z$: $[M + Na]^+$ Calcd. for $C_{11}H_{11}N_3O_3S_2Na^+$: 320.01340; Found: 320.01226.

**(*E*)-*N*-(amino(oxo)(phenyl)-λ⁶-sulfaneylidene)-2-phenylethene-1-sulfonamide (12k)**

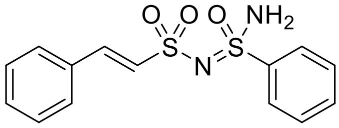

The title compound **12k** was synthesized according to the general procedure (GP-4), and was obtained after silica column chromatography (DCM : Methanol $NH_3$ – 40:1 – 20:1 – 9:1 ) as a yellow solid (71%, 46 mg).

$^1$**H NMR** (600 MHz, DMSO-$d_6$): δ = 8.14 (s, 2H), 7.92 – 7.85 (m, 2H), 7.62 – 7.52 (m, 5H), 7.41 – 7.36 (m, 3H), 7.10 (s, 2H) ppm.

$^{13}$**C NMR** (151 MHz, DMSO-$d_6$): δ = 142.5, 137.3, 133.3, 130.7, 130.6, 129.4, 129.3, 128.6, 127.0 ppm.

**HRMS** (ESI): *m/z*: [M + Na]$^+$ Calcd. for $C_{14}H_{14}N_2O_3S_2Na^+$: 345.03377; Found: 345.03268.

***N*-(Amino(oxo)(phenyl)-λ⁶-sulfaneylidene)methanesulfonamide (12l)**

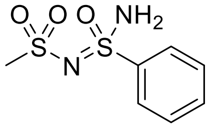

The title compound **12l** was synthesized according to the general procedure (GP-4), and was obtained after silica column chromatography (DCM : Methanol/$NH_3$ – 40:1 – 20:1 – 9:1) as a yellow solid (64%, 30 mg).

$^1$**H NMR** (600 MHz, DMSO-$d_6$): δ = 8.10 (s, 2H), 7.90 (d, *J* = 7.1 Hz, 2H), 7.66 (t, *J* = 7.3 Hz, 1H), 7.61 (t, *J* = 7.5 Hz, 2H), 2.94 (s, 3H) ppm.

$^{13}$**C NMR** (151 MHz, DMSO-$d_6$): δ =143.0, 133.3, 129.5, 126.8, 44.6 ppm.

**HRMS** (ESI): *m/z*: [M + Na]$^+$ Calcd. for $C_7H_{10}N_2O_3S_2Na^+$: 257.00250; Found: 257.00227.

***N*-(amino(oxo)(phenyl)- λ⁶-sulfaneylidene)-1-phenylmethanesulfonamide (12m)**

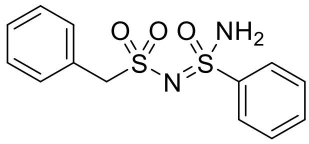

The title compound **12m** was synthesized according to the general procedure (GP-4), and was obtained after silica column chromatography (DCM : Methanol/$NH_3$ – 40:1 – 20:1 – 9:1) as a yellow solid (81%, 50 mg).

$^1$**H NMR** (600 MHz, Chloroform-*d*): δ = 7.97 – 7.87 (m, 2H), 7.61 – 7.55 (m, 1H), 7.51 – 7.45 (m, 2H), 7.45 – 7.38 (m, 2H), 7.35 – 7.28 (m, 3H), 5.29 (s, 2H), 4.41 (s, 2H) ppm.

$^{13}$**C NMR** (151 MHz Chloroform-*d*): δ = 140.5, 133.7, 131.1, 129.3, 129.2, 128.5, 126.8, 62.2 ppm.

**HRMS** (ESI): *m/z*: [M + Na]$^+$ Calcd. for $C_{13}H_{14}N_2O_3S_2Na^+$: 333.03380; Found: 333.03264.

**N-(Amino(oxo)(p-tolyl)-λ⁶-sulfaneylidene)-4-methylbenzenesulfonamide (12n)**

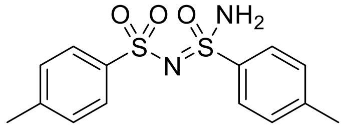


The title compound **12n** was synthesized according to the general procedure (GP-4), and was obtained after silica column chromatography (DCM : Methanol/$NH_3$ – 40:1 – 20:1 – 9:1) as a yellow solid (97%, 63 mg).

**$^1$H NMR** (600 MHz, DMSO-*d*$_6$): δ = 8.00 (s, 2H), 7.68 – 7.60 (m, 2H), 7.52 (d, *J* = 8.3 Hz, 2H), 7.33 (d, *J* = 8.1 Hz, 2H), 7.23 (d, *J* = 8.0 Hz, 2H), 2.36 (s, 3H), 2.33 (s, 3H) ppm.

**$^{13}$C NMR** (151 MHz, DMSO-*d*$_6$): δ = 143.7, 142.2, 141.5, 139.6, 129.7, 129.4, 127.0, 126.4, 21.4, 21.3 ppm.

**HRMS** (ESI): *m/z*: [M + Na]$^+$ Calcd. for $C_{15}H_{18}N_2O_3S_2Na^+$: 361.06511; Found: 361.06456.

**N-(Amino(4-fluorophenyl)(oxo)-λ⁶-sulfaneylidene)-4-methylbenzenesulfonamide (12o)**

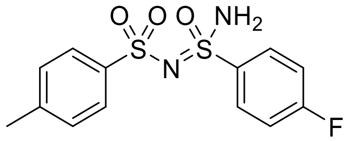


The title compound **12o** was synthesized according to the general procedure (GP-4), and was obtained after silica column chromatography (DCM : Methanol/$NH_3$ – 40:1 – 20:1 – 9:1) as a yellow solid (87%, 57 mg).

**$^1$H NMR** (600 MHz, DMSO-*d*$_6$): δ = 8.15 (s, 2H), 7.86 – 7.69 (m, 2H), 7.52 (d, *J* = 8.2 Hz, 2H), 7.38 (t, *J* = 8.8 Hz, 2H), 7.24 (d, *J* = 8.1 Hz, 2H), 2.33 (s, 3H) ppm.

**$^{13}$C NMR** (151 MHz, DMSO-*d*$_6$): δ = 164.7 (d, *J* = 252.3 Hz), 142.4, 141.2, 138.7, 130.1 (d, *J* = 10.3 Hz), 129.5, 126.4, 116.5 (d, *J* = 23.4 Hz), 21.3 ppm.

**$^{19}$F NMR** (564 MHz, DMSO-*d*$_6$): δ = -106.37 (ddd, *J* = 13.9, 8.8, 5.0 Hz) ppm.

**HRMS** (ESI): *m/z*: [M + Na]$^+$ Calcd. for $C_{13}H_{13}N_2O_3FS_2Na^+$: 351.02438; Found: 351.02396.

**N-(Amino(4-chlorophenyl)(oxo)-λ⁶-sulfaneylidene)-4-methylbenzenesulfonamide (12p)**

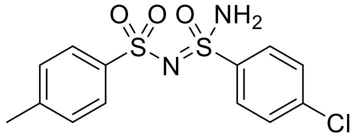


The title compound **12p** was synthesized according to the general procedure (GP-4), and was obtained after silica column chromatography (DCM : Methanol/$NH_3$ – 40:1 – 20:1 – 9:1) as a yellow solid (90%, 62 mg).

**$^1$H NMR** (600 MHz, Chloroform-*d*): δ = 7.90 (d, *J* = 8.8 Hz, 2H), 7.81 (d, *J* = 8.3 Hz, 2H), 7.47 (d, *J* = 8.8 Hz, 2H), 7.28 – 7.26 (m, 2H), 5.76 (s, 2H), 2.41 (s, 3H) ppm.

**$^{13}$C NMR** (151 MHz, Chloroform-*d*) δ 143.3, 140.5, 139.6, 138.7, 129.4, 129.3, 128.5, 128.5, 126.6, 21.5 ppm.

**HRMS** (ESI): *m/z*: [M + Na]$^+$ Calcd. for $C_{13}H_{14}N_2O_3ClS_2Na^+$: 345.01289; Found: 345.01303.

**N-(Amino(4-bromophenyl)(oxo)-λ⁶-sulfaneylidene)-4-methylbenzenesulfonamide (12q)**

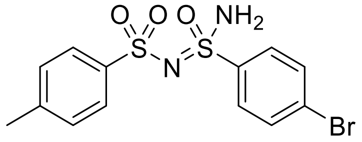


The title compound **12q** was synthesized according to the general procedure (GP-4), and was obtained after silica column chromatography (DCM : Methanol/$NH_3$ – 40:1 – 20:1 – 9:1) as a yellow solid (82%, 63 mg).

**$^1$H NMR** (600 MHz, Chloroform-*d*): δ = 7.80 (dd, *J* = 11.0, 8.5 Hz, 4H), 7.62 (d, *J* = 8.7 Hz, 2H), 7.26 – 7.23 (m, 3H), 5.81 (s, 2H), 2.41 (s, 3H) ppm.

**$^{13}$C NMR** (151 MHz, Chloroform-*d*): δ = 143.3, 139.6, 139.3, 132.4, 129.3, 129.3, 129.0, 128.5, 126.6, 116.3, 21.5 ppm.

**HRMS** (ESI): *m/z*: $[M + Na]^+$ Calcd. for $C_{13}H_{14}N_2O_3BrS_2Na^+$: 388.96237; Found: 388.96241.

**N-(Amino(3-methoxyphenyl)(oxo)-λ⁶-sulfaneylidene)-4-methylbenzenesulfonamide (12r)**

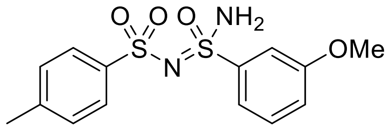


The title compound **12r** was synthesized according to the general procedure (GP-4), and was obtained after silica column chromatography (DCM : Methanol/$NH_3$ – 40:1 – 20:1 – 9:1) as a yellow solid (66%, 45 mg).

**$^1$H NMR** (600 MHz, DMSO-$d_6$): δ = 8.05 (s, 2H), 7.49 (d, *J* = 8.3 Hz, 2H), 7.45 (t, *J* = 8.0 Hz, 1H), 7.35 (d, *J* = 8.5 Hz, 1H), 7.22 (d, *J* = 8.0 Hz, 2H), 7.20 – 7.14 (m, 2H), 3.74 (s, 3H), 2.32 (s, 3H) ppm.

**$^{13}$C NMR** (151 MHz, DMSO-$d_6$): δ = 159.5, 143.6, 142.3, 141.2, 129.4, 126.4, 119.1, 119.0, 111.9, 56.0, 21.3 ppm.

**HRMS** (ESI): *m/z*: $[M + H]^+$ Calcd. for $C_{14}H_{17}N_2O_4S_2^+$: 341.06243; Found: 341.06141.

**N-(Amino(2-methoxyphenyl)(oxo)-λ⁶-sulfaneylidene)-4-methylbenzenesulfonamide (12s)**

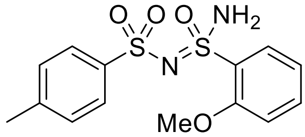


The title compound **12s** was synthesized according to the general procedure (GP-4), and was obtained after silica column chromatography (DCM : Methanol/$NH_3$ – 40:1 – 20:1 – 9:1) as a yellow solid (46%, 32 mg).

**$^1$H NMR** (600 MHz, Chloroform-*d*): δ = 7.95 (dt, *J* = 8.0, 1.4 Hz, 1H), 7.79 (d, *J* = 7.9 Hz, 2H), 7.57 (td, *J* = 8.0, 7.4, 1.5 Hz, 1H), 7.23 (d, *J* = 8.0 Hz, 2H), 7.08 (t, *J* = 7.7 Hz, 1H), 6.99 (d, *J* = 8.4 Hz, 1H), 5.69 (s, 2H), 3.95 (s, 3H), 2.39 (s, 3H) ppm.

**$^{13}$C NMR** (151 MHz, Chloroform-*d*): δ = 156.0, 142.7, 140.3, 135.5, 129.1, 129.0, 128.4, 126.6, 120.7, 112.5, 56.5, 21.5 ppm.

**HRMS** (ESI): *m/z*: $[M + Na]^+$ Calcd. for $C_{14}H_{16}N_2O_4S_2Na^+$: 363.04437; Found: 363.04397.

***N*-(Amino(naphthalen-2-yl)(oxo)-λ⁶-sulfaneylidene)-4-methylbenzenesulfonamide (12t)**

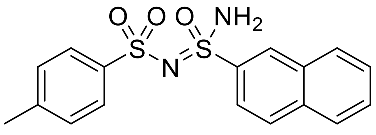

The title compound **12t** was synthesized according to the general procedure (GP-4), and was obtained after silica column chromatography (DCM : Methanol/$NH_3$ – 40:1 – 20:1 – 9:1) as a yellow solid (82%, 59 mg).

**$^{1}$H NMR** (600 MHz, Chloroform-*d*): δ = 8.49 (s, 1H), 7.93 – 7.85 (m, 4H), 7.79 (d, *J* = 8.3 Hz, 2H), 7.68 – 7.64 (m, 1H), 7.62 – 7.58 (m, 1H), 7.15 (d, *J* = 8.0 Hz, 2H), 5.93 (s, 2H), 2.32 (s, 3H) ppm.

**$^{13}$C NMR** (151 MHz, Chloroform-*d*): δ = 143.0, 139.7, 136.6, 135.1, 131.7, 129.6, 129.5, 129.4, 129.2, 128.7, 127.8, 127.7, 126.6, 121.8, 21.4 ppm.

**HRMS** (ESI): *m/z*: $[M + Na]^+$ Calcd. for $C_{17}H_{16}N_2O_3S_2Na^+$: 383.04946; Found: 383.04892.

***N*-(Amino(benzyl)(oxo)- λ⁶-sulfaneylidene)-4-methylbenzenesulfonamide (12u)**

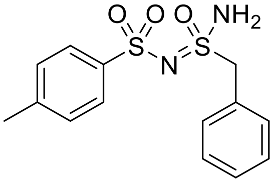

The title compound **12u** was synthesized according to the general procedure (GP-4), and was obtained after silica column chromatography (DCM : Methanol/$NH_3$ – 40:1 – 20:1 – 9:1) as a yellow solid (44%, 28 mg).

**$^{1}$H NMR** (600 MHz, DMSO-*d*$_6$): δ = 7.64 (d, *J* = 8.2 Hz, 2H), 7.56 (s, 2H), 7.44 – 7.35 (m, 5H), 7.29 (d, *J* = 8.0 Hz, 2H), 4.65 – 4.58 (m, 2H), 2.34 (s, 3H) ppm.

**$^{13}$C NMR** (151 MHz, DMSO-*d*$_6$): δ = 142.2, 142.1, 131.9, 129.5, 129.3, 129.0, 128.8, 126.4, 62.3, 21.3 ppm.

**HRMS** (ESI): *m/z*: $[M + Na]^+$ Calcd. for $C_{14}H_{16}N_2O_3S_2Na^+$: 347.04945; Found: 347.04926.

***N*-(Amino(oxo)(phenethyl)-λ⁶-sulfaneylidene)-4-methylbenzenesulfonamide (12v)**

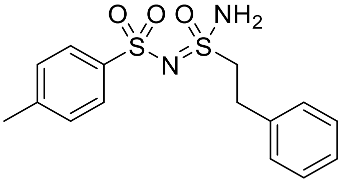

The title compound **12v** was synthesized according to the general procedure (GP-4), and was obtained after silica column chromatography (DCM : Methanol/$NH_3$ – 40:1 – 20:1 – 9:1) as a yellow solid (92%, 62 mg).

**$^{1}$H NMR** (600 MHz, Chloroform-*d*): δ = 7.85 (d, *J* = 8.1 Hz, 2H), 7.31 – 7.20 (m, 5H), 7.16 (d, *J* = 6.9 Hz, 2H), 5.61 (s, 2H), 3.65 – 3.52 (m, 2H), 3.21 – 3.08 (m, 2H), 2.40 (s, 3H) ppm.

**$^{13}$C NMR** (151 MHz, Chloroform-*d*): δ = 143.2, 140.0, 136.6, 129.4, 128.9, 128.5, 127.1, 126.5, 58.8, 29.3, 21.5.

**HRMS** (ESI): *m/z*: $[M + Na]^+$ Calcd. for $C_{15}H_{18}N_2O_3S_2Na^+$: 361.06511; Found: 361.06456.

**(*R*)-*N*-(Amino(tert-butyl)(oxo)-λ⁶-sulfaneylidene)-4-methylbenzenesulfonamide (12w)**

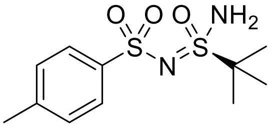

The title compound **12w** was synthesized according to the general procedure (GP-4), and was obtained after silica column chromatography (DCM : Methanol/$NH_3$ – 40:1 – 20:1 – 9:1) as a yellow solid (93%, 54 mg).

**HPLC** (ADH, *n*-hexane/2-propanol = 90/10, flow rate = 0.75 mL/min, λ = 220 nm, temp = rt) $t_R$ = 23.1 min (major), 27.9 min (minor), *er* = 99.98:0.02.

**$^1$H NMR** (600 MHz, Chloroform-*d*): δ = 7.84 (d, *J* = 8.3 Hz, 2H), 7.26 (d, *J* = 8.5 Hz, 2H), 5.46 (s, 2H), 2.39 (s, 3H), 1.43 (s, 9H) ppm.

**$^{13}$C NMR** (151 MHz, Chloroform-*d*): δ = 142.8, 140.4, 129.3, 126.4, 62.8, 23.1, 21.5 ppm.

**HRMS** (ESI): *m/z*: $[M + Na]^+$ Calcd. for $C_{11}H_{18}N_2O_3S_2Na^+$: 313.06510; Found: 313.06476.

***N*-((Isopropylamino)(oxo)(p-tolyl)-λ⁶-sulfaneylidene)-4-methylbenzenesulfonamide (12x)**

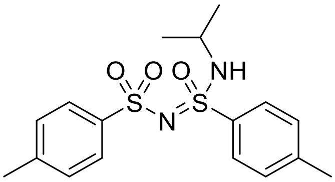

The title compound **12x** was synthesized according to the general procedure (GP-4), and was obtained after silica column chromatography (DCM : Methanol/$NH_3$ – 40:1 – 20:1 – 9:1) as a yellow solid (68%, 50 mg).

**$^1$H NMR** (600 MHz, Chloroform-*d*): δ = 7.84 – 7.77 (m, 3H), 7.24 (dd, *J* = 23.1, 7.8 Hz, 4H), 5.95 (d, *J* = 6.9 Hz, 1H), 3.48 – 3.38 (m, 1H), 2.40 (s, 3H), 2.38 (s, 3H), 1.20 (d, *J* = 6.5 Hz, 3H), 0.98 (d, *J* = 6.6 Hz, 3H) ppm.

**$^{13}$C NMR** (151 MHz, Chloroform-*d*): δ = 144.5, 142.7, 140.4, 136.1, 129.7, 129.1, 127.8, 126.7, 46.2, 24.0, 22.9, 21.5, 21.5 ppm.

**HRMS** (ESI): *m/z*: $[M + Na]^+$ Calcd. for $C_{17}H_{22}N_2O_3S_2Na^+$: 389.09641; Found: 389.09604.

**$N$-((cyclohexylamino)(oxo)($p$-tolyl)-$\lambda^6$-sulfaneylidene)-4-methylbenzenesulfonamide (12y)**

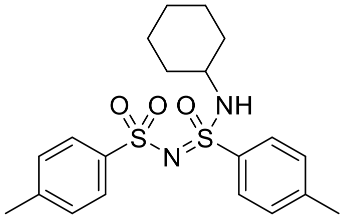

The title compound **12y** was synthesized according to the general procedure (GP-4), and was obtained after silica column chromatography (DCM : Methanol/$NH_3$ – 40:1 – 20:1 – 9:1) as a yellow solid (57%, 46 mg).

**$^1$H NMR** (600 MHz, Chloroform-*d*): δ = 7.87 – 7.74 (m, 4H), 7.27 (d, *J* = 8.2 Hz, 2H), 7.24 (d, *J* = 8.0 Hz, 2H), 5.87 (d, *J* = 7.7 Hz, 1H), 3.13 (dtt, *J* = 10.1, 7.0, 3.8 Hz, 1H), 2.42 (s, 3H), 2.39 (s, 3H), 1.96 – 1.89 (m, 1H), 1.73 – 1.65 (m, 1H), 1.56 (ddt, *J* = 18.5, 10.8, 5.0 Hz, 2H), 1.50 (t, *J* = 7.5 Hz, 1H), 1.33 (ddd, *J* = 12.8, 10.0, 3.5 Hz, 1H), 1.29 – 1.19 (m, 2H), 1.15 – 1.10 (m, 2H) ppm.

**$^{13}$C NMR** (151 MHz, Chloroform-*d*): δ = 144.5, 142.7, 140.3, 136.4, 129.7, 129.1, 127.7, 126.7, 52.6, 34.1, 33.0, 25.0, 24.5, 21.58, 21.53 ppm.

**HRMS** (ESI): *m/z*: $[M + Na]^+$ Calcd. for $C_{20}H_{26}N_2O_3S_2Na^+$: 429.12771; Found: 429.12712.

**$N$-((Benzylamino)(oxo)(p-tolyl)-$\lambda^6$-sulfaneylidene)-4-methylbenzenesulfonamide(12z)**

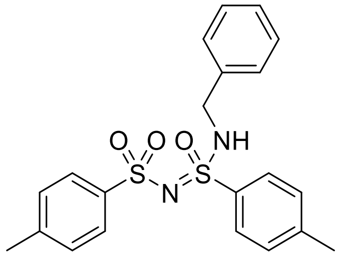

The title compound **12z** was synthesized according to the general procedure (GP-4), and was obtained after silica column chromatography (DCM : Methanol/$NH_3$ – 40:1 – 20:1 – 9:1) as a yellow solid (31%, 26 mg).

**$^1$H NMR** (600 MHz, Chloroform-*d*): δ = 7.82 (dd, *J* = 8.4, 6.4 Hz, 4H), 7.31 – 7.20 (m, 7H), 7.20 – 7.12 (m, 2H), 6.03 (s, 1H), 4.18 (dd, *J* = 13.8, 4.1 Hz, 1H), 4.07 (dd, *J* = 13.5, 3.9 Hz, 1H), 2.42 (s, 3H), 2.40 (s, 3H) ppm.

**$^{13}$C NMR** (151 MHz, Chloroform-*d*): δ = 144.9, 142.8, 140.3, 135.2, 134.8, 129.8, 129.2, 128.7, 128.0, 127.8, 126.7, 46.7, 21.5, 21.5 ppm.

**HRMS** (ESI): *m/z*: $[M + Na]^+$ Calcd. for $C_{21}H_{22}N_2O_3S_2Na^+$: 437.09641; Found: 437.09663.

**4-Methyl-*N*-(oxo(phenethylamino)(*p*-tolyl)-λ$^6$-sulfaneylidene) benzenesulfonamide (12aa)**

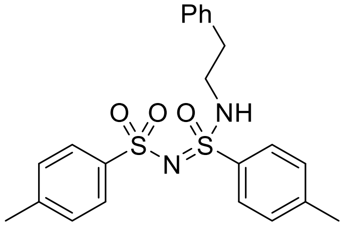

The title compound **12aa** was synthesized according to the general procedure (GP-4), and was obtained after silica column chromatography (DCM : Methanol/$NH_3$ – 40:1 – 20:1 – 9:1) as a yellow solid (58%, 50 mg).
$^1$**H NMR** (600 MHz, Chloroform-*d*): δ = 7.80 (d, *J* = 8.3 Hz, 2H), 7.71 (d, *J* = 8.4 Hz, 2H), 7.29 – 7.20 (m, 7H), 7.08 (d, *J* = 6.8 Hz, 2H), 5.67 – 5.57 (m, 1H), 3.26 (dq, *J* = 13.1, 6.6 Hz, 1H), 3.16 (dq, *J* = 12.8, 6.7 Hz, 1H), 2.77 (td, *J* = 7.0, 3.3 Hz, 2H), 2.41 (s, 3H), 2.39 (s, 3H) ppm.
$^{13}$**C NMR** (151 MHz, Chloroform-*d*): δ = 144.8, 142.8, 140.3, 137.2, 134.8, 129.8, 129.2, 128.78, 128.72, 127.7, 126.9, 126.6, 43.8, 35.4, 21.58, 21.53.
**HRMS** (ESI): *m/z*: $[M + Na]^+$ Calcd. for $C_{22}H_{24}N_2O_3S_2Na^+$: 451.11206; Found: 451.11149.

**4-Methyl-*N*-(oxo(*p*-tolyl)((2,2,2-trifluoroethyl)amino)-λ$^6$-sulfaneylidene)benzenesulfonamide (12z)**

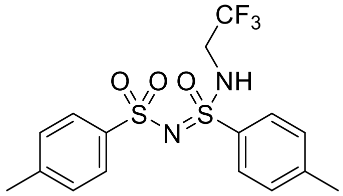

The title compound **12z** was synthesized according to the general procedure (GP-4), and was obtained after silica column chromatography (DCM : Methanol/$NH_3$ – 40:1 – 20:1 – 9:1) as a yellow solid (63%, 51 mg).
$^1$**H NMR** (600 MHz, Chloroform-*d*): δ = 7.84 (t, *J* = 8.3 Hz, 4H), 7.31 (d, *J* = 8.1 Hz, 2H), 7.27 (d, *J* = 8.1 Hz, 3H), 6.39 (s, 1H), 3.78 – 3.62 (m, 2H), 2.43 (s, 3H), 2.41 (s, 3H) ppm.
$^{13}$**C NMR** (151 MHz, Chloroform-*d*): δ 145.4, 143.3, 139.8, 134.9, 129.9, 129.3, 127.6, 126.7, 123.3 (q, *J* = 278.4 Hz), 43.8 (q, *J* = 35.6 Hz), 21.6, 21.5 ppm.
$^{19}$**F NMR** (565 MHz, Chloroform-*d*): δ = -72.04 (t, *J* = 8.6 Hz) ppm.
**HRMS** (ESI): *m/z*: $[M + H]^+$ Calcd. for $C_{16}H_{18}N_2O_3F_3S_2^+$: 407.07054; Found: 407.06920.

**N-(((cyclopropylmethyl)amino)(oxo)(p-tolyl)-λ⁶-sulfaneylidene)-4-methylbenzenesulfonamide (12ac)**

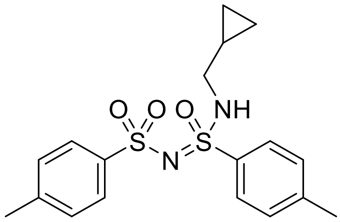


The title compound **12ac** was synthesized according to the general procedure (GP-4), and was obtained after silica column chromatography (DCM : Methanol/$NH_3$ – 40:1 – 20:1 – 9:1) as a yellow solid (69%, 52 mg).

**$^1$H NMR** (600 MHz, Chloroform-*d*): δ = 7.82 (dd, *J* = 14.1, 8.4 Hz, 4H), 7.29 (d, *J* = 7.8 Hz, 2H), 7.24 (d, *J* = 7.8 Hz, 2H), 5.82 (t, *J* = 5.8 Hz, 1H), 2.88 (ddd, *J* = 13.1, 7.0, 6.1 Hz, 1H), 2.75 (ddd, *J* = 12.9, 7.4, 5.5 Hz, 1H), 2.42 (s, 3H), 2.39 (s, 3H), 0.89 (dddd, *J* = 15.2, 10.4, 5.2, 2.6 Hz, 1H), 0.52 – 0.42 (m, 2H), 0.12 (ddddd, *J* = 13.0, 11.1, 9.4, 4.7, 3.5 Hz, 2H) ppm.

**$^{13}$C NMR** (151 MHz, Chloroform-*d*): δ - 144.7, 142.8, 140.4, 135.1, 129.7, 129.1, 127.8, 126.7, 47.9, 21.58, 21.53, 10.4, 3.8, 3.6 ppm.

**HRMS** (ESI): *m/z*: $[M + Na]^+$ Calcd. for $C_{18}H_{22}N_2O_3S_2Na^+$: 401.09641; Found: 401.09574.

**N-(((2-methoxyethyl)amino)(oxo)(p-tolyl)-λ⁶-sulfaneylidene)-4-methylbenzenesulfonamide (12ad)**

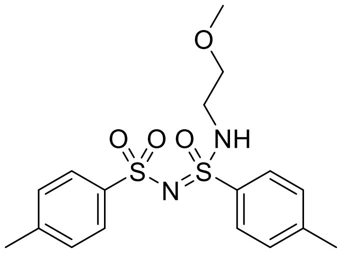


The title compound **12ad** was synthesized according to the general procedure (GP-4), and was obtained after silica column chromatography (DCM : Methanol/$NH_3$ – 40:1 – 20:1 – 9:1) as a yellow solid (39%, 30 mg).

**$^1$H NMR** (600 MHz, Chloroform-*d*): δ = 7.83 (d, *J* = 8.3 Hz, 2H), 7.80 (d, *J* = 8.4 Hz, 2H), 7.30 (d, *J* = 8.3 Hz, 2H), 7.24 (d, *J* = 8.1 Hz, 2H), 5.78 (s, 1H), 3.43 – 3.36 (m, 2H), 3.29 (s, 3H), 3.18 – 3.05 (m, 2H), 2.42 (s, 3H), 2.39 (s, 3H) ppm.

**$^{13}$C NMR** (151 MHz, Chloroform-*d*): δ = 144.8, 142.8, 140.4, 135.0, 129.8, 129.1, 127.7, 126.7, 69.9, 58.8, 42.3, 21.5, 21.5 ppm.

**HRMS** (ESI): *m/z*: $[M + Na]^+$ Calcd. for $C_{17}H_{22}N_2O_4S_2Na^+$: 405.09132; Found: 405.09076.

***N*-(Amino(4-(5-methyl-3-phenylisoxazol-4-yl)phenyl)(oxo)-λ$^6$-sulfaneylidene)-4-methylbenzenesulfonamide (12ae)**

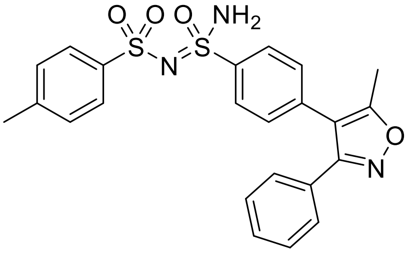


The title compound **12ae** was synthesized according to the general procedure (GP-4), and was obtained after silica column chromatography (DCM : Methanol/$NH_3$ – 40:1 – 20:1 – 9:1) as a yellow solid (64%, 60 mg).

**$^1$H NMR** (600 MHz, DMSO-$d_6$): δ = 7.91 (s, 2H), 7.76 (d, *J* = 8.5 Hz, 2H), 7.54 (d, *J* = 8.3 Hz, 2H), 7.45 (t, *J* = 7.3 Hz, 1H), 7.40 (dd, *J* = 8.2, 6.7 Hz, 2H), 7.37 – 7.31 (m, 4H), 7.22 (d, *J* = 7.9 Hz, 2H), 2.46 (s, 3H), 2.30 (s, 3H) ppm.

**$^{13}$C NMR** (151 MHz DMSO-$d_6$): δ = 168.1, 161.1, 142.5, 142.1, 141.7, 130.2, 129.4, 129.2, 128.7, 128.6, 127.3, 126.4, 122.0, 114.5, 21.3, 11.8, 2.1 ppm.

**HRMS** (ESI): *m/z*: [M + Na]$^+$ Calcd. for $C_{23}H_{21}N_3O_4S_2Na^+$: 490.08657; Found: 490.08605.

**N-(Amino(oxo)(phenyl)-λ⁶-sulfaneylidene)-4-(5-methyl-3-phenylisoxazol-4-yl)benzenesulfonamide (12af)**

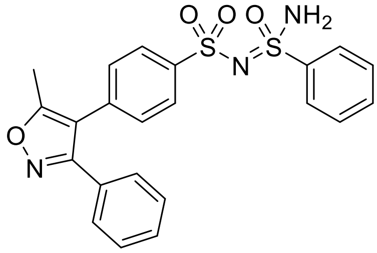


The title compound **12af** was synthesized according to the general procedure (GP-4), and was obtained after silica column chromatography (DCM : Methanol/$NH_3$ – 40:1 – 20:1 – 9:1) as a yellow solid (62%, 56 mg).
$^1$**H NMR** (600 MHz, DMSO-*d*$_6$): δ = 8.18 (s, 2H), 7.84 – 7.74 (m, 2H), 7.65 – 7.59 (m, 3H), 7.53 (t, *J* = 7.9 Hz, 2H), 7.47 – 7.43 (m, 1H), 7.41 (t, *J* = 7.3 Hz, 2H), 7.32 (d, *J* = 6.9 Hz, 2H), 7.27 (d, *J* = 8.4 Hz, 2H), 2.45 (s, 3H) ppm.
$^{13}$**C NMR** (151 MHz, DMSO-*d*$_6$): δ = 168.0, 161.0, 143.1, 142.3, 133.6, 133.4, 130.2, 130.1, 129.4, 129.2, 128.8, 128.6, 127.0, 126.8, 114.6, 11.8 ppm.
**HRMS** (ESI): *m/z*: [M + Na]$^+$ Calcd. for $C_{22}H_{19}N_3O_4S_2Na^+$: 476.07092; Found: 476.07094.

**N-(Amino(oxo)(phenyl)-λ⁶-sulfaneylidene)-4-(5-(*p*-tolyl)-3-(trifluoromethyl)-1*H*-pyrazol-1-yl)benzenesulfonamide (12ag)**

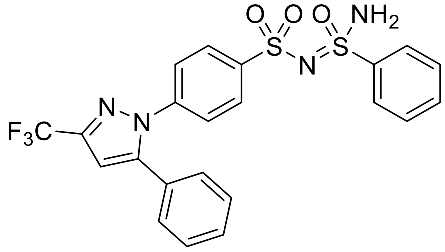


The title compound **12ag** was synthesized according to the general procedure (GP-4), and was obtained after silica column chromatography (DCM : Methanol/$NH_3$ – 40:1 – 20:1 – 9:1) as a yellow solid (52%, 54 mg).
$^1$**H NMR** (600 MHz, DMSO-*d*$_6$): δ = 8.23 (s, 2H), 7.81 (d, *J* = 8.5 Hz, 2H), 7.71 (d, *J* = 8.6 Hz, 2H), 7.64 (t, *J* = 7.4 Hz, 1H), 7.59 – 7.53 (m, 2H), 7.43 (d, *J* = 8.6 Hz, 2H), 7.21 (d, *J* = 8.1 Hz, 2H), 7.19 – 7.14 (m, 3H), 2.30 (s, 3H) ppm.
$^{13}$**C NMR** (151 MHz, DMSO-*d*$_6$): δ = 145.6, 144.0, 142.6 (q, *J* = 38.0 Hz), 142.3, 141.5, 139.5, 133.5, 129.8, 129.5, 129.1, 127.6, 127.0, 126.2, 125.7, 121.7 (q, *J* = 268.7 Hz), 106.5, 21.2 ppm.
$^{19}$**F NMR** (564 MHz, DMSO-*d*$_6$): δ = -60.90 ppm.
**HRMS** (ESI): *m/z*: [M + Na]$^+$ Calcd. for $C_{23}H_{20}N_4O_3F_3S_2Na^+$: 521.09234; Found: 521.09251.

**4-Methyl-*N*-(((methyl(phenyl)-λ[4]-sulfaneylidene)amino)(oxo)(phenyl)-λ[6]-sulfaneylidene)benzenesulfonamide (14)**

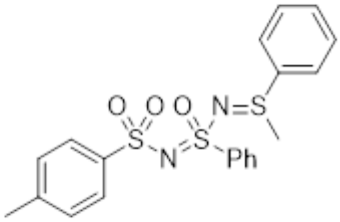


The title compound **14** was synthesized according to the general procedure (GP-5), and was obtained after silica column chromatography (DCM : Methanol – 40:1 – 20:1) as a yellow solid (96%, 42 mg).

$^{1}$**H NMR** (600 MHz, Chloroform-*d*): δ = 7.86 (dd, *J* = 8.5, 1.2 Hz, 2H), 7.82 (d, *J* = 8.2 Hz, 2H), 7.75 (dd, *J* = 8.5, 1.3 Hz, 2H), 7.72 – 7.69 (m, 2H), 7.68 (d, *J* = 8.2 Hz, 2H), 7.64 – 7.61 (m, 2H), 7.57 – 7.47 (m, 4H), 7.47 – 7.40 (m, 3H), 7.38 – 7.32 (m, 3H), 7.28 – 7.24 (m, 2H), 7.17 (d, *J* = 7.8 Hz, 2H), 7.08 (d, *J* = 7.8 Hz, 2H), 3.04 (s, 3H), 3.01 (s, 3H), 2.35 (s, 3H), 2.31 (s, 3H) ppm.

$^{13}$**C NMR** (151 MHz, Chloroform-*d*): δ = 142.6, 142.2, 142.0, 141.6, 141.2, 141.0, 135.5, 134.6, 132.8, 132.7, 132.2, 132.0, 130.12, 130.11, 128.9, 128.7, 128.6, 126.8, 126.7, 126.6, 126.2, 126.1, 126.0, 39.2, 38.5, 21.48, 21.42 ppm.

**HRMS** (ESI): *m/z*: [M + H]$^+$ Calcd. for $C_{20}H_{21}N_2O_3S_3^+$: 433.07088; Found: 433.07088.

***N*-(((Dimethyl(oxo)-λ[6]-sulfaneylidene)amino)(oxo)(phenyl)-λ[6]-sulfaneylidene)-4-methylbenzenesulfonamide(16)**

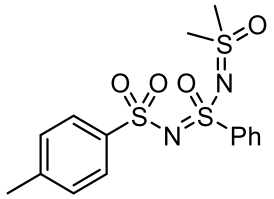


The title compound **16** was synthesized according to the general procedure (GP-5), and was obtained after silica column chromatography (DCM : Methanol – 40:1 – 20:1) as a yellow solid (67%, 26 mg).

$^{1}$**H NMR** (600 MHz, Chloroform-*d*): δ = 7.94 (d, *J* = 8.7 Hz, 2H), 7.80 (d, *J* = 8.3 Hz, 2H), 7.54 (t, *J* = 7.4 Hz, 1H), 7.45 (t, *J* = 7.9 Hz, 2H), 7.21 (d, *J* = 8.0 Hz, 2H), 3.60 (s, 3H), 3.35 (s, 3H), 2.37 (s, 3H) ppm.

$^{13}$**C NMR** (151 MHz, Chloroform-*d*): δ = 142.3, 142.1, 140.8, 132.9, 129.0, 128.8, 126.55, 126.54, 44.47, 44.40, 21.4 ppm.

**HRMS** (ESI): *m/z*: [M + Na]$^+$ Calcd. for $C_{15}H_{18}N_2O_4S_2Na^+$: 409.03209; Found: 409.03183.

**4*N*-((Allylamino)(oxo)(phenyl)-λ$^6$-sulfaneylidene)-4-methylbenzenesulfonamide (18)**

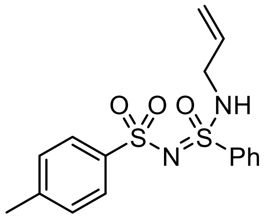

The title compound **18** was synthesized according to the general procedure (GP-5), and was obtained after silica column chromatography (DCM : Methanol – 40:1 – 20:1) as a yellow solid (98%, 35 mg).
$^1$**H NMR** (600 MHz, Chloroform-*d*): δ = 7.94 (dd, *J* = 8.5, 1.3 Hz, 2H), 7.83 (d, *J* = 8.3 Hz, 2H), 7.60 (t, *J* = 7.5 Hz, 1H), 7.52 – 7.47 (m, 2H), 7.24 (d, *J* = 8.0 Hz, 2H), 6.00 (s, 1H), 5.68 (ddt, *J* = 17.0, 10.2, 5.9 Hz, 1H), 5.22 – 5.15 (m, 1H), 5.10 (dq, *J* = 10.2, 1.2 Hz, 1H), 3.60 (dddt, *J* = 51.9, 15.0, 6.1, 1.5 Hz, 2H), 2.39 (s, 3H) ppm.
$^{13}$**C NMR** (151 MHz, Chloroform-*d*): δ = 142.9, 140.2, 138.2, 133.6, 132.0, 129.26, 129.22, 127.7, 126.7, 118.5, 45.1, 21.5 ppm.
**HRMS** (ESI): *m/z*: [M + H]$^+$ Calcd. for $C_{16}H_{19}N_2O_3S_2^+$: 351.08316; Found: 351.08265.

**4-Methyl-*N*-(oxo(phenyl)(2-phenylaziridin-1-yl)- λ$^6$-sulfaneylidene) benzenesulfonamide (19a)**

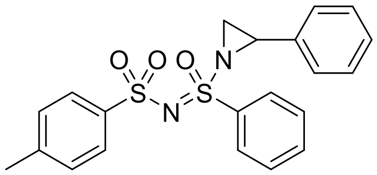

The title compound **19a** was synthesized according to the general procedure (GP-6), and was obtained after silica column chromatography (DCM : methanol – 40:1 – 20:1) as a yellow solid (78%, 32 mg).
$^1$**H NMR** (600 MHz, Chloroform-*d*): δ = 8.01 – 7.95 (m, 4H), 7.82 (d, *J* = 8.3 Hz, 2H), 7.78 (d, *J* = 8.3 Hz, 2H), 7.65 – 7.60 (m, 2H), 7.50 (dt, *J* = 8.5, 7.1 Hz, 4H), 7.30 – 7.25 (m, 6H), 7.22 (d, *J* = 8.0 Hz, 2H), 7.19 (dt, *J* = 7.0, 2.4 Hz, 4H), 7.14 – 7.11 (m, 2H), 4.02 (dd, *J* = 7.3, 4.8 Hz, 1H), 3.87 (dd, *J* = 7.3, 4.8 Hz, 1H), 3.27 (d, *J* = 7.5 Hz, 1H), 3.14 (d, *J* = 7.3 Hz, 1H), 2.62 (dd, *J* = 4.8, 0.8 Hz, 1H), 2.47 (d, *J* = 4.9 Hz, 1H), 2.39 (s, 3H), 2.37 (s, 3H) ppm.
$^{13}$**C NMR** (151 MHz, Chloroform-*d*): δ = 142.8, 142.7, 140.3, 137.6, 137.5, 137.4, 134.2, 133.8, 133.7, 129.29, 129.26, 129.21, 129.1, 128.6, 128.6, 127.8, 126.7, 126.6, 126.5, 43.7, 42.3, 37.9, 36.2, 21.5 ppm.
**HRMS** (ESI): *m/z*: [M + K]$^+$ Calcd. for $C_{21}H_{20}N_2O_3S_2K^+$: 451.05469; Found: 451.05382.

**4-(5-Methyl-3-phenylisoxazol-4-yl)-*N*-(oxo(phenyl)(2-phenylaziridin-1-yl)- λ⁶-sulfaneylidene)benzenesulfonamide (19b)**

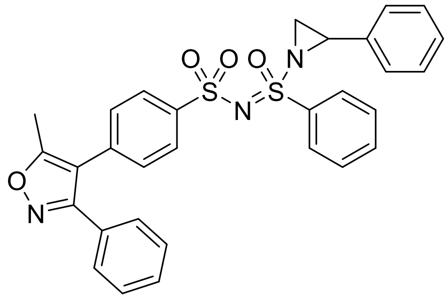


The title compound **19b** was synthesized according to the general procedure (GP-6), and was obtained after silica column chromatography (DCM : methanol – 40:1 – 20:1) as a yellow solid (51%, 28 mg).

$^{1}$**H NMR** (600 MHz, Chloroform-*d*): δ = 8.02 (dt, *J* = 8.6, 1.5 Hz, 2H), 7.94 (d, *J* = 8.5 Hz, 2H), 7.89 (d, *J* = 8.4 Hz, 2H), 7.68 – 7.63 (m, 2H), 7.53 (q, *J* = 8.1 Hz, 4H), 7.37 (ddd, *J* = 7.2, 3.0, 1.6 Hz, 5H), 7.35 – 7.31 (m, 5H), 7.31 – 7.26 (m, 7H), 7.25 (d, *J* = 8.5 Hz, 3H), 7.23 – 7.20 (m, 3H), 7.15 – 7.12 (m, 3H), 4.08 (dd, *J* = 7.4, 4.9 Hz, 1H), 3.89 (dd, *J* = 7.3, 4.8 Hz, 1H), 3.32 (dd, *J* = 7.4, 0.9 Hz, 1H), 3.16 (d, *J* = 7.3 Hz, 1H), 2.68 (dd, *J* = 4.8, 0.9 Hz, 1H), 2.52 (d, *J* = 4.9 Hz, 1H), 2.47 (s, 6H) ppm.

$^{13}$**C NMR** (151 MHz, Chloroform-*d*): δ = 167.1, 161.0, 142.3, 134.4, 133.6, 129.85, 129.82, 129.6, 129.38, 129.35, 128.8, 128.7, 128.6, 128.4, 127.8, 127.1, 127.0, 126.7, 126.5, 114.6, 43.9, 42.5, 38.0, 36.4, 11.7 ppm.

**HRMS** (ESI): *m/z*: [M + Na]$^+$ Calcd. for $C_{30}H_{25}N_3O_4S_2Na^+$: 578.11787; Found: 578.11820.

**Diethyl(3a*R*,6a*S*)-2-(*N*-tosylphenylsulfonimidoyl)hexahydrocyclopenta[*c*]pyrrole-5,5(1*H*)-dicarboxylate (20a)**

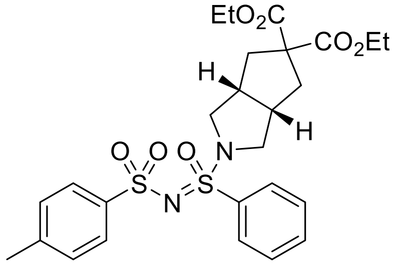


The title compound **20a** was synthesized according to the general procedure (GP-7), and was obtained after silica column chromatography (*n*-Pentane : $Et_2O$ 2:1 – 1:1 – 1:2) as a yellow solid (67%, 37 mg).

$^{1}$**H NMR** (600 MHz, Chloroform-*d*): δ = 7.92 – 7.85 (m, 4H), 7.63 (t, *J* = 7.5 Hz, 1H), 7.56 – 7.50 (m, 2H), 7.25 (d, *J* = 7.9 Hz, 2H), 4.18 – 4.11 (m, 4H), 3.33 (dd, *J* = 9.8, 2.8 Hz, 1H), 3.15 (td, *J* = 9.9, 7.1 Hz, 2H), 3.09 (dd, *J* = 9.9, 2.9 Hz, 1H), 2.82 – 2.68 (m, 2H), 2.56 – 2.47 (m, 2H), 2.39 (s, 3H), 1.98 (dd, *J* = 13.4, 8.0 Hz, 1H), 1.89 (dd, *J* = 13.4, 8.0 Hz, 1H), 1.22 (td, *J* = 7.1, 2.5 Hz, 6H) ppm.

$^{13}$**C NMR** (151 MHz, Chloroform-*d*): δ = 171.5, 170.8, 142.7, 140.7, 135.1, 133.7, 129.1, 127.8, 126.8, 62.1, 61.5, 53.7, 53.5, 41.9, 41.7, 39.3, 39.2, 21.5, 14.0 ppm.

**HRMS** (ESI): *m/z*: [M + Na]$^+$ Calcd. for $C_{26}H_{32}N_2O_7S_2Na^+$: 571.15431; Found: 571.15431.

**N-(((3aR,6aS)-Hexahydrocyclopenta[c]pyrrol-2(1H)-yl)(oxo)(phenyl)-λ⁶-sulfaneylidene)-4-methylbenzenesulfonamide (20b)**

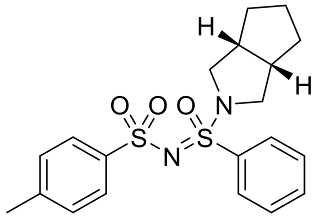

The title compound **20b** was synthesized according to the general procedure (GP-7), and was obtained after silica column chromatography (*n*-Pentane : $Et_2O$ 2:1 – 1:1 – 1:2) as a yellow solid (49%, 20 mg).

$^1$**H NMR** (600 MHz, Chloroform-*d*): δ = 7.91 – 7.85 (m, 4H), 7.65 – 7.59 (m, 1H), 7.52 (dd, *J* = 8.5, 7.4 Hz, 2H), 7.24 (d, *J* = 8.2 Hz, 2H), 3.30 (dt, *J* = 9.9, 7.8 Hz, 2H), 3.09 (dd, *J* = 9.7, 3.5 Hz, 1H), 2.96 (dd, *J* = 9.8, 3.5 Hz, 1H), 2.61 (tq, *J* = 7.7, 4.4 Hz, 2H), 2.38 (s, 3H), 1.74 (ddd, *J* = 12.8, 7.5, 5.5 Hz, 2H), 1.63 (dt, *J* = 12.1, 6.7 Hz, 1H), 1.48 (dt, *J* = 12.5, 6.5 Hz, 1H), 1.44 – 1.40 (m, 1H), 1.36 (dq, *J* = 10.4, 4.1, 3.1 Hz, 1H) ppm.

$^{13}$**C NMR** (151 MHz, Chloroform-*d*): δ = 142.6, 140.9, 135.3, 133.5, 129.1, 129.0, 127.7, 126.7, 126.6, 54.8, 54.6, 42.6, 42.5, 32.5, 32.4, 29.6, 25.9, 24.2, 21.5 ppm.

**HRMS** (ESI): *m/z*: $[M + Na]^+$ Calcd. for $C_{20}H_{24}N_2O_3S_2Na^+$: 427.11206; Found: 427.11194.

**Diethyl(3aR,6aS)-2-(N-((4-(5-methyl-3-phenylisoxazol-4-yl)phenyl)sulfonyl) phenylsulfonimidoyl)hexahydrocyclopenta[c]pyrrole-5,5(1H)-dicarboxylate (20c)**

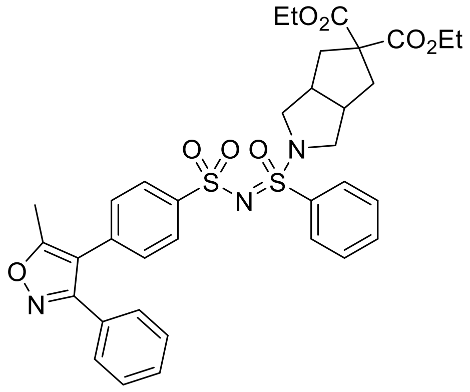

The title compound **20c** was synthesized according to the general procedure (GP-7), and was obtained after silica column chromatography (*n*-nentane : $Et_2O$ 2:1 – 1:1 – 1:2) as a yellow solid (43%, 30 mg).

$^1$**H NMR** (600 MHz, Chloroform-*d*): δ = 7.98 (d, *J* = 8.4 Hz, 2H), 7.95 – 7.90 (m, 2H), 7.65 (t, *J* = 7.5 Hz, 1H), 7.56 (t, *J* = 7.9 Hz, 2H), 7.40 – 7.36 (m, 3H), 7.34 – 7.31 (m, 2H), 7.27 – 7.25 (m, 2H), 4.22 – 4.05 (m, 4H), 3.38 – 3.34 (m, 1H), 3.20 (dt, *J* = 9.5, 7.3 Hz, 2H), 3.13 (dd, *J* = 9.9, 2.8 Hz, 1H), 2.77 (tdd, *J* = 11.9, 6.1, 2.0 Hz, 2H), 2.55 – 2.46 (m, 5H), 2.00 (dd, *J* = 13.5, 7.8 Hz, 1H), 1.89 (dd, *J* = 13.4, 7.9 Hz, 1H), 1.22 (td, *J* = 7.1, 4.6 Hz, 7H) ppm.

$^{13}$**C NMR** (151 MHz, Chloroform-*d*): δ = 171.5, 170.8, 167.1, 161.0, 142.6, 135.1, 134.5, 133.8, 129.8, 129.5, 129.3, 128.6, 128.4, 127.8, 127.1, 114.7, 62.0, 61.6, 53.8, 53.5, 41.9, 41.8, 39.3, 39.2, 14.0, 11.7 ppm.

**HRMS** (ESI): *m/z*: $[M + Na]^+$ Calcd. for $C_{35}H_{37}N_3O_8S_2Na^+$: 714.19143; Found: 714.19086.

**Diethyl(3a*R*,6a*S*)-2-(4-bromo-*N*-((4-bromophenyl)sulfonyl)phenylsulfonimidoyl) hexahydrocyclopenta[*c*]pyrrole-5,5(1*H*)-dicarboxylate (20d)**

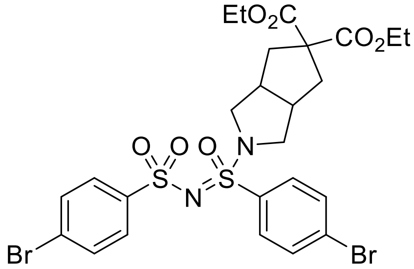


The title compound **20d** was synthesized according to the general procedure (GP-7), and was obtained after silica column chromatography (*n*-Pentane : $Et_2O$ 2:1 – 1:1 – 1:2) as a yellow solid (65%, 33 mg).

$^1$**H NMR** (600 MHz, Chloroform-*d*): δ = 7.83 (d, *J* = 8.6 Hz, 2H), 7.74 (d, *J* = 8.8 Hz, 2H), 7.68 (d, *J* = 8.7 Hz, 2H), 7.59 (d, *J* = 8.6 Hz, 2H), 4.23 – 4.09 (m, 4H), 3.30 (dd, *J* = 9.9, 2.7 Hz, 1H), 3.18 – 3.08 (m, 3H), 2.77 (dddd, *J* = 17.9, 10.7, 8.1, 2.7 Hz, 2H), 2.52 (dddd, *J* = 13.4, 11.8, 7.8, 6.4 Hz, 2H), 1.97 (dd, *J* = 13.5, 7.7 Hz, 1H), 1.89 (dd, *J* = 13.5, 7.8 Hz, 1H), 1.22 (t, *J* = 7.1 Hz, 6H) ppm.

$^{13}$**C NMR** (151 MHz, Chloroform-*d*) δ = 171.4, 170.8, 142.3, 134.1, 132.6, 131.8, 129.3, 129.2, 128.4, 127.1, 62.0, 61.68, 61.65, 53.8, 53.5, 41.9, 41.8, 39.3, 39.2, 14.0, 13.9 ppm.

**HRMS** (ESI): *m/z*: $[M + Na]^+$ Calcd. for $C_{25}H_{28}N_2O_7S_2Br_2Na^+$: 714.95764; Found: 714.95755.

## 13. References


[1] S. Pan, F. F. Mulks, P. Wu, K. Rissanen, C. Bolm, *Angew. Chem. Int. Ed.* **2024**, *63*, e202316702.

[2] Y.-Z. Ji, J.-Y. Zhang, H.-J. Li, C. Han, Y.-K. Yanga, Y.-C. Wu, *Org. Biomol. Chem.* **2019**,*17*, 4789-4800.

[3] P. Gross, H. Im, D. Laws III, B. Park, M.-H. Baik, S. B. Blakey, *J. Am. Chem. Soc.* **2024**, *146*, 1447–1454.

[4] H. Okamura, C. Bolm, *Org. Lett.* **2004**, *6*, 1305–1307

[6] J. W. W. Chang, T. M. U. Ton, Z. Zhang, Y. Xu, P. W. H. Chan, *Tetrahedron Lett.* **2009**, *50,* 161-164.

[6] F. Li W. F. Zhu, C. Empel, O. Datsenko, A. Kumar, Y. Xu, J. H. M. Ehrler, I. Atodiresei, S. Knapp, P. K. Mykhailiuk, E. Proschak, R. M. Koenigs *Science,* **2024**, *383*, 498-503.

[7] M. Arık, N. Çelebi, Y. Onganer, *J. Photochem. Photobiol., A* **2005**, *170*, 105-111.

[8] T. H.-F. Wong, D. Ma, R. D. Sanza, P. Melchiorre, *Org. Lett*. **2022**, *24*, 1695–1699.

[9] M. Montalti, A. Credi, L. Prodi, M. T. Gandolfi, Handbook of Photochemistry, Taylor
Francis, **2006**.

[10] C. Liang, Y. Guo, Y. Zhang, Z. Wang, L. Li, W. Li, *Org. Chem. Front.* **2023**, *10*,611−623.

[11] Stoe&Cie, **2024**.

[12] G. M. Sheldrick, *Acta Crystallogr A Found Adv* **2015**, *71*, 3–8.

[13] O. V. Dolomanov, L. J. Bourhis, R. J. Gildea, J. A. K. Howard, H. Puschmann, *J Appl Crystallogr* **2009**, *42*, 339–341.

[14] F. Kleemiss, O. V. Dolomanov, M. Bodensteiner, N. Peyerimhoff, L. Midgley, L. J. Bourhis, A. Genoni, L. A. Malaspina, D. Jayatilaka, J. L. Spencer, F. White, B. Grundkötter-Stock, S. Steinhauer, D. Lentz, H. Puschmann, S. Grabowsky, *Chem. Sci.* **2021**, *12*, 1675–1692.

[15] J. W. Furness, A. D. Kaplan, J. Ning, J. P. Perdew, J. Sun, *J. Phys. Chem. Lett.* **2020**, *11*, 8208–8215.

[16] F. Neese, F. Wennmohs, U. Becker, C. Riplinger, *J. Chem. Phys.* **2020**, *152*, 224108.

[17] F. Neese, *WIREs Comput. Mol. Sci.* **2022**, *12*, e1606.

[18] F. Neese, *J. Comput. Chem.* **2003**, *24*, 1740–1747.

[19] F. Neese, *J. Comput. Chem.* **2023**, *44*, 381–396.

[20] S. Lehtola, C. Steigemann, M. J. T. Oliveira, M. A. L. Marques, *SoftwareX* **2018**, *7*, 1–5.

[21] G. A. R. Y. Suaifan, M. Shehadeh, D. Tahboub, A.A. M. Mohammed, M. D. Threadgill, A. Gaurav, M. Khan *et al.* *Med Chem Res,* **2024**, *33*, 663–676.

## 14. NMR Spectra

### *N*-(Pivaloyloxy)-4-(5-(*p*-tolyl)-3-(trifluoromethyl)-1*H*-pyrazol-1-yl)benzenesulfonamide

[1]**H NMR** (600 MHz, Chloroform-*d*)

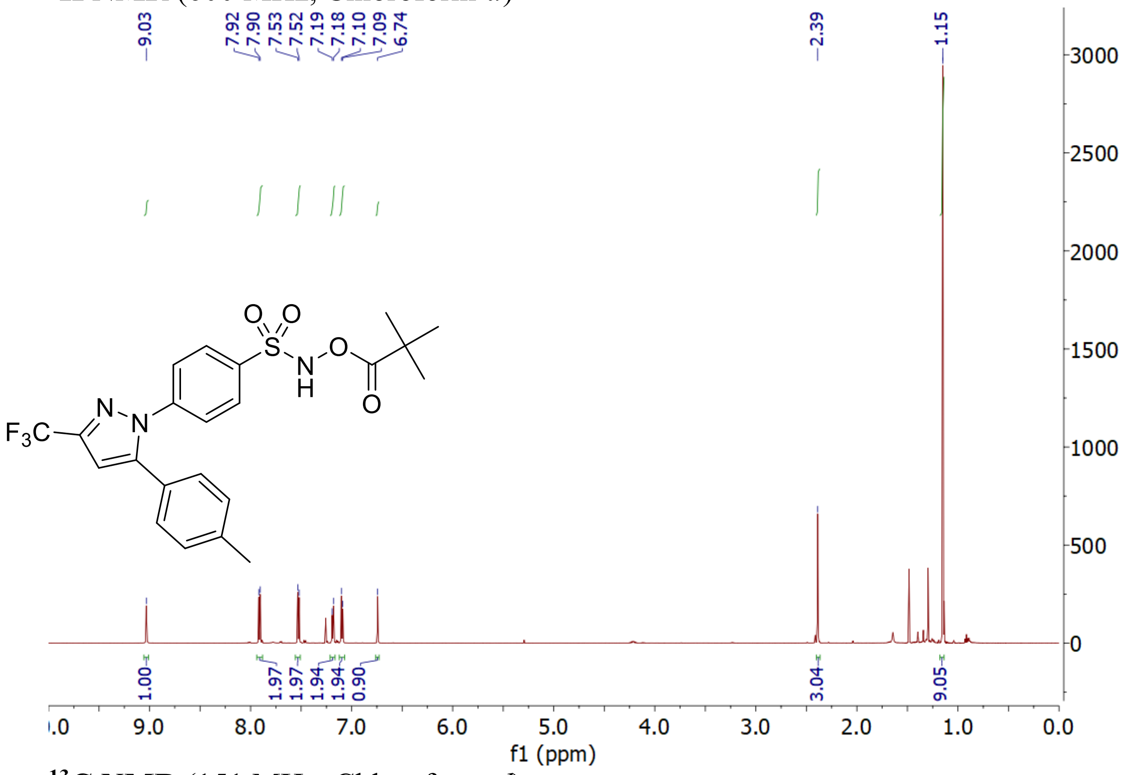


[13]**C NMR** (151 MHz, Chloroform-*d*)

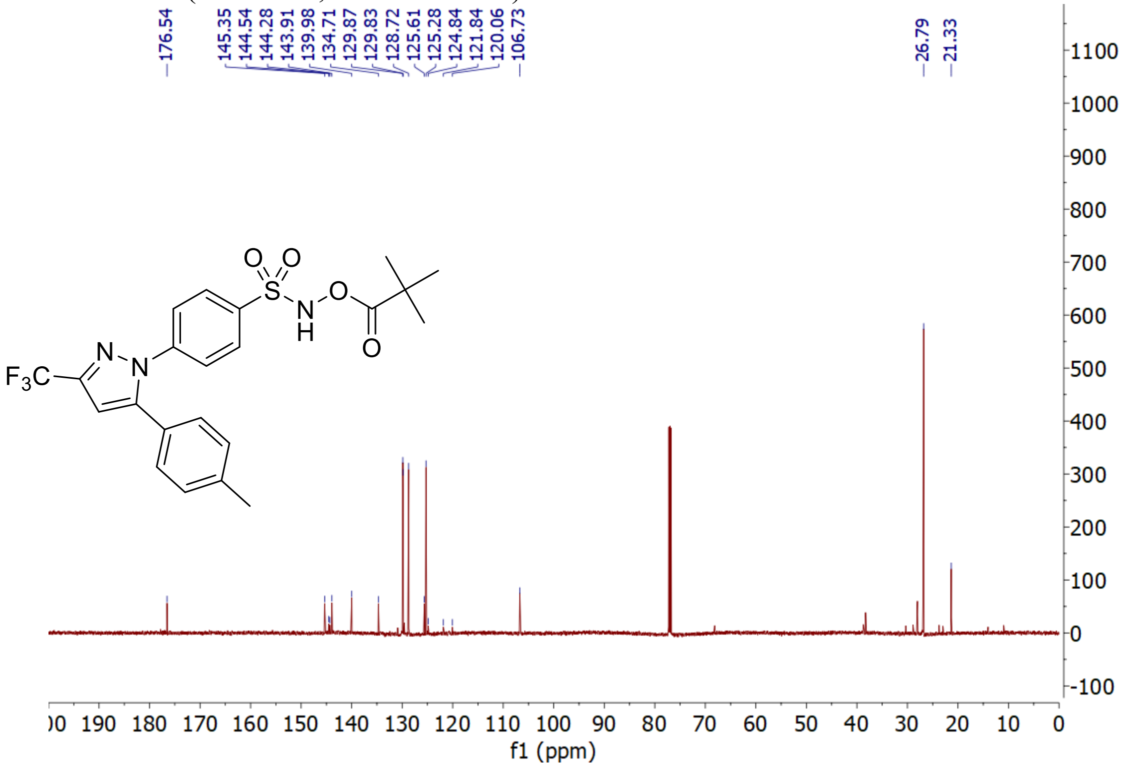

**$^{19}$F NMR** (564 MHz, $CDCl_3$)

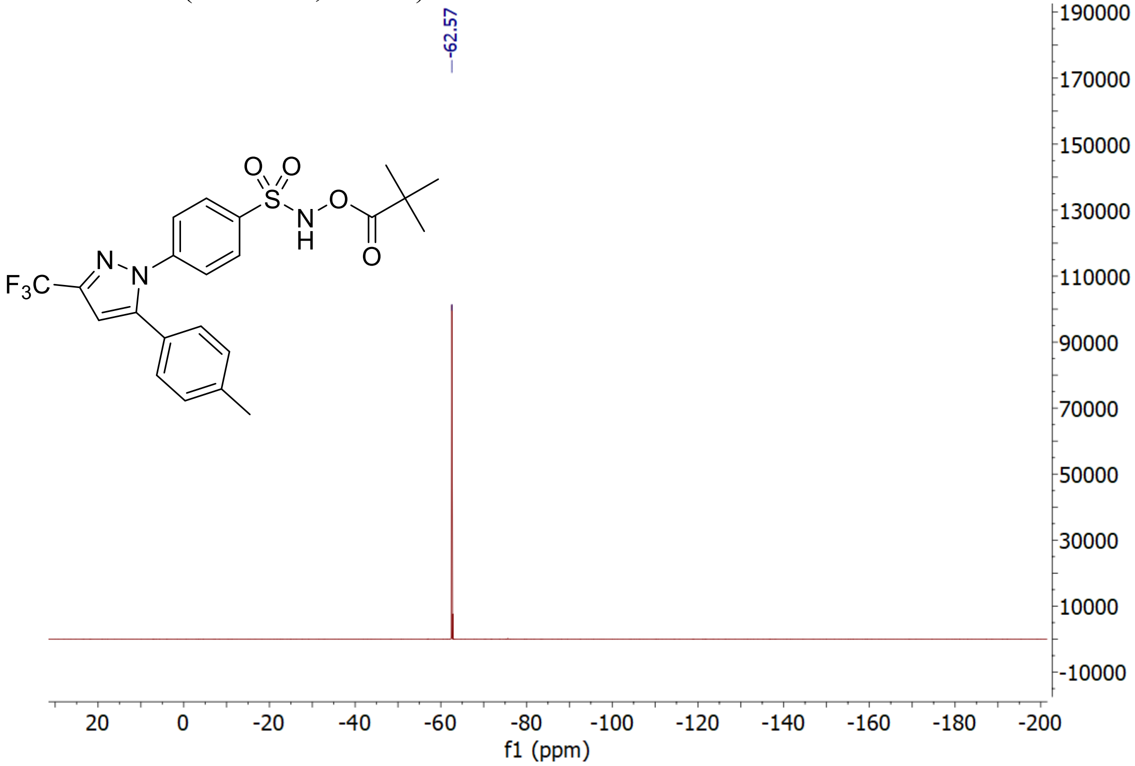

**4-(5-Methyl-3-phenylisoxazol-4-yl)-*N*-(pivaloyloxy)benzenesulfonamide**

$^1$**H NMR** (600 MHz, DMSO-$d_6$)

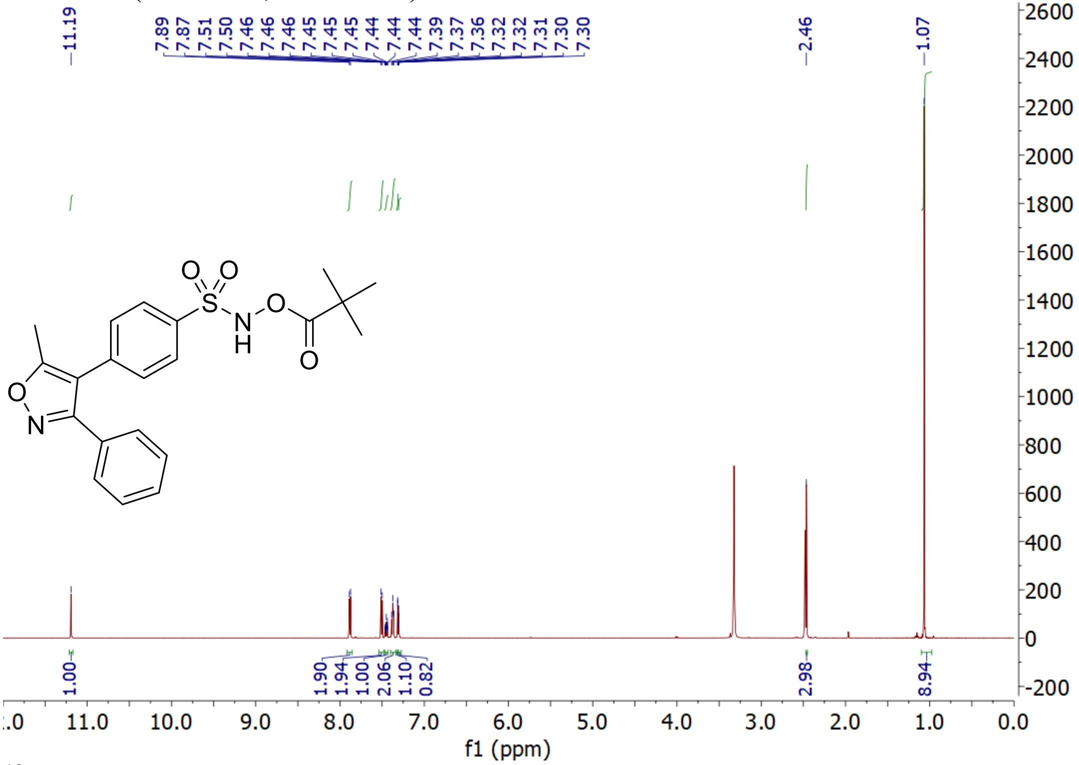

$^{13}$**C NMR** (151 MHz, DMSO-$d_6$)

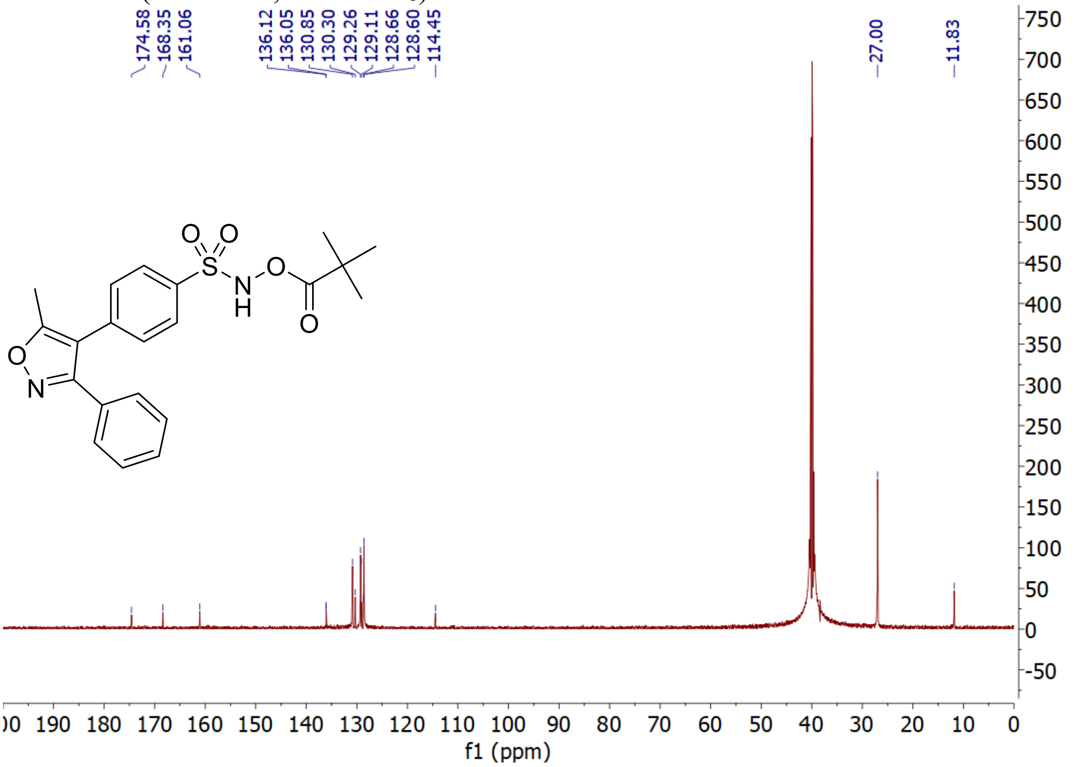

**4-(5-Methyl-3-phenylisoxazol-4-yl)benzenesulfinamide**

**$^{1}$H NMR** (600 MHz, Chloroform-*d*):

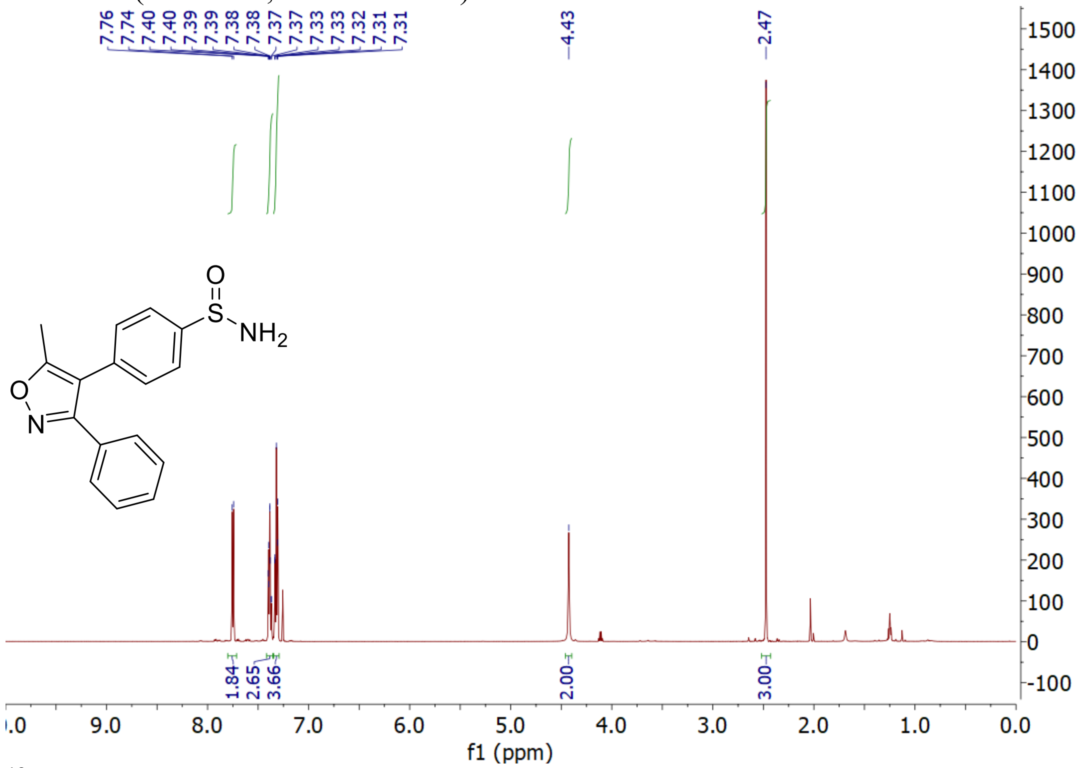


**$^{13}$C NMR** (151 MHz, Chloroform-*d*):

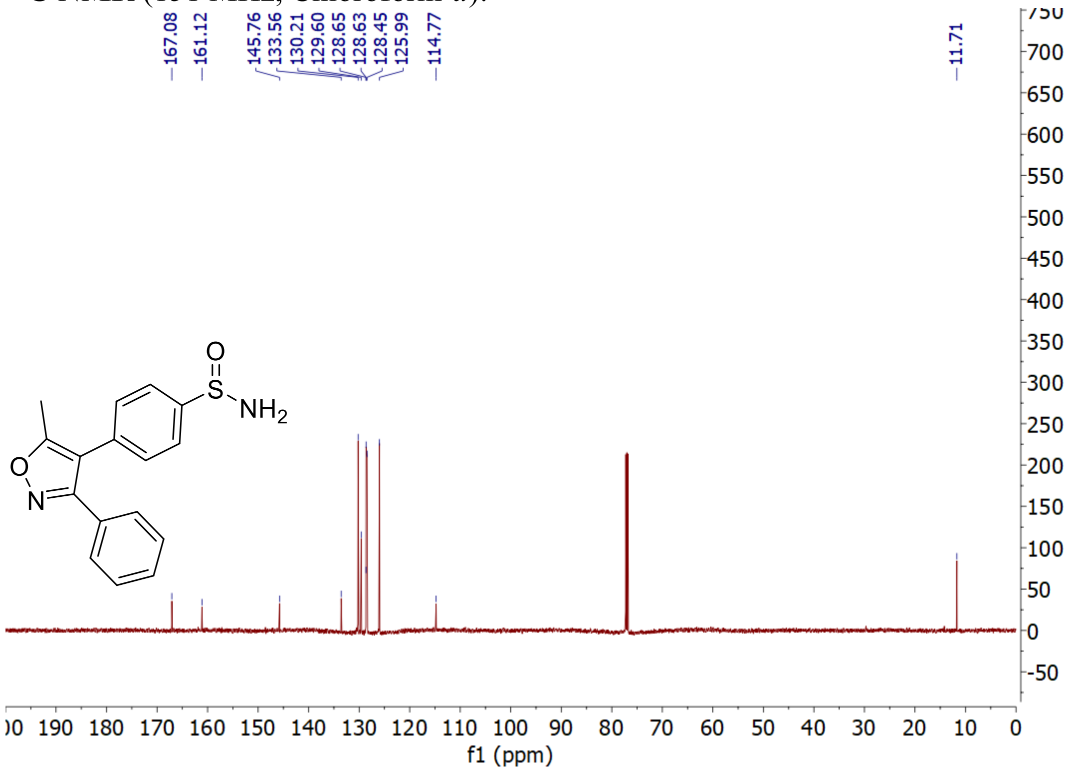

*N*-(Amino(oxo)(phenyl)- λ⁶-sulfaneylidene)-4-methylbenzenesulfonamide (12a)

[1]**H NMR** (600 MHz, DMSO-$d_6$)

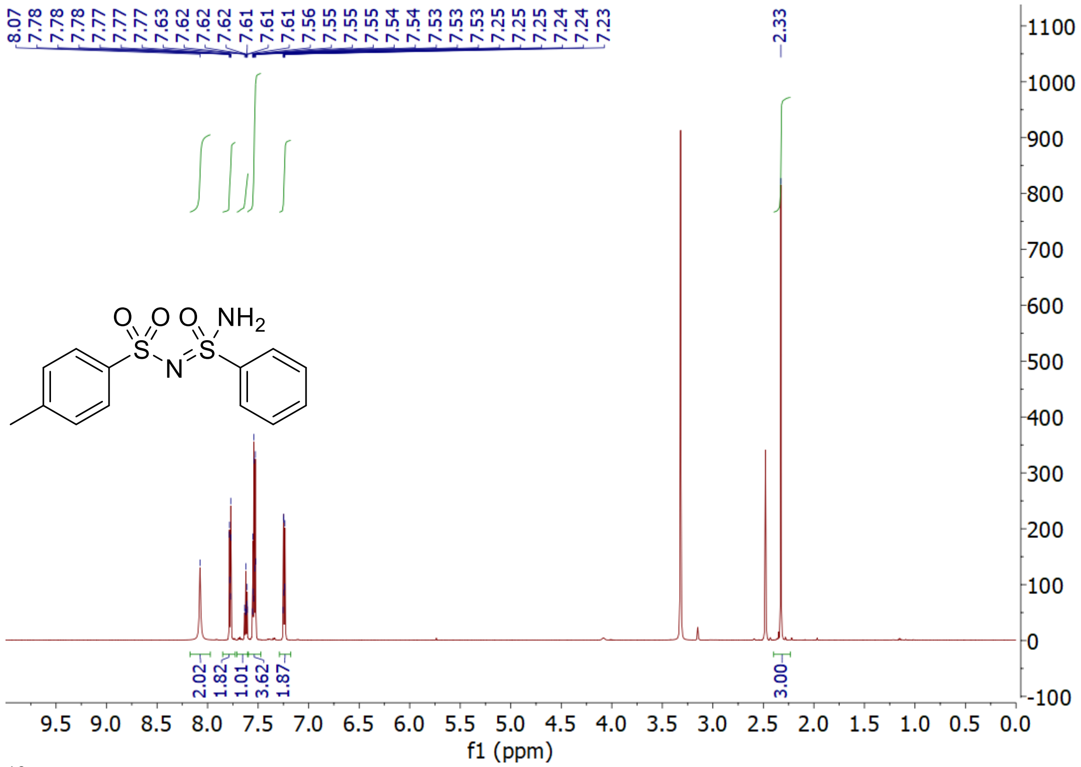

[13]**C NMR** (151 MHz, DMSO-$d_6$)

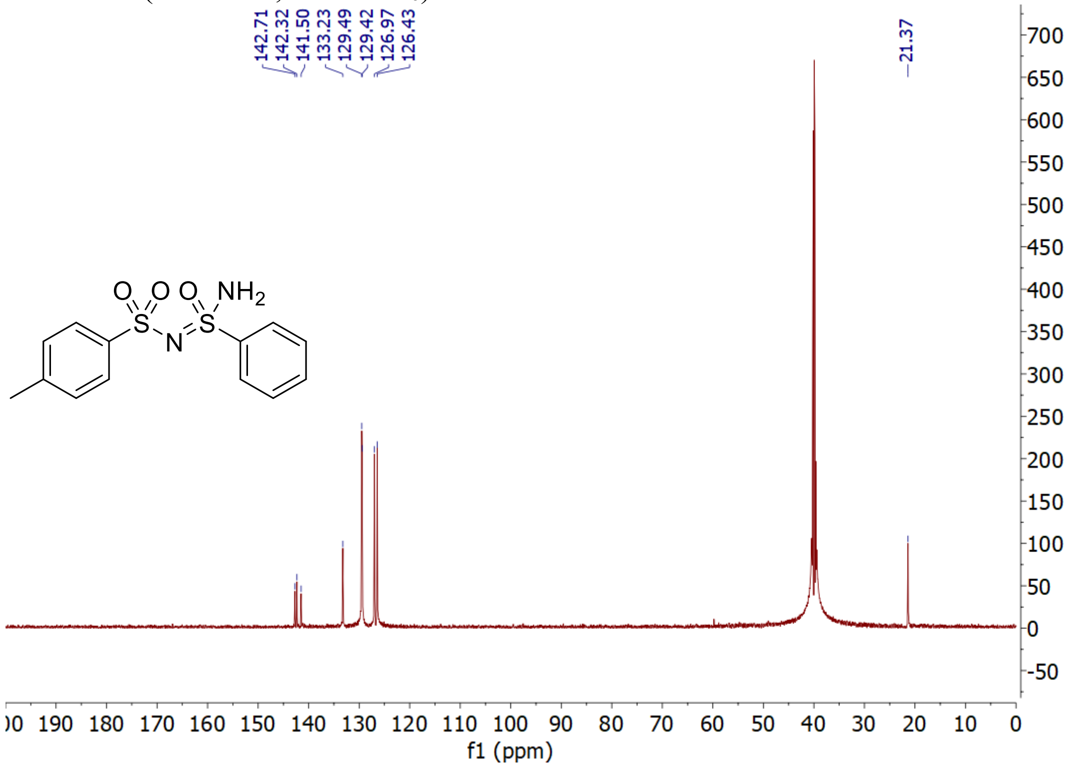

**$N$-(Amino(oxo)(phenyl)-$\lambda^6$-sulfaneylidene)benzenesulfonamide (12b)**

**$^1$H NMR** (600 MHz, DMSO-$d_6$)

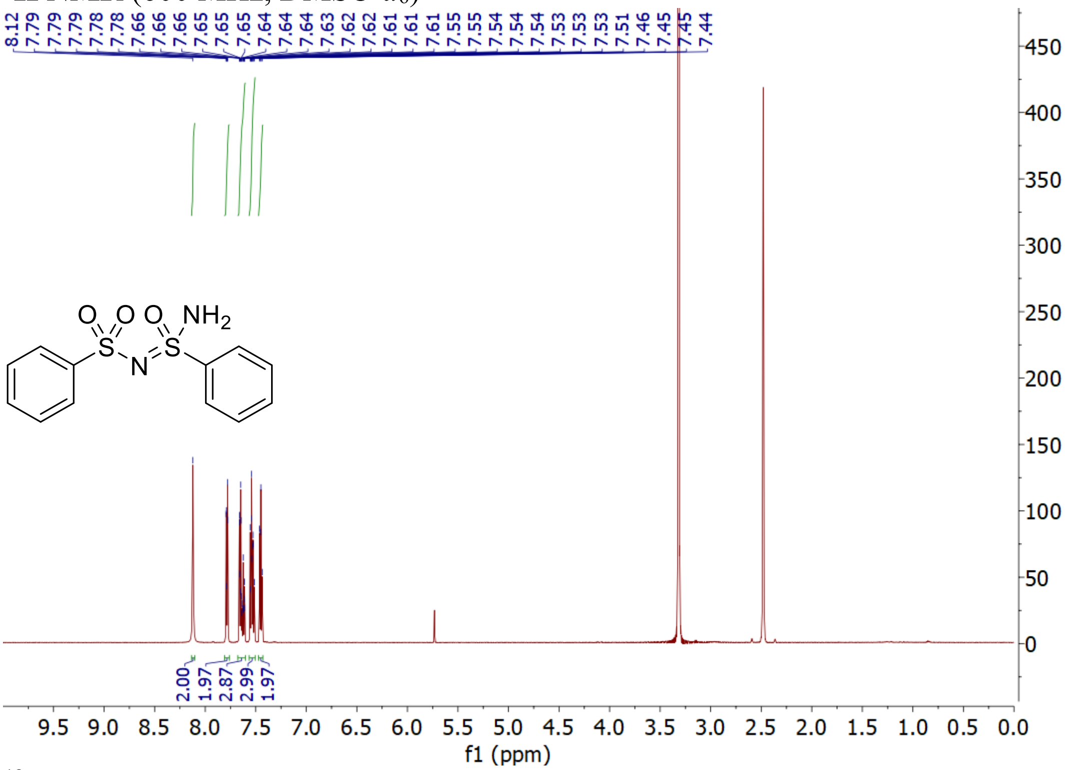

**$^{13}$C NMR** (151 MHz, DMSO-$d_6$)

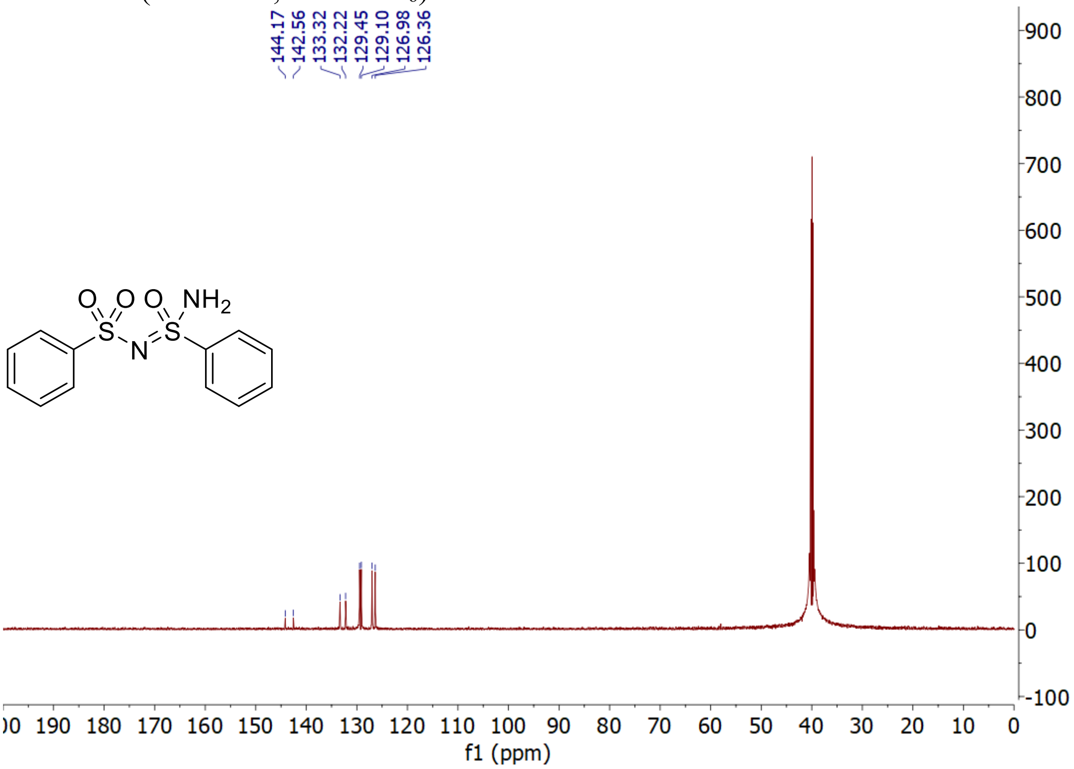

***N*-(Amino(oxo)(phenyl)-λ⁶-sulfaneylidene)-4-fluorobenzenesulfonamide (12c)**

**$^{1}$H NMR** (400 MHz, DMSO-$d_6$)

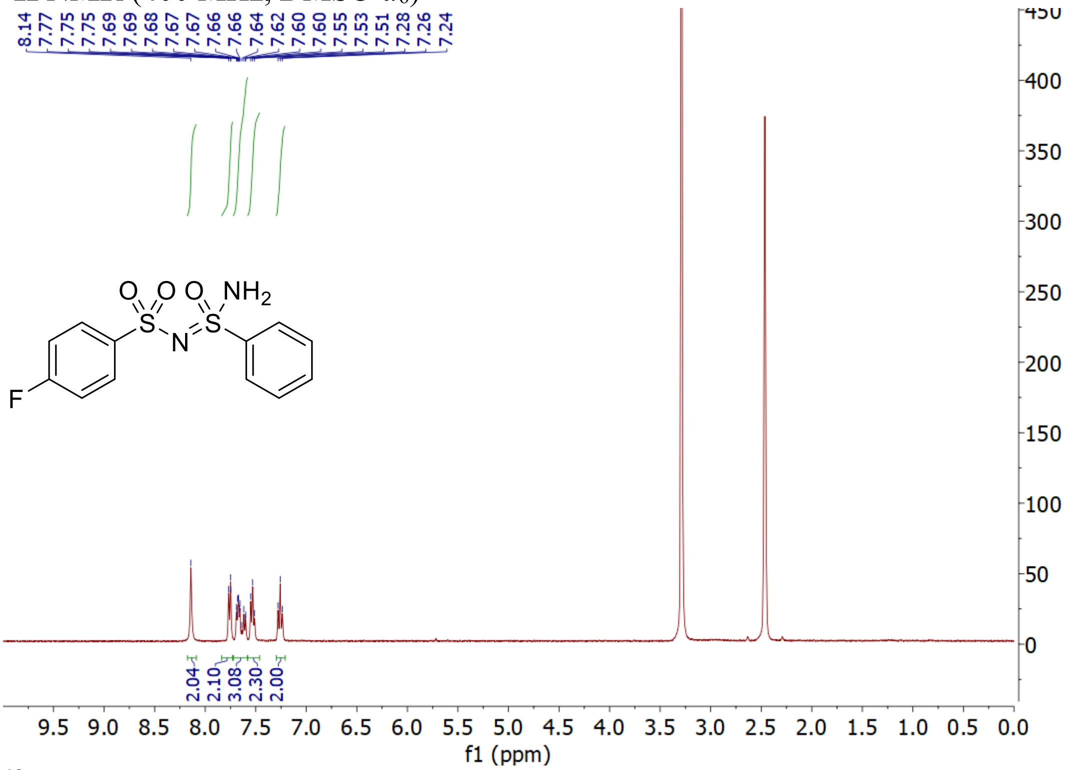


**$^{13}$C NMR** (151 MHz, DMSO-$d_6$)

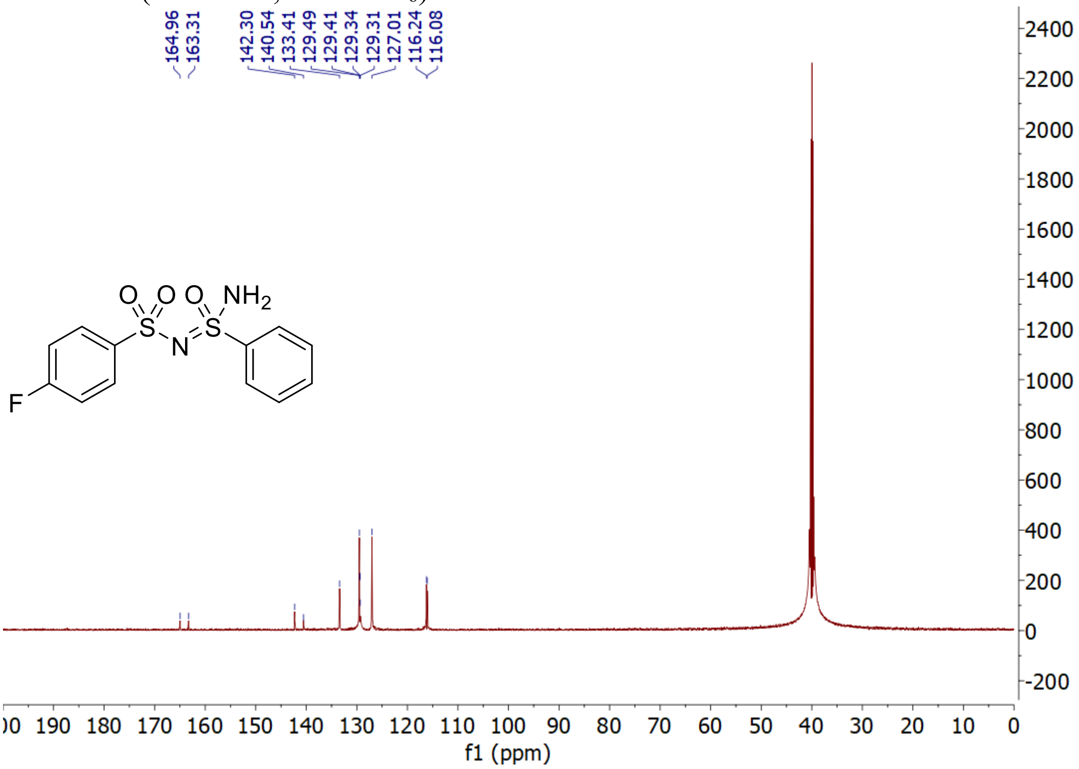

**$^{19}$F NMR** (564 MHz, DMSO-$d_6$)

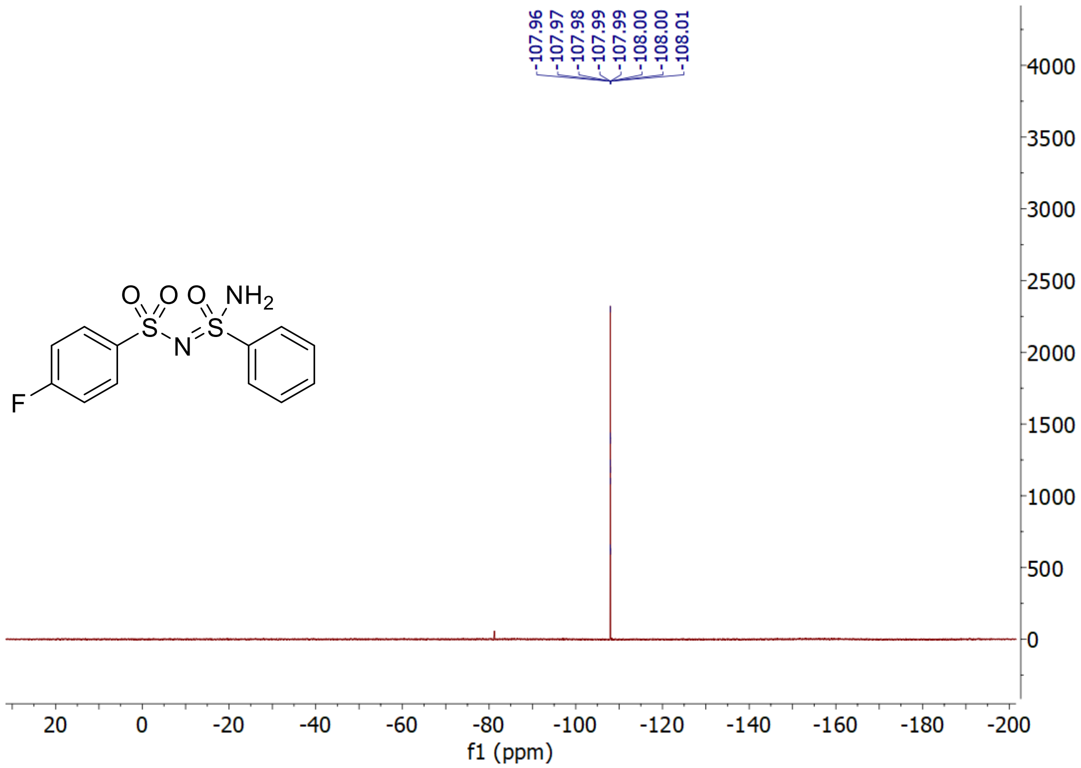

***N*-(Amino(oxo)(phenyl)-λ$^6$-sulfaneylidene)-4-bromobenzenesulfonamide (12d)**

**$^1$H NMR** (600 MHz, DMSO-$d_6$)

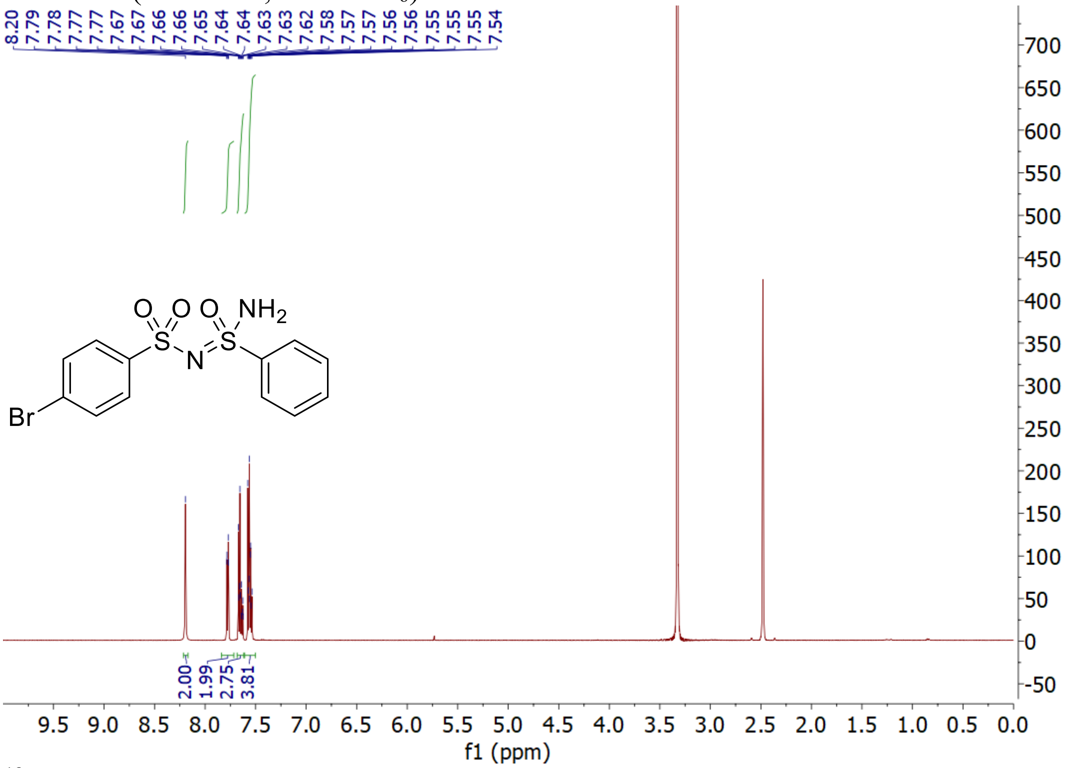


**$^{13}$C NMR** (151 MHz, DMSO-$d_6$)

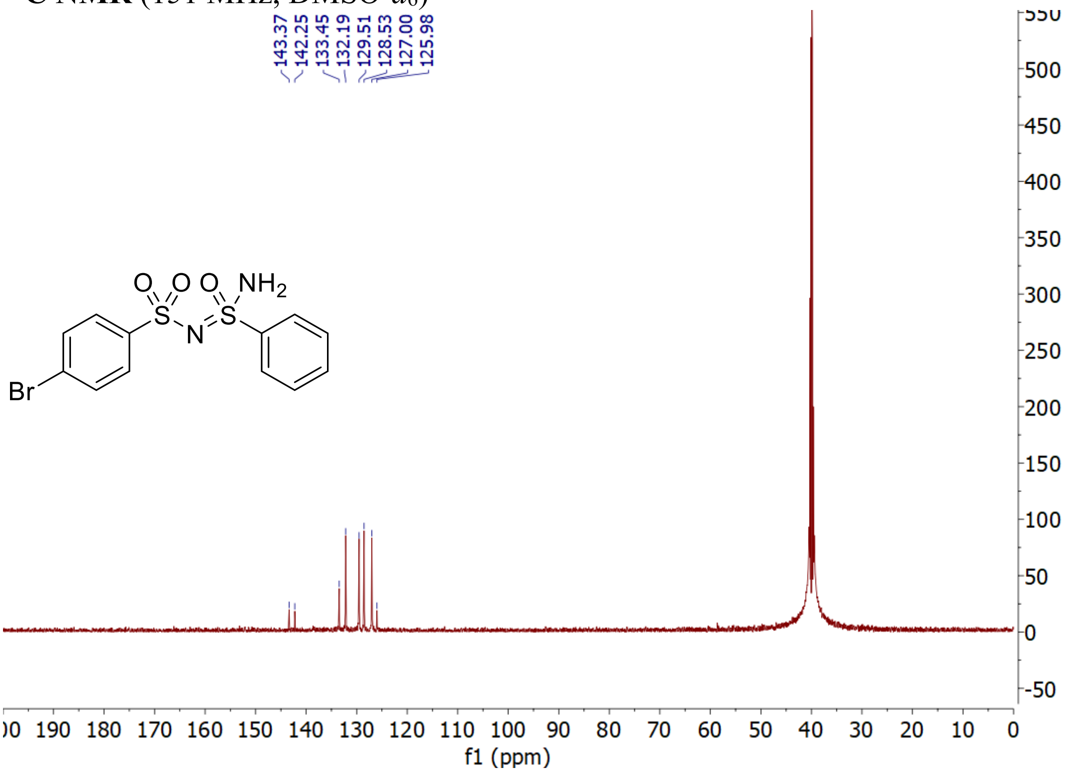

***N*-(Amino(oxo)(phenyl)-λ⁶-sulfaneylidene)-4-(trifluoromethyl) benzenesulfonamide (12e)**

$^{1}$**H NMR** (600 MHz, DMSO-$d_6$)

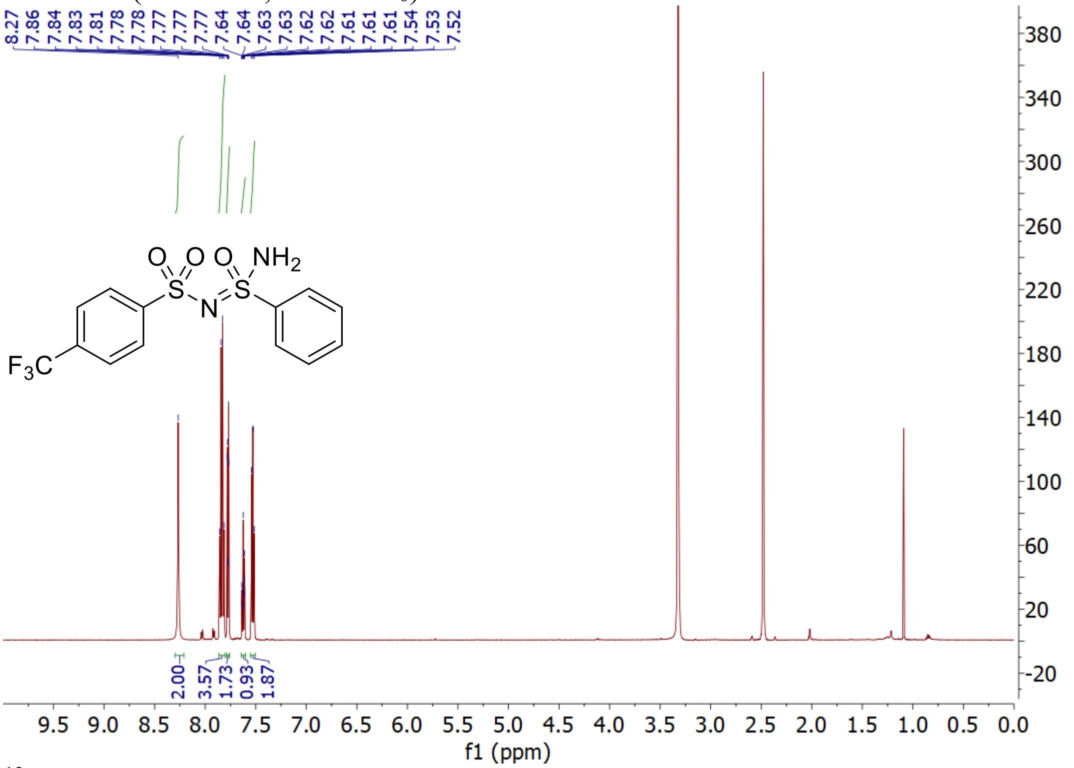

$^{13}$**C NMR** (151 MHz, DMSO-$d_6$)

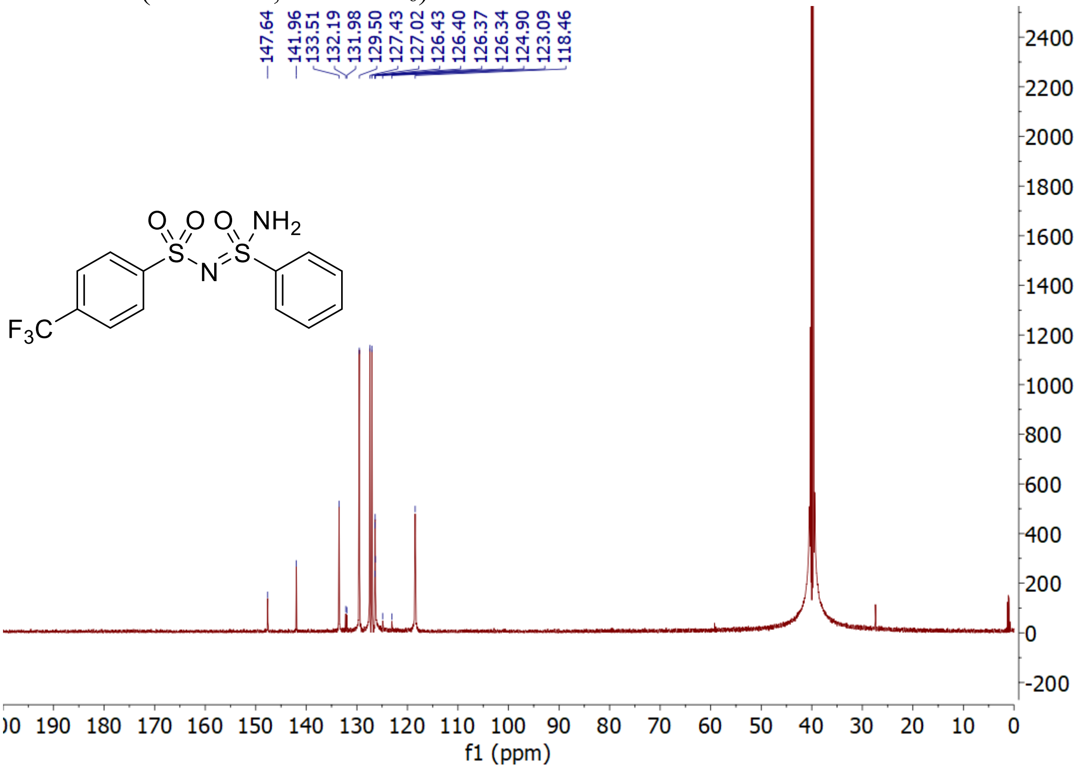

**$^{19}$F NMR** (564 MHz, DMSO-$d_6$)

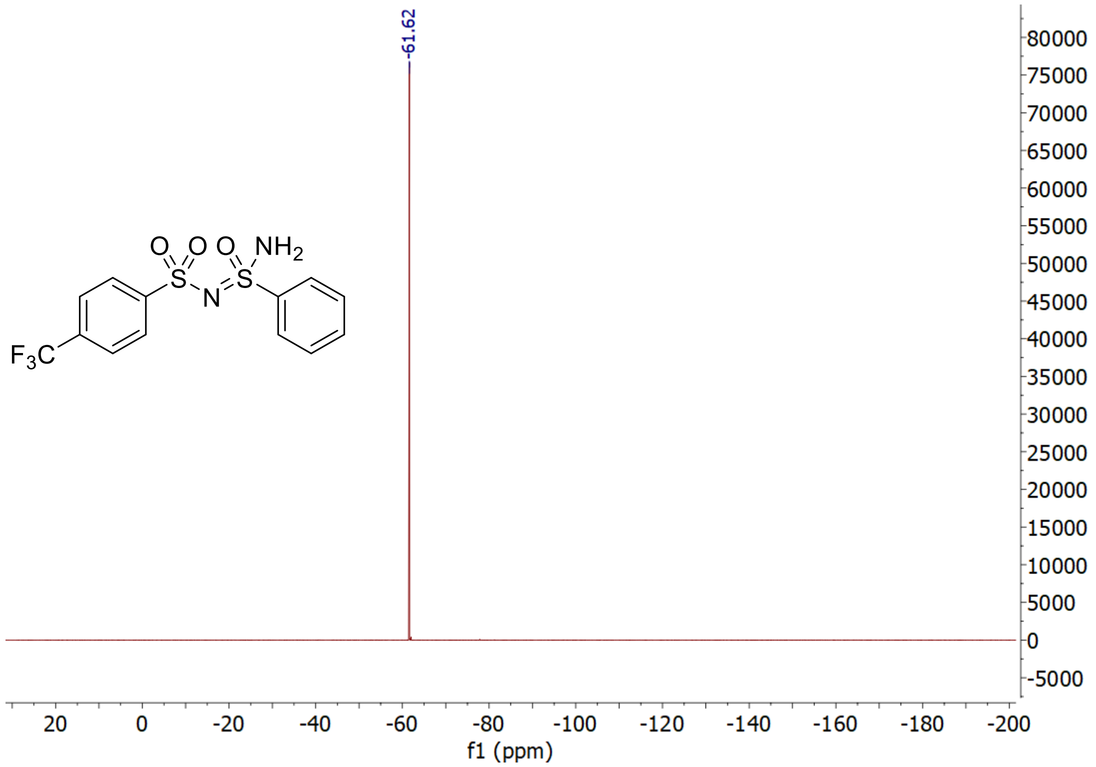

***N*-(Amino(oxo)(phenyl)-λ⁶-sulfaneylidene)-3-chlorobenzenesulfonamide (12f)**

**$^{1}$H NMR** (600 MHz, DMSO-$d_6$)

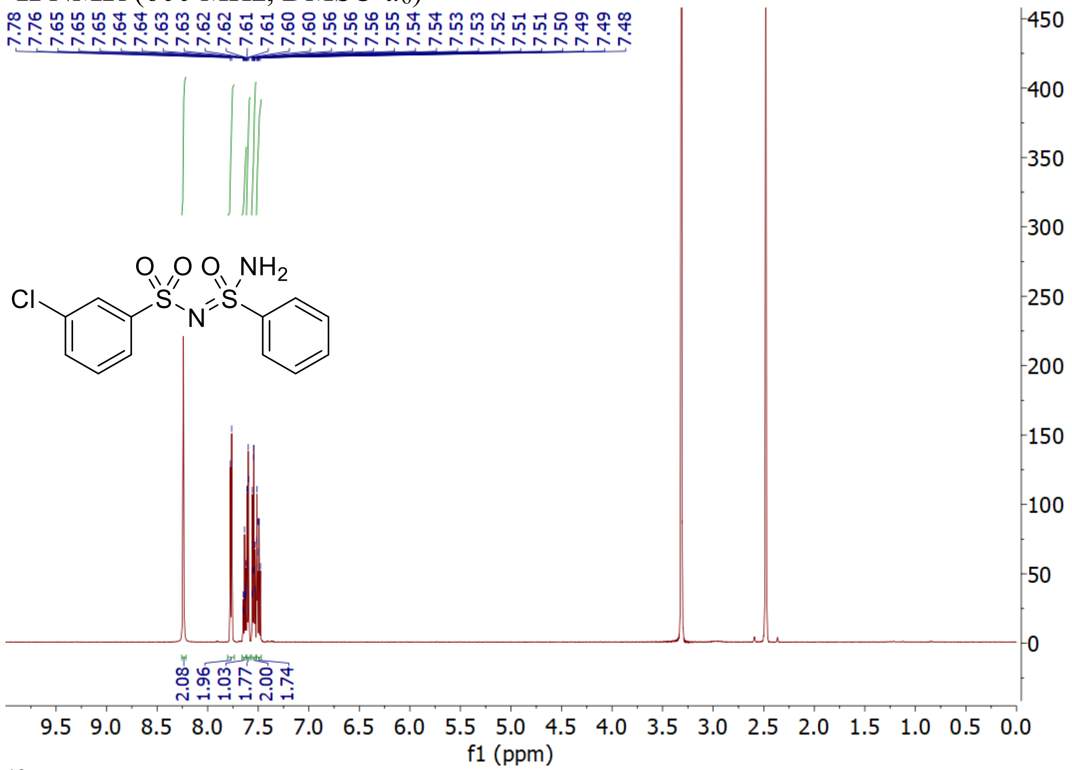

**$^{13}$C NMR** (151 MHz, DMSO-$d_6$)

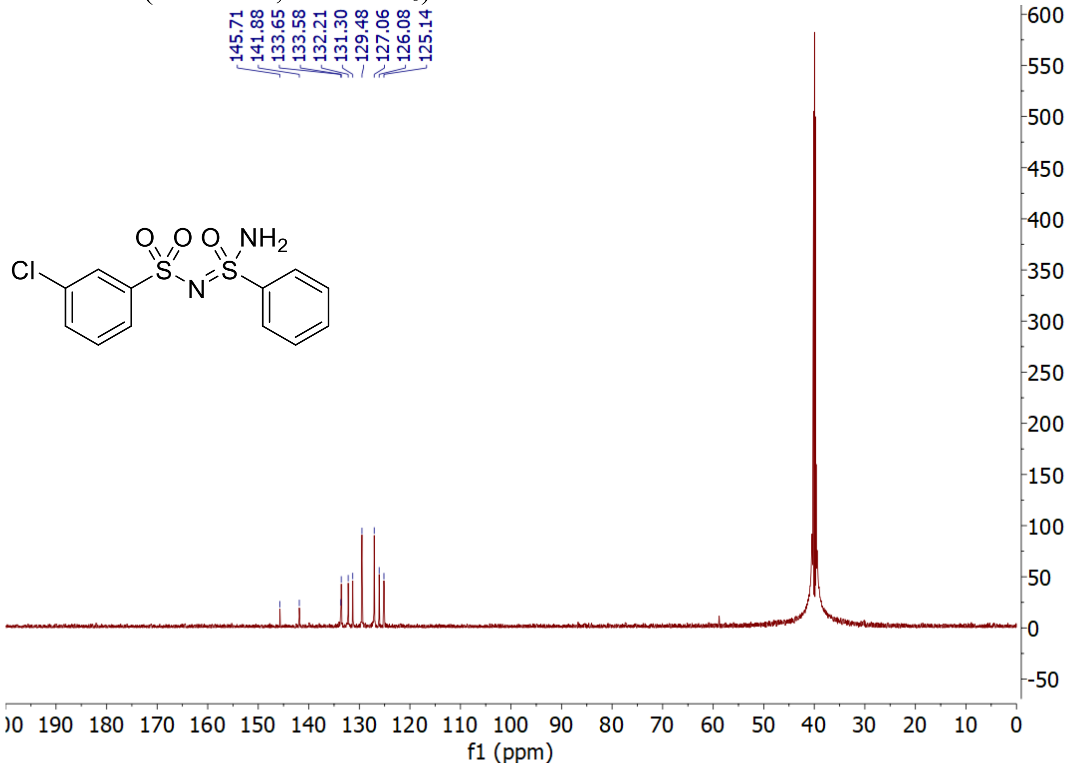

***N*-(Amino(oxo)(phenyl)-λ⁶-sulfaneylidene)-3-nitrobenzenesulfonamide (12g)**

$^1$**H NMR** (600 MHz, DMSO-$d_6$)

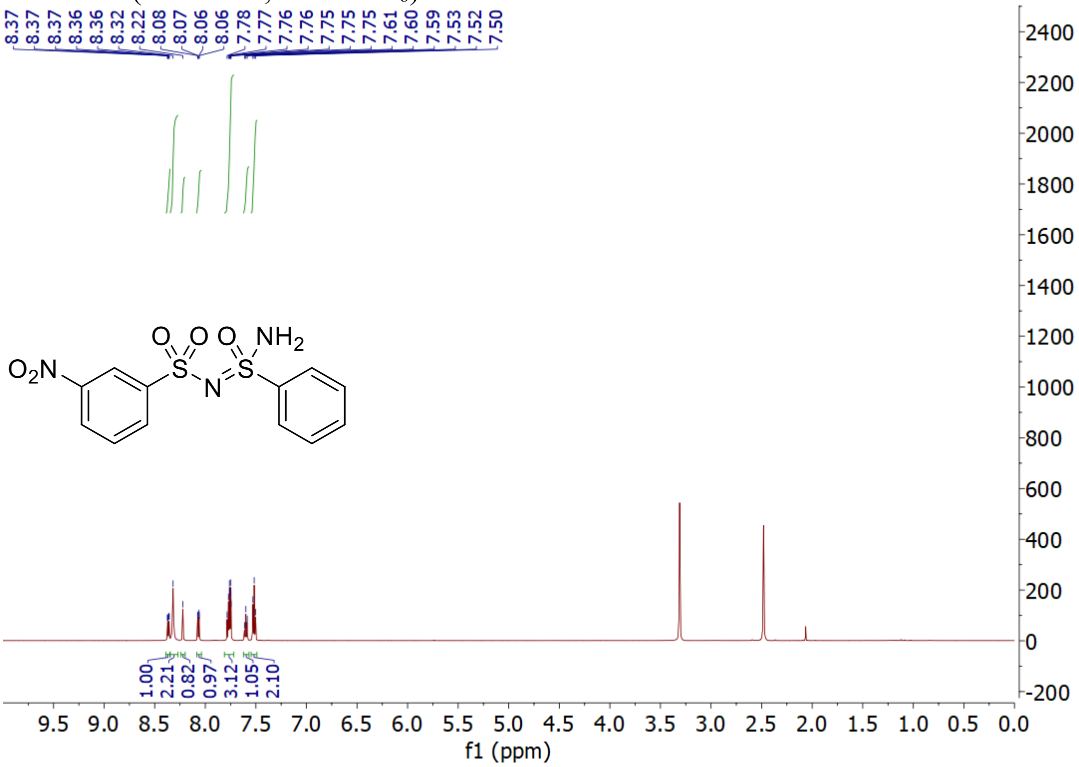


$^{13}$**C NMR** (151 MHz, DMSO-$d_6$)

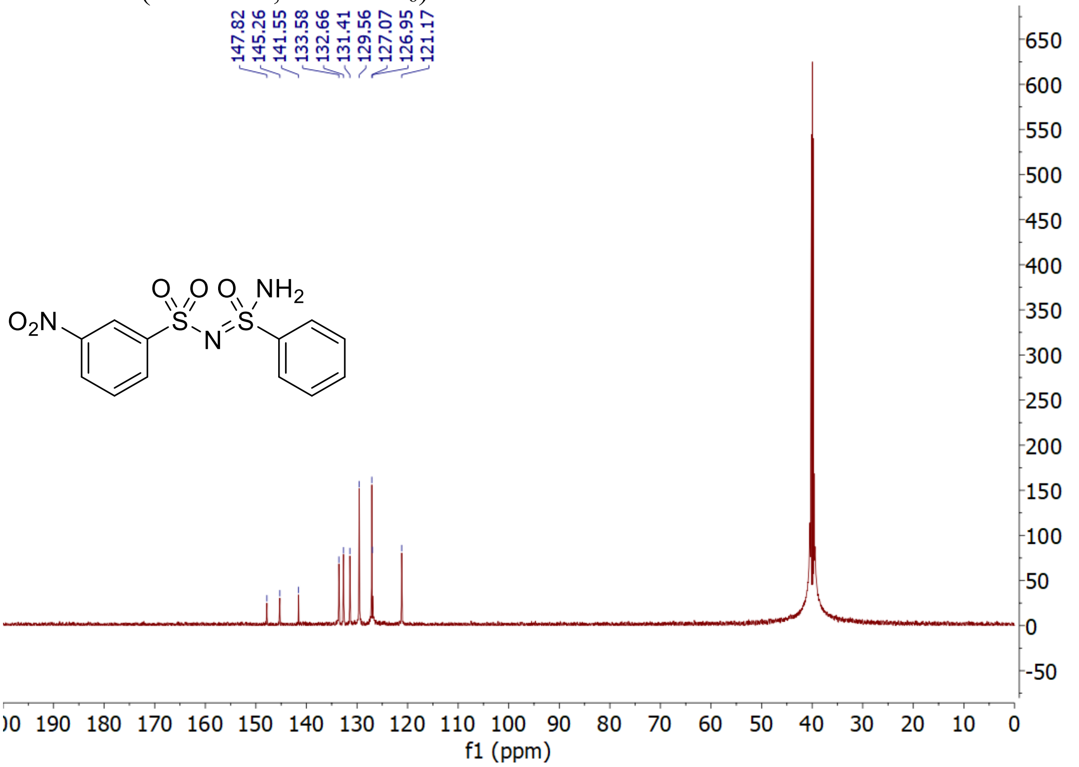

**$N$-(Amino(oxo)(phenyl)-λ$^6$-sulfaneylidene)-3-chloro-2-methylbenzenesulfonamide (12h)**

**$^1$H NMR** (600 MHz, DMSO-$d_6$)

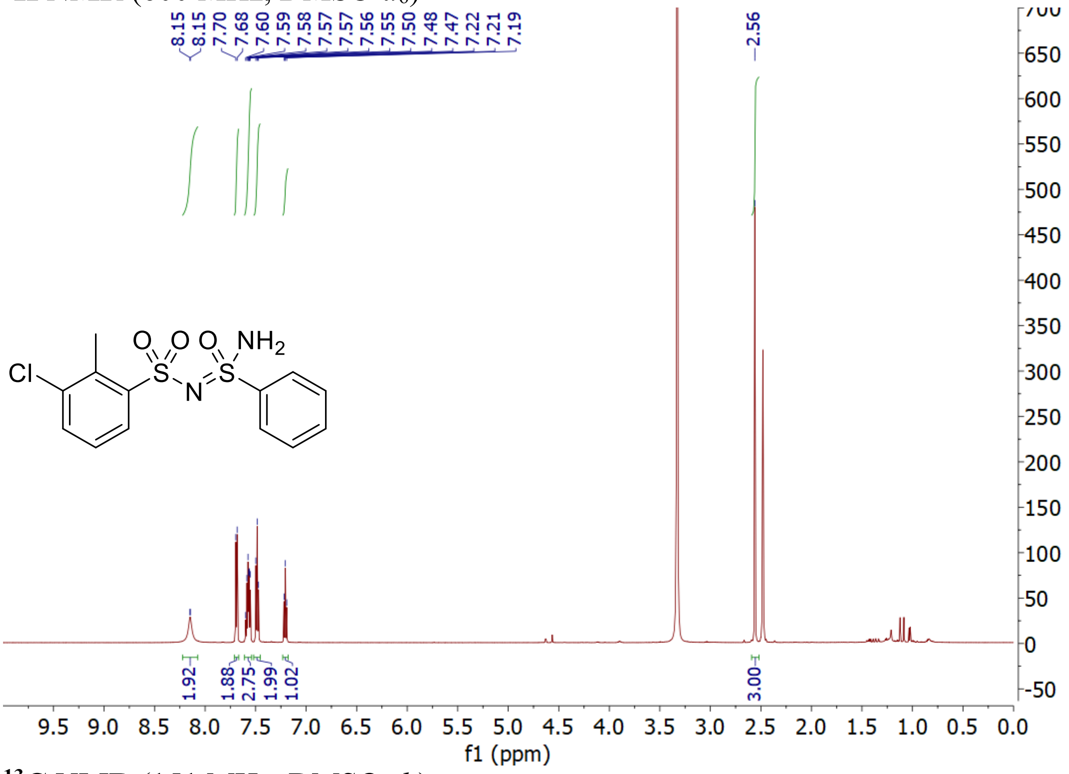


**$^{13}$C NMR** (151 MHz, DMSO-$d_6$)

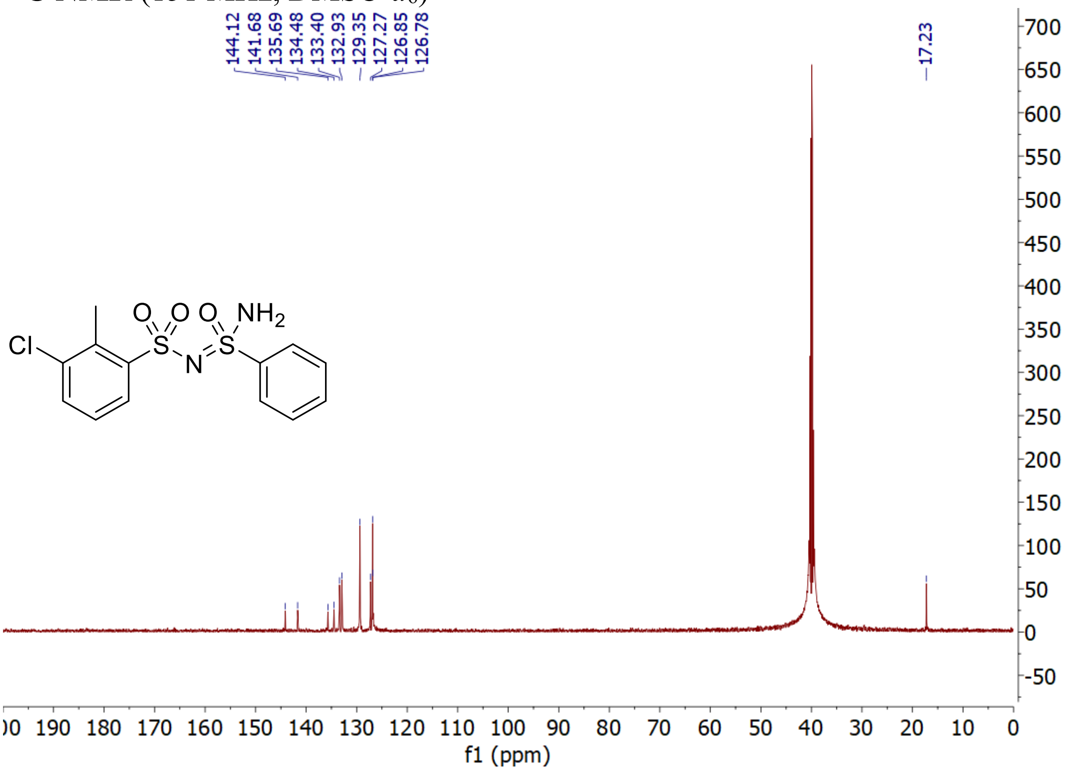

***N*-(Amino(4-bromophenyl)(oxo)-λ⁶-sulfaneylidene)-4-bromobenzenesulfonamide (12i)**

[1]**H NMR** (600 MHz, DMSO-*d*6)

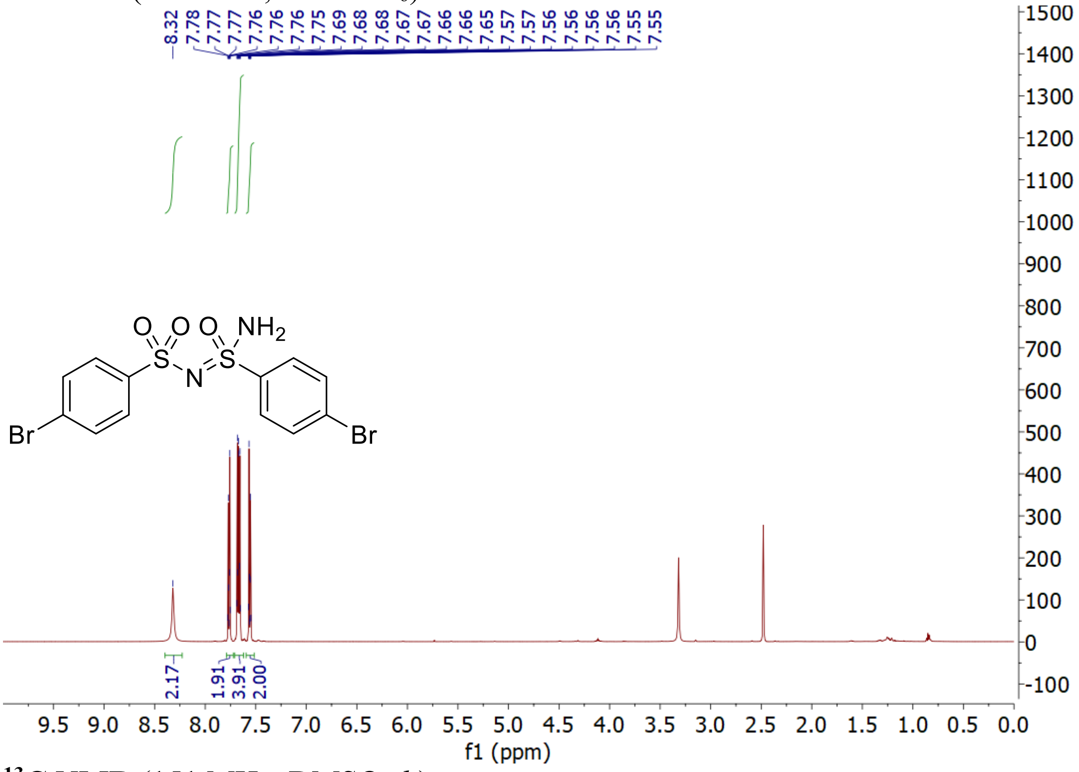

[13]**C NMR** (151 MHz, DMSO-*d*6)

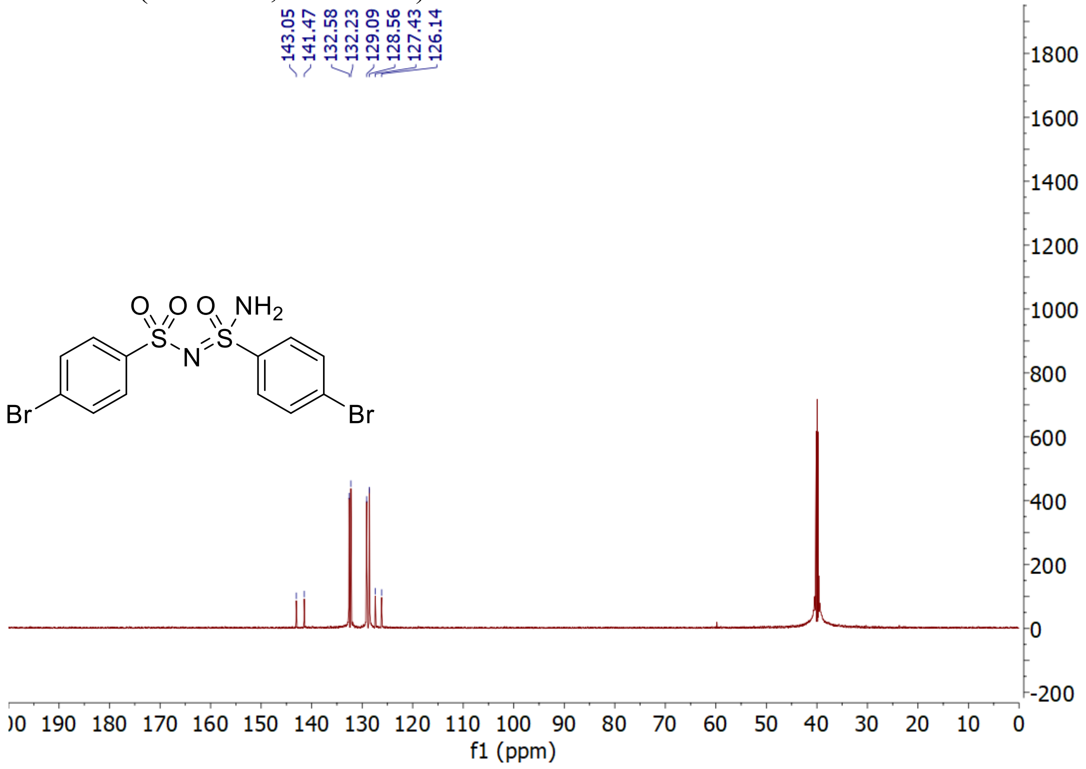

*N*-(Amino(oxo)(phenyl)-λ[6]-sulfaneylidene)pyridine-3-sulfonamide (12j)

[1]**H NMR** (400 MHz, DMSO-$d_6$)

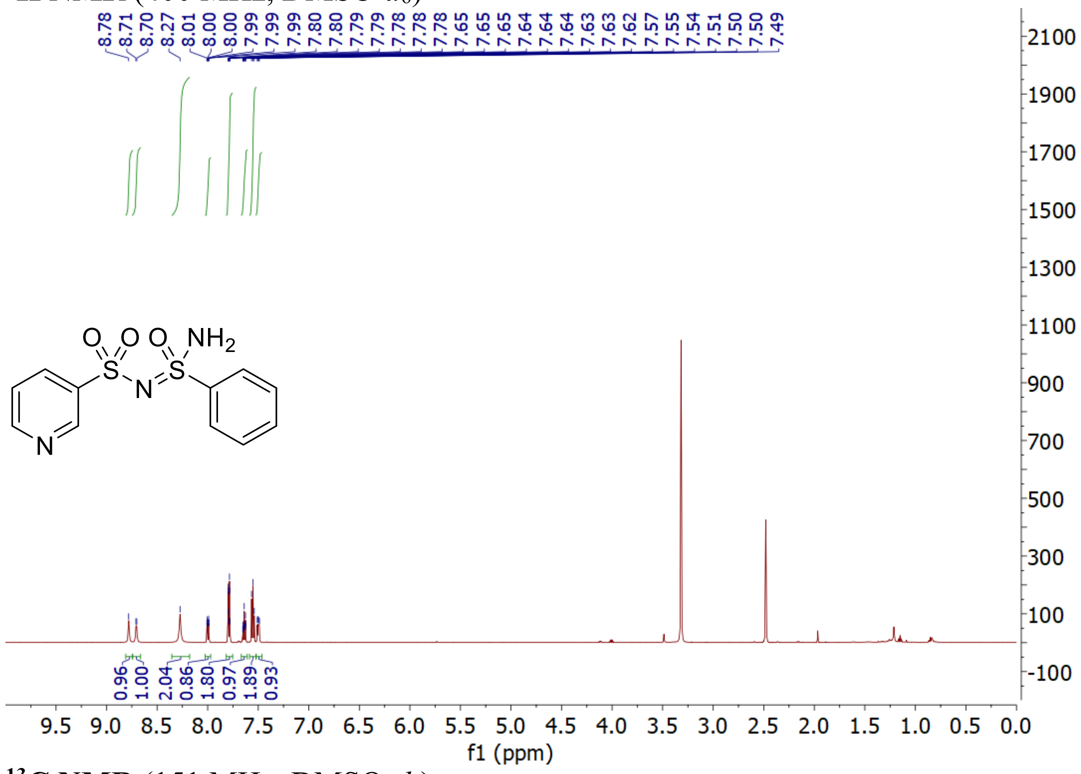

[13]**C NMR** (151 MHz, DMSO-$d_6$)

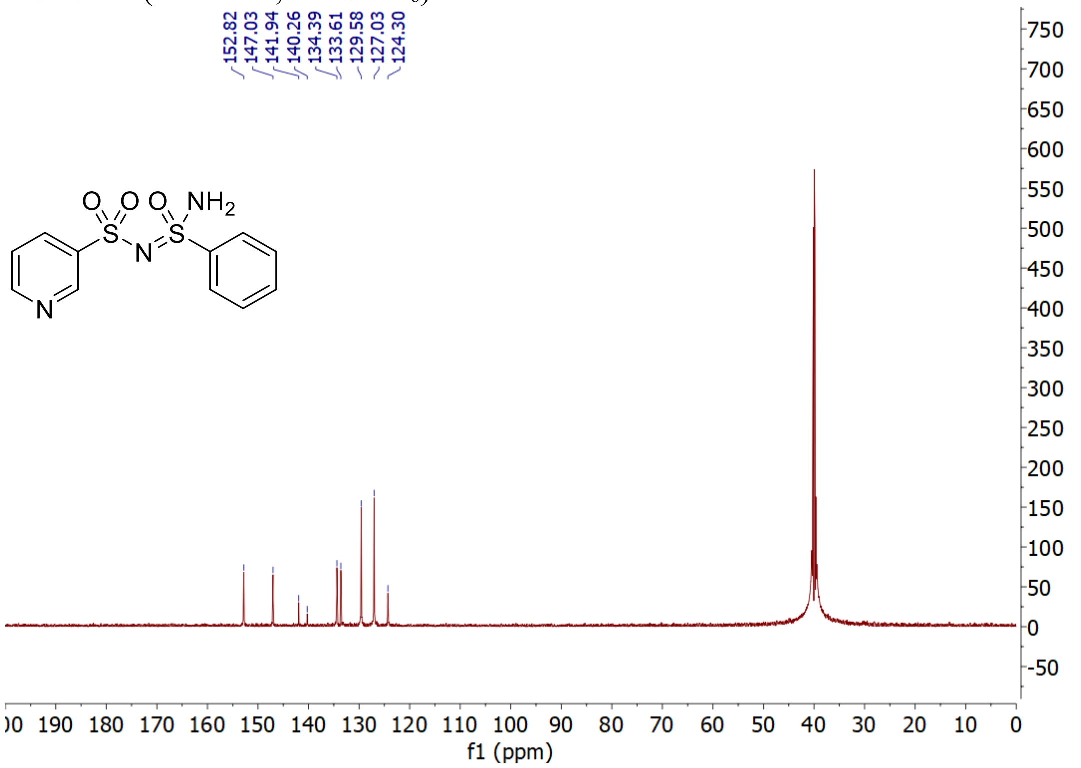

**(*E*)-*N*-(Amino(oxo)(phenyl)-λ⁶-sulfaneylidene)-2-phenylethene-1-sulfonamide (12k)**

$^{1}$**H NMR** (600 MHz, DMSO-$d_6$)

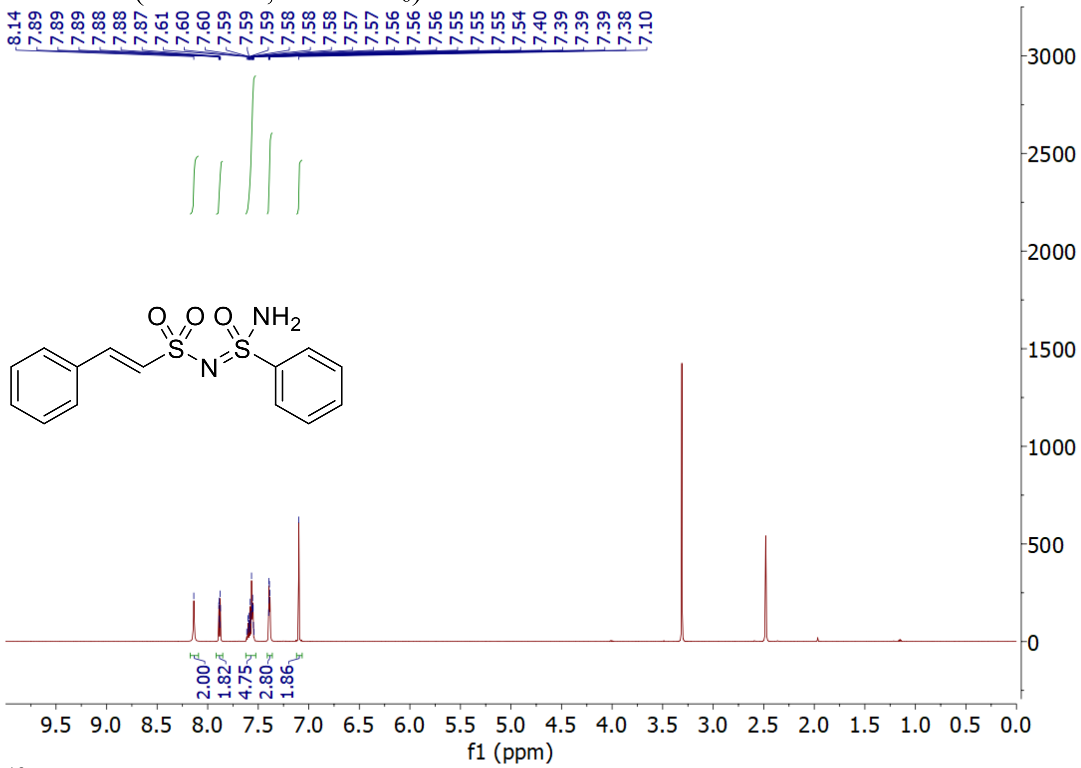


$^{13}$**C NMR** (151 MHz, DMSO-$d_6$)

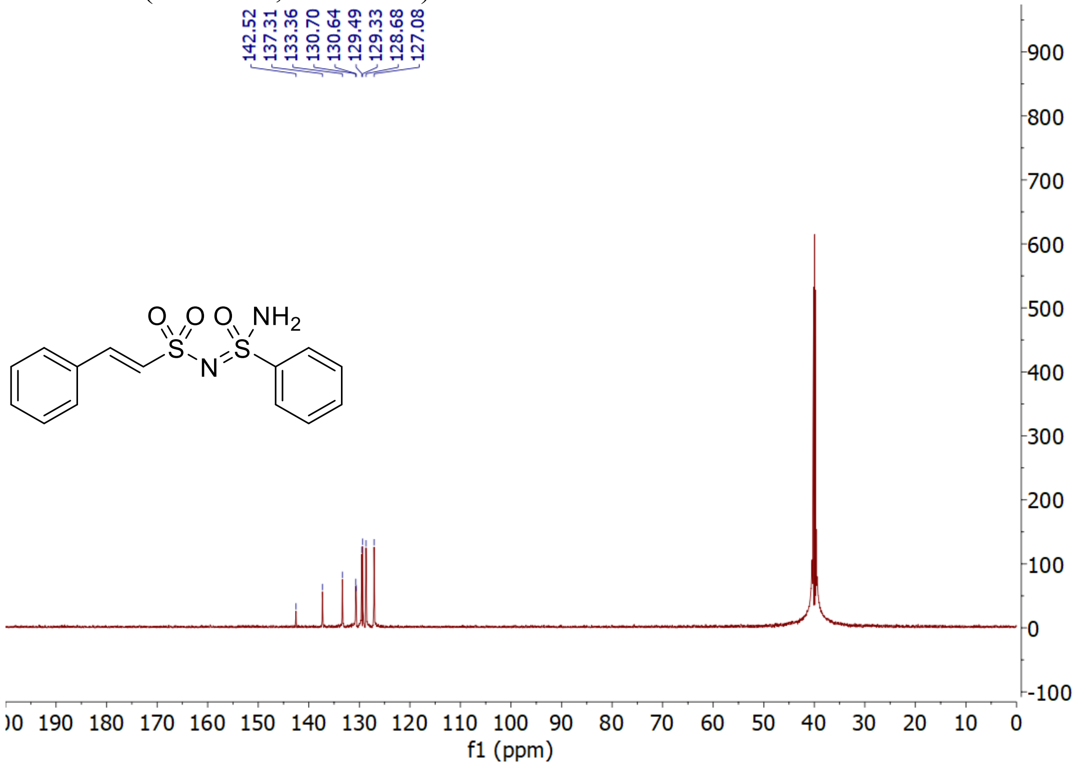

**$N$-(Amino(oxo)(phenyl)-λ$^6$-sulfaneylidene)methanesulfonamide (12l)**

$^1$**H NMR** (600 MHz, DMSO-$d_6$)

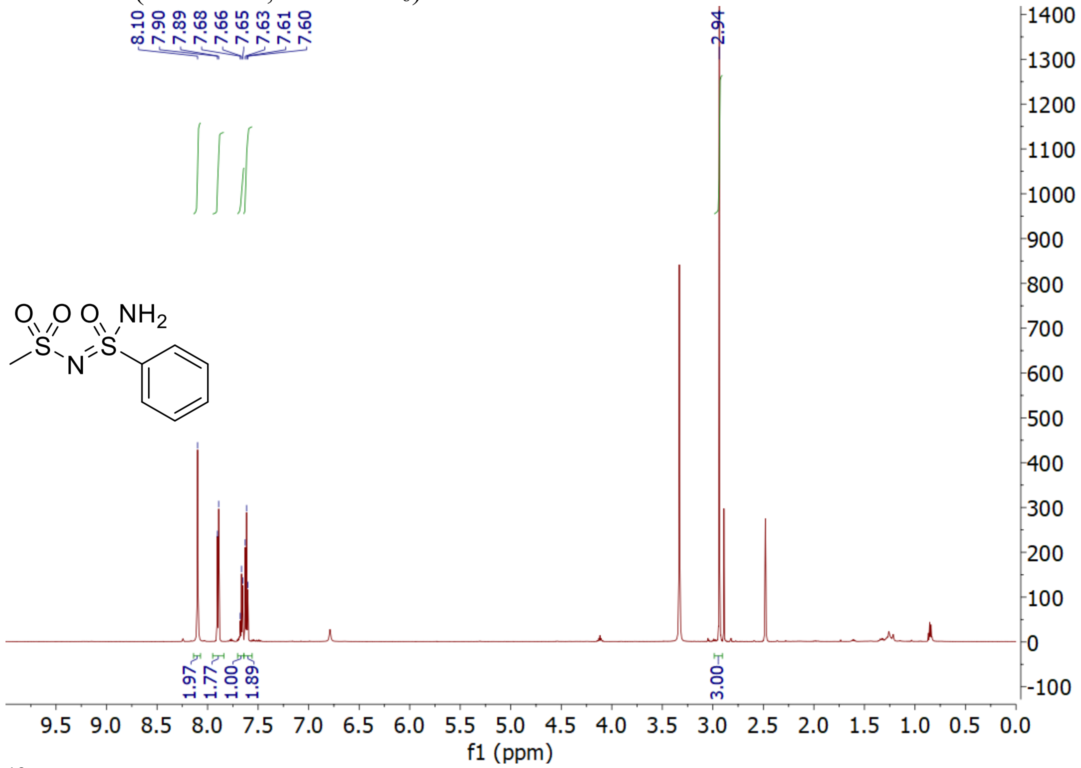


$^{13}$**C NMR** (151 MHz, DMSO-$d_6$)

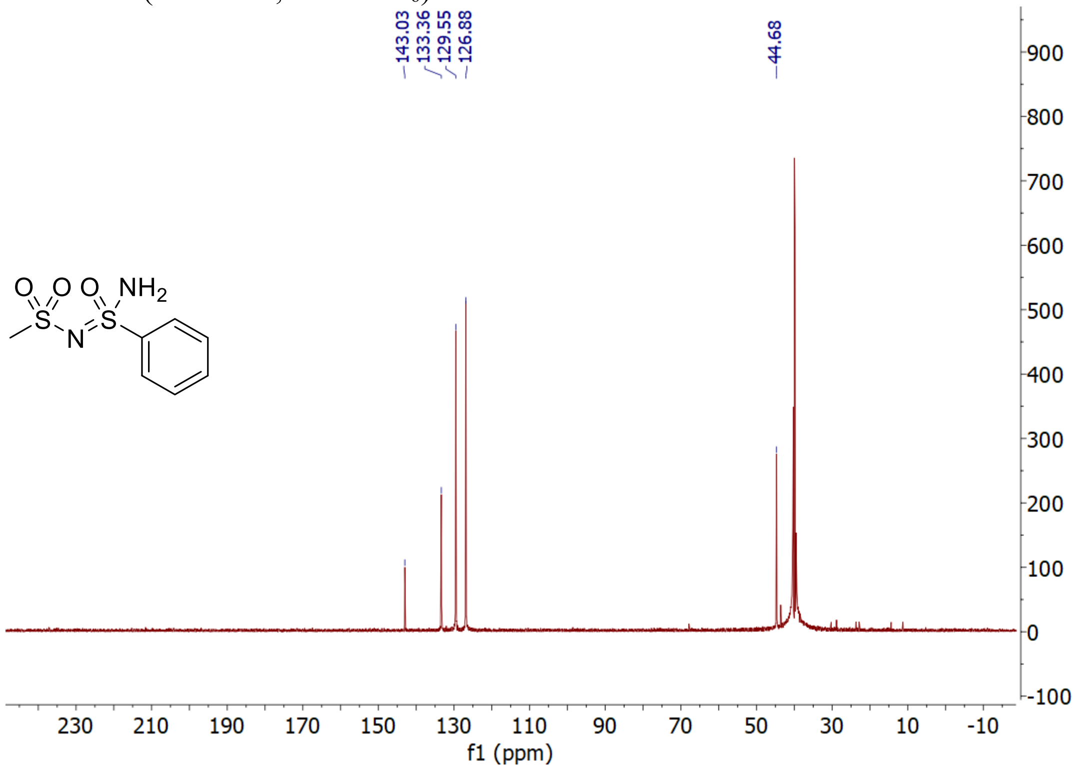

***N*-(Amino(oxo)(phenyl)- λ[6]-sulfaneylidene)-1-phenylmethanesulfonamide (12m)**

[1]**H NMR** (151 MHz Chloroform-*d*)

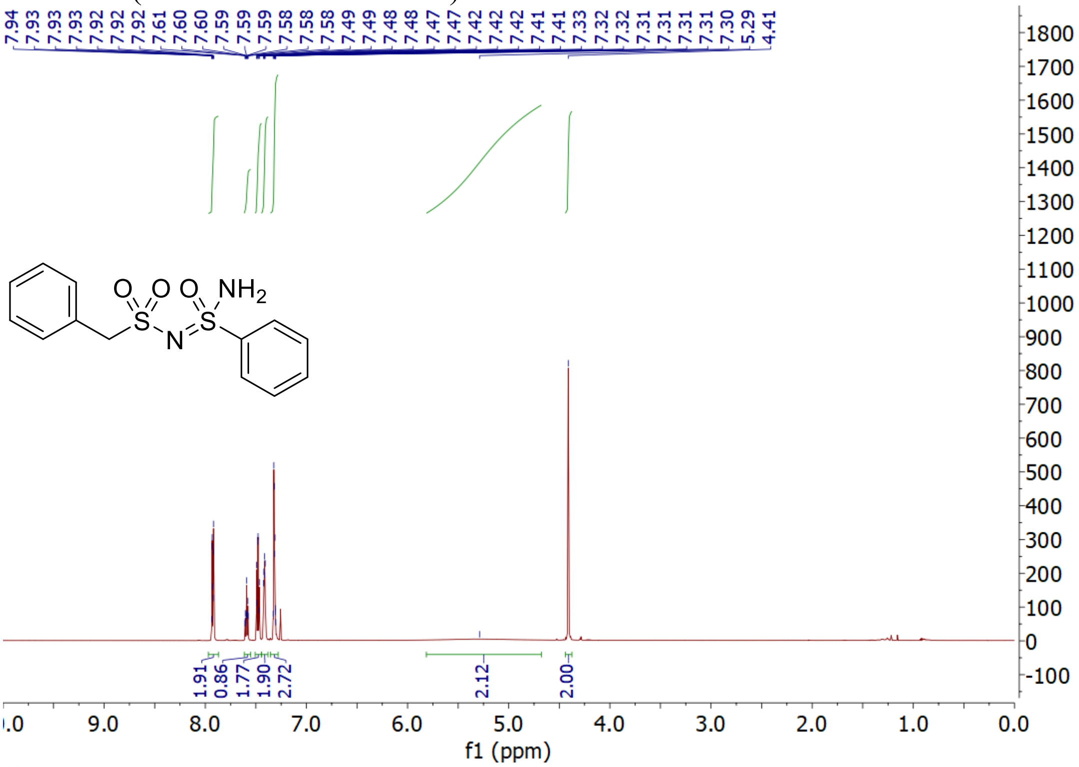


[13]**C NMR** (151 MHz Chloroform-*d*)

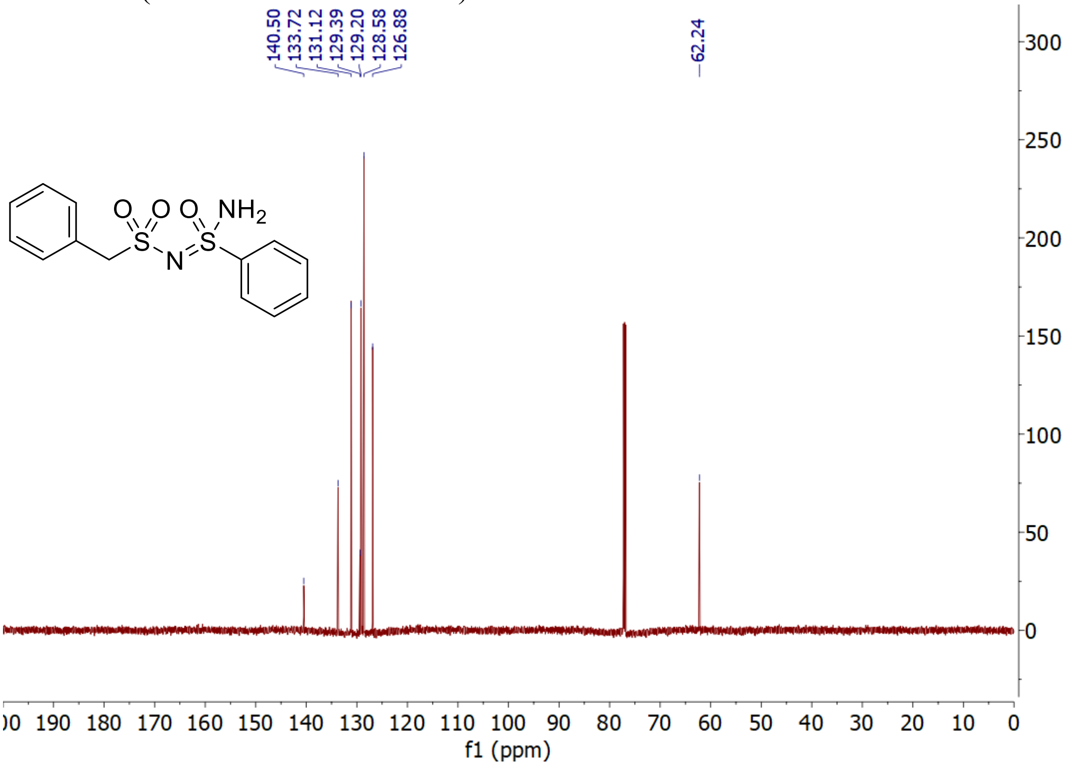

**$N$-(Amino(oxo)($p$-tolyl)- $\lambda^6$-sulfaneylidene)-4-methylbenzenesulfonamide (12n)**

**$^1$H NMR** (600 MHz, DMSO-$d_6$)

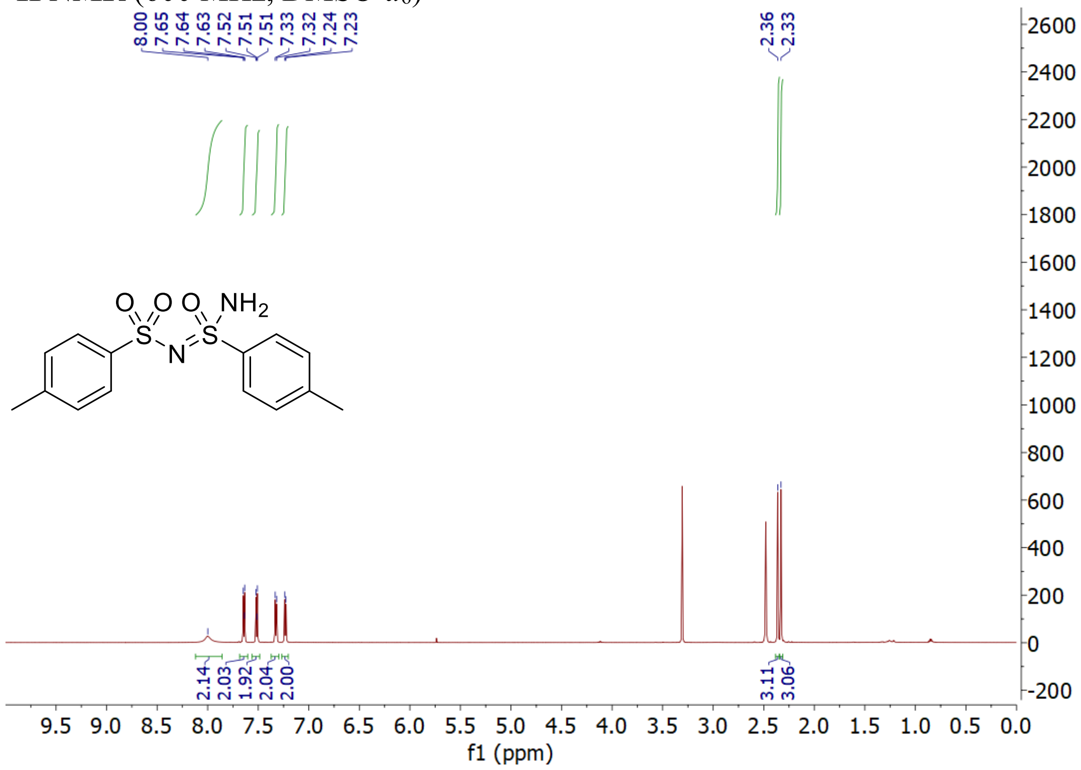


**$^{13}$C NMR** (151 MHz, DMSO-$d_6$)

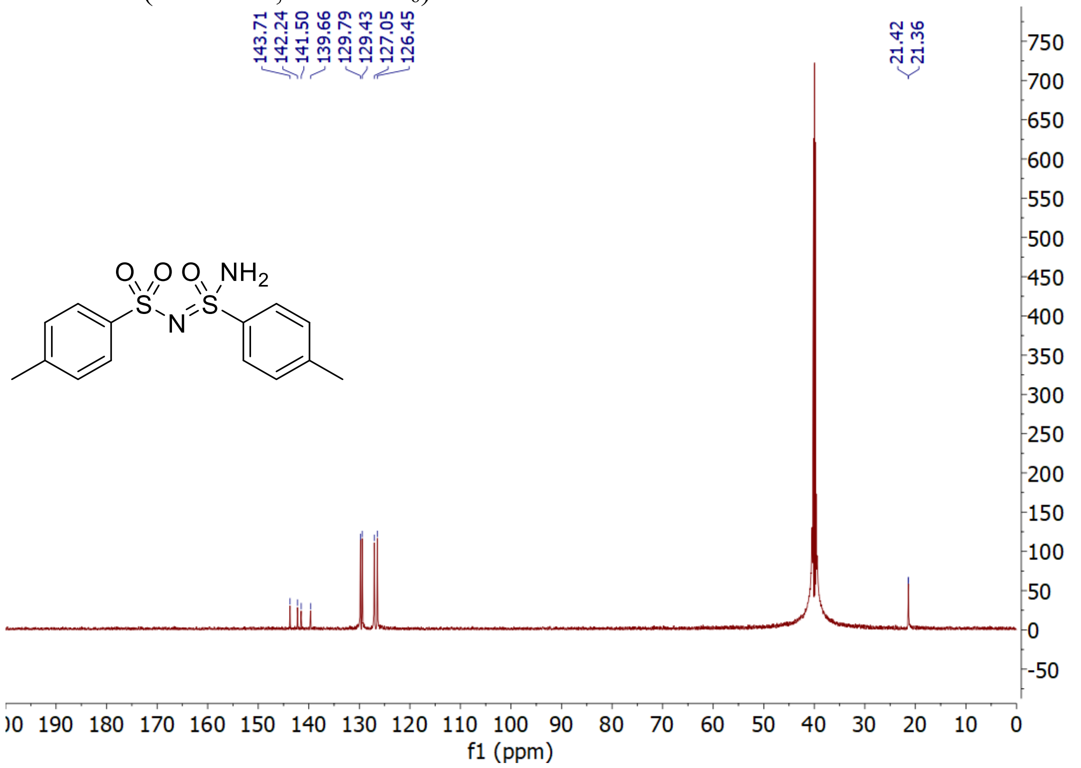

**N-(Amino(4-fluorophenyl)(oxo)- λ⁶-sulfaneylidene)-4-methylbenzenesulfonamide (12o)**

**¹H NMR** (600 MHz, DMSO-$d_6$)

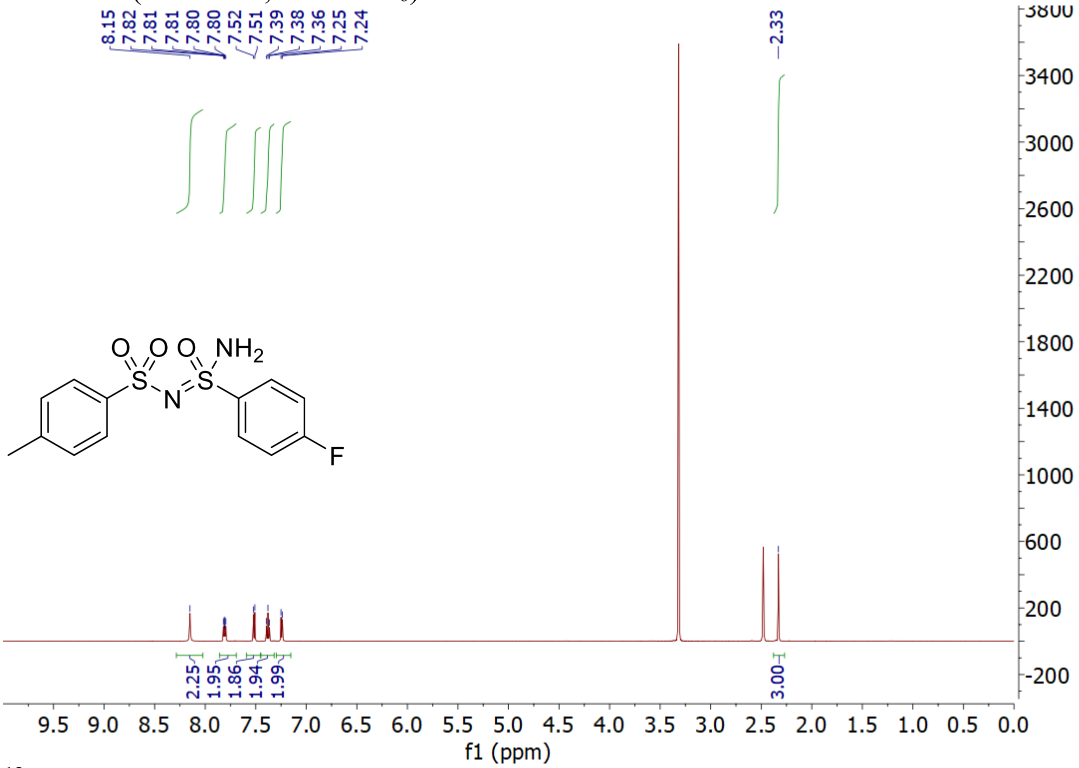


**¹³C NMR** (151 MHz, DMSO-$d_6$)

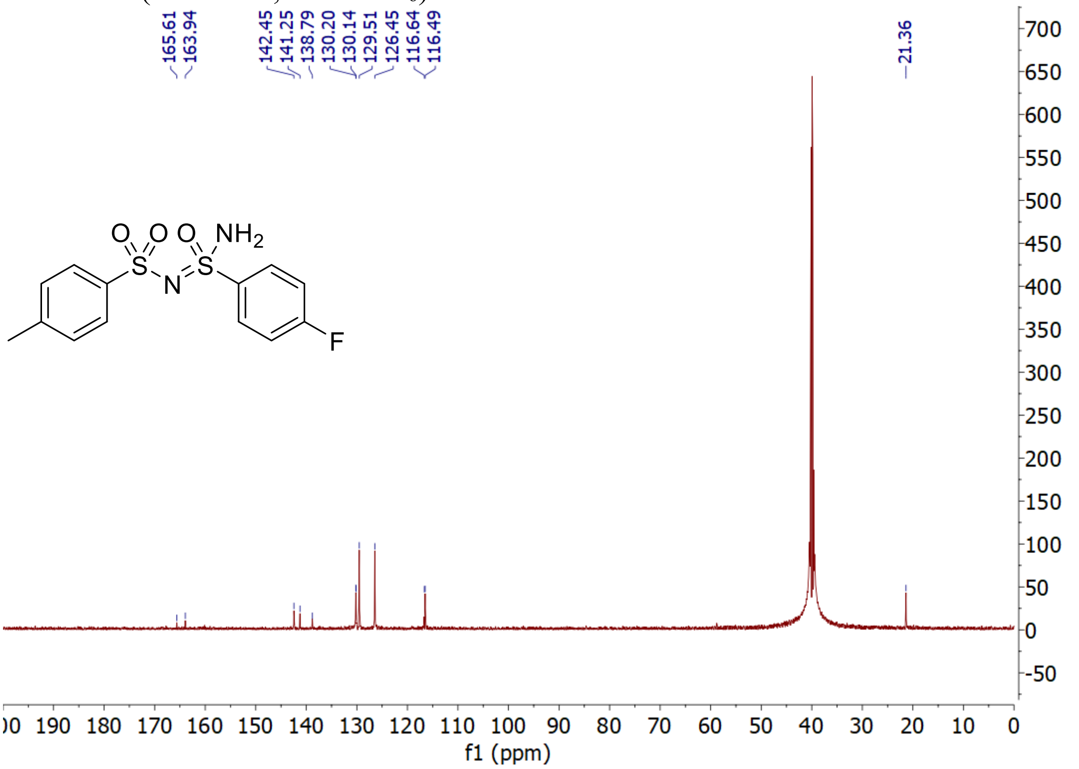

**$^{19}$F NMR** (564 MHz, DMSO-$d_6$)

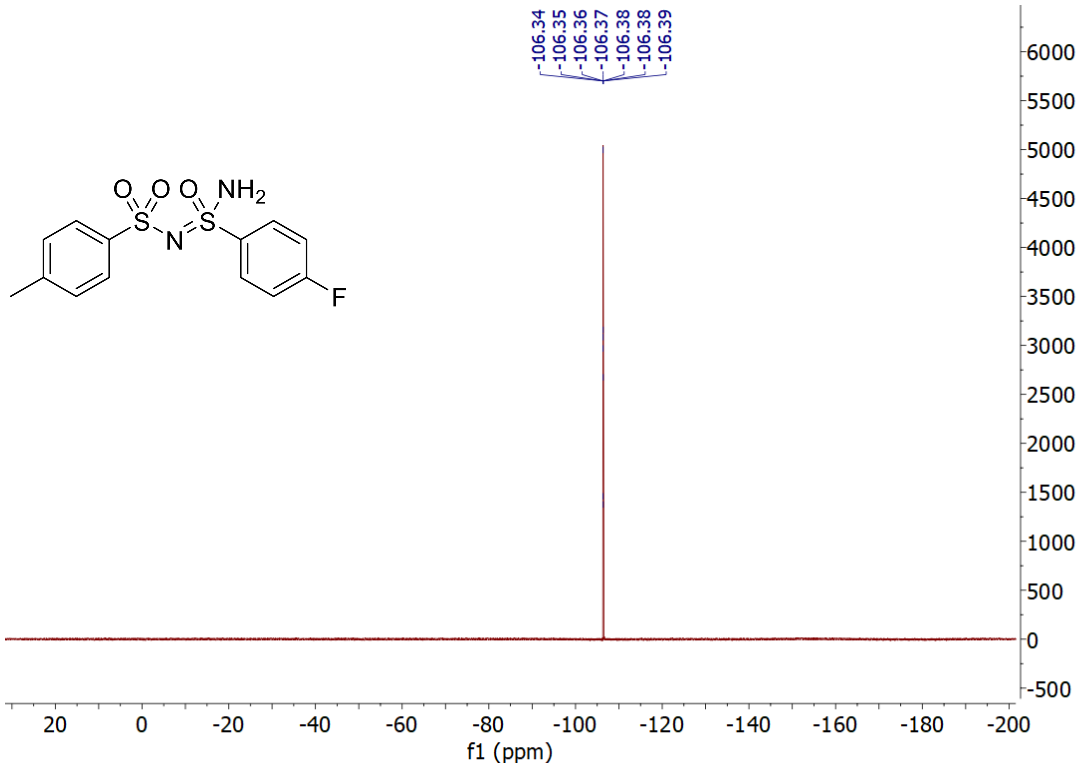

**N-(Amino(4-chlorophenyl)(oxo)- λ6-sulfaneylidene)-4-methylbenzenesulfonamide (12p)**

**$^{1}$H NMR** (600 MHz, Chloroform-*d*)

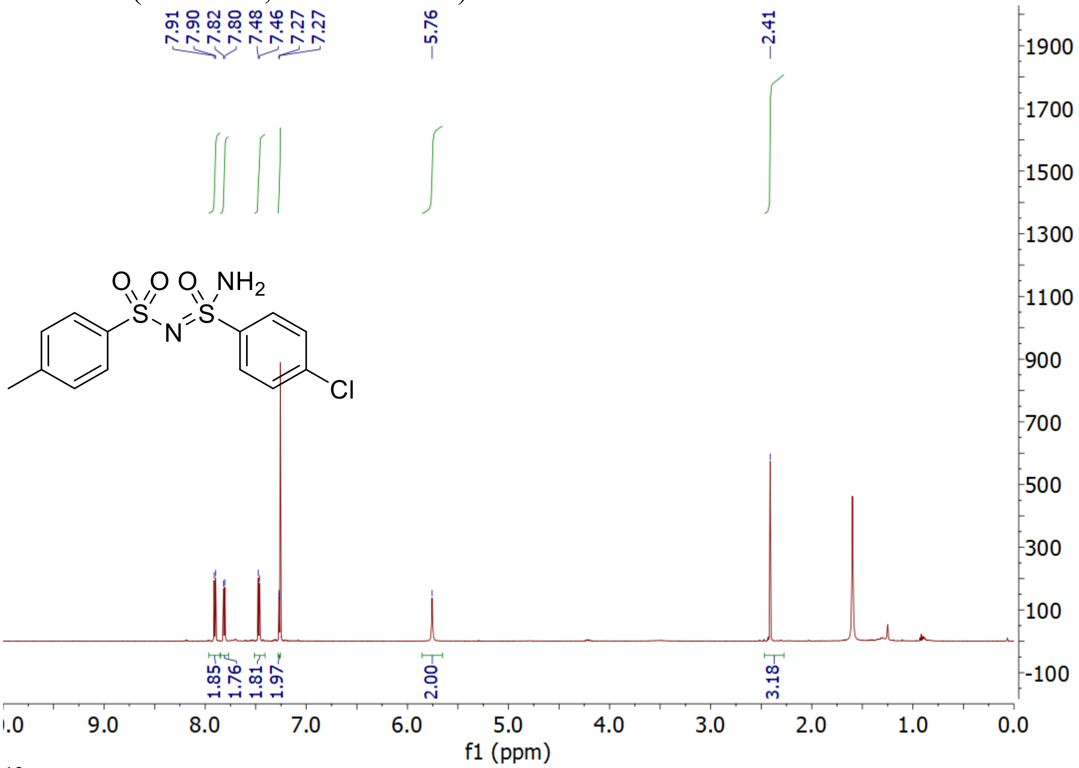


**$^{13}$C NMR** (151 MHz, Chloroform-*d*)

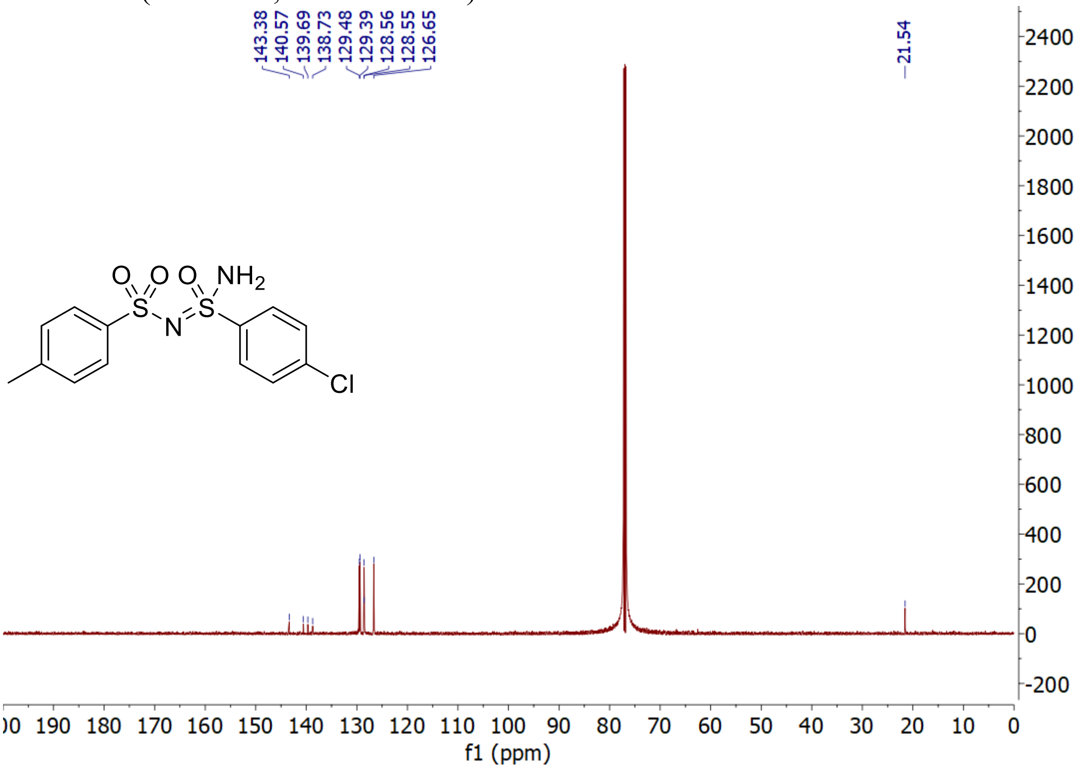

***N*-(Amino(4-bromophenyl)(oxo)- λ⁶-sulfaneylidene)-4-methylbenzenesulfonamide (12q)**

$^{1}$**H NMR** (600 MHz, Chloroform-*d*)

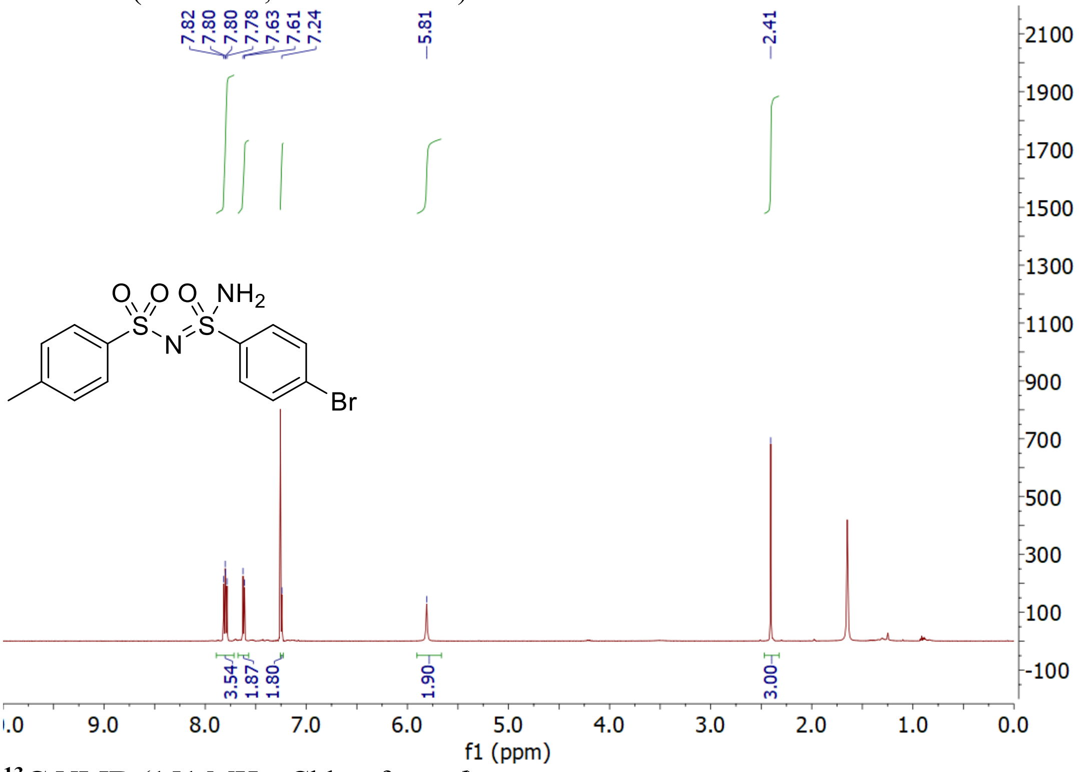


$^{13}$**C NMR** (151 MHz, Chloroform-*d*)

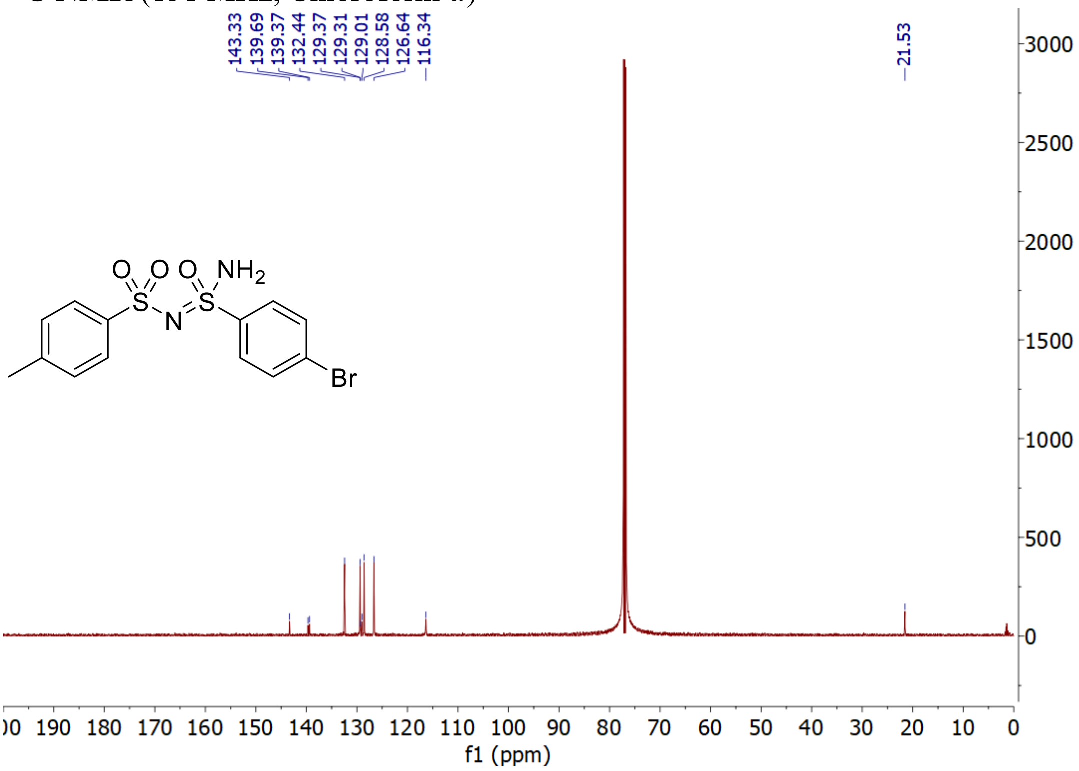

***N*-(Amino(3-methoxyphenyl)(oxo)-λ⁶-sulfaneylidene)-4-methylbenzenesulfonamide (12r)**

**$^1$H NMR** (600 MHz, DMSO-$d_6$)

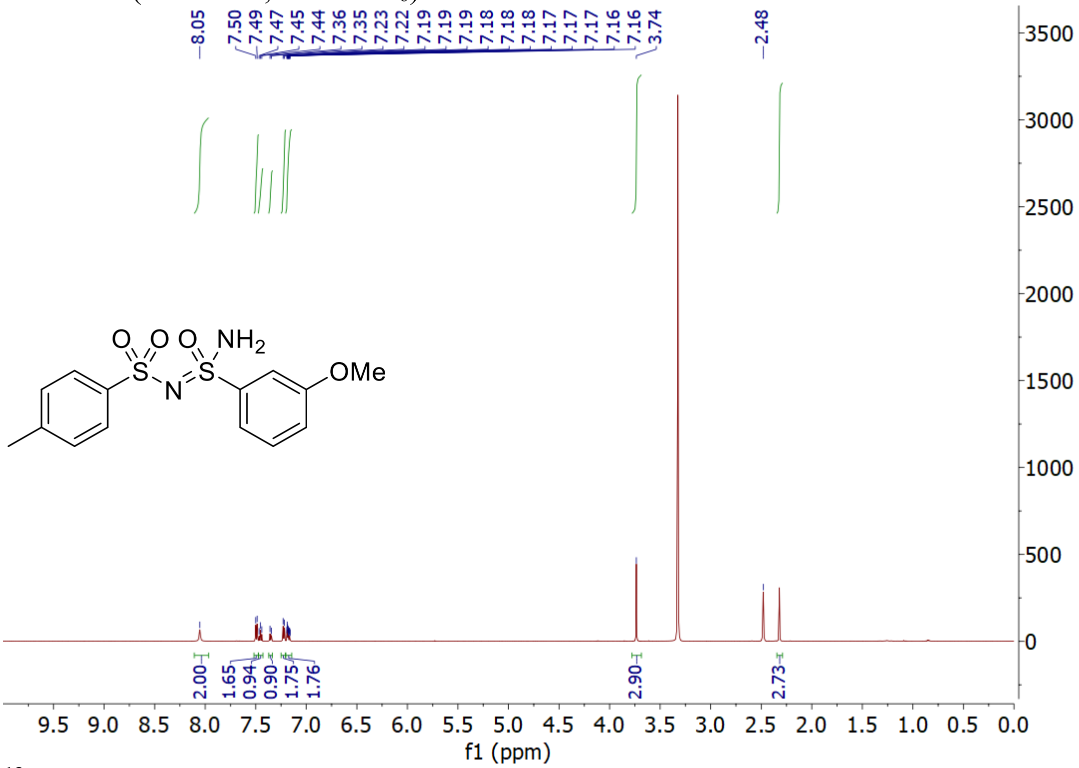


**$^{13}$C NMR** (151 MHz, DMSO-$d_6$)

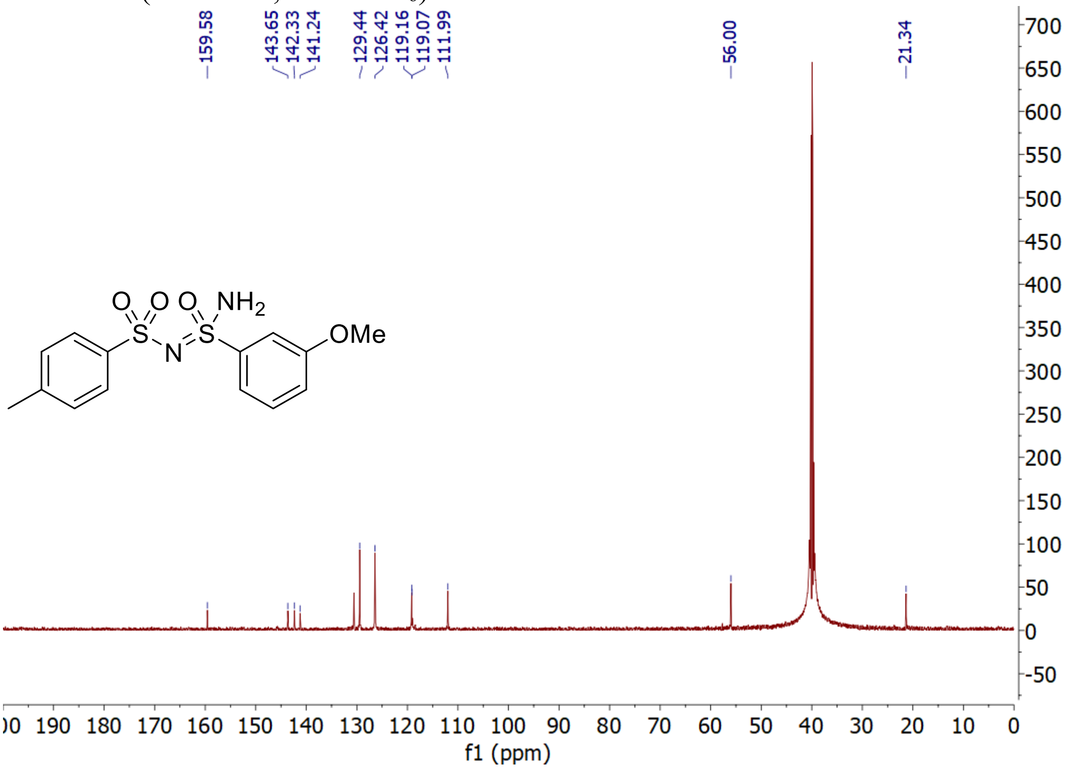

***N*-(Amino(2-methoxyphenyl)(oxo)-λ⁶-sulfaneylidene)-4-methylbenzenesulfonamide (12s)**

$^{1}$**H NMR** (600 MHz, Chloroform-*d*)

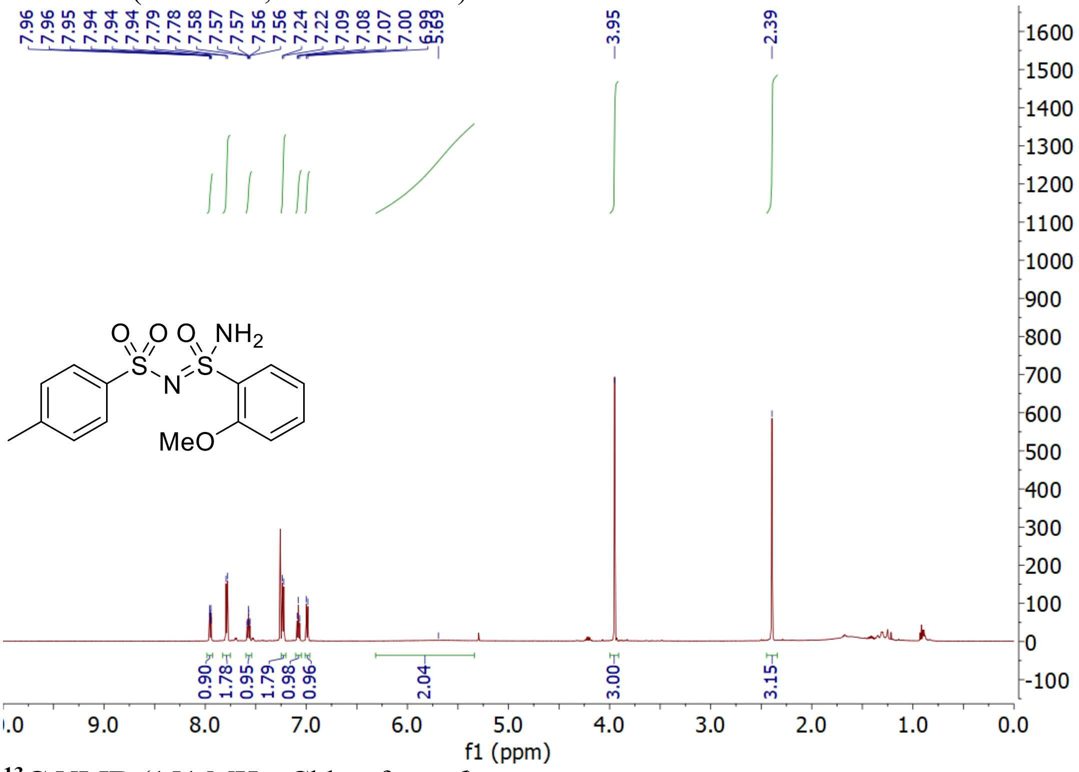

$^{13}$**C NMR** (151 MHz, Chloroform-*d*)

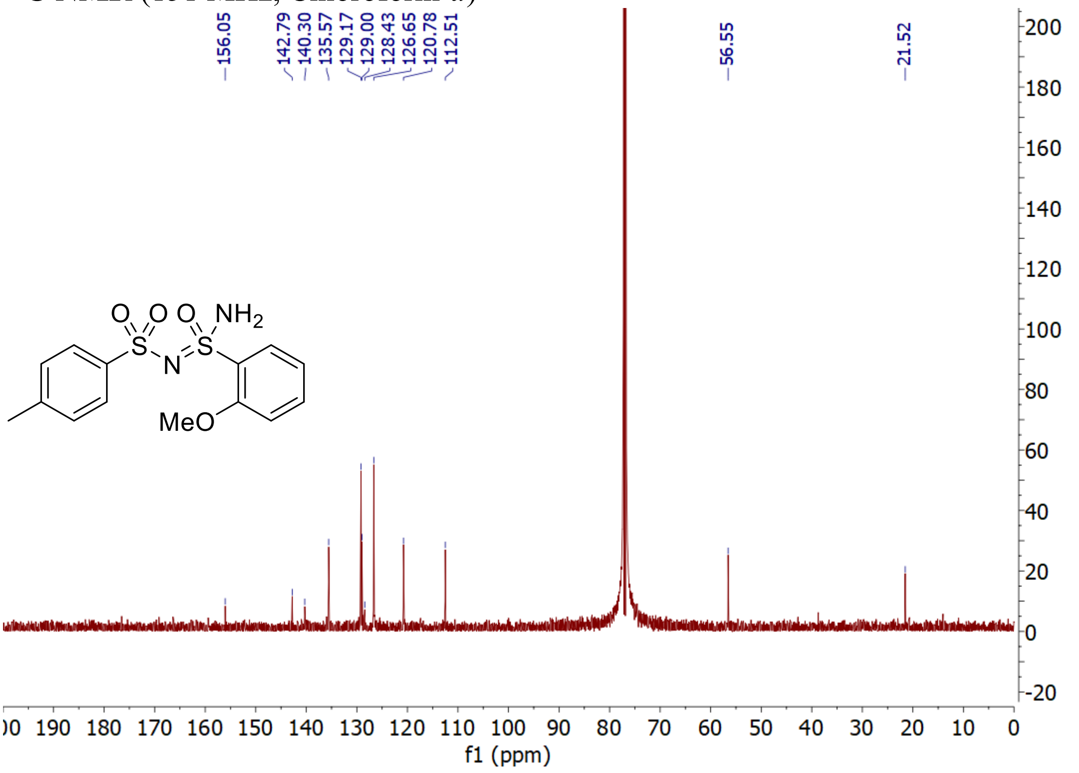

***N*-(Amino(naphthalen-2-yl)(oxo)- λ^6^-sulfaneylidene)-4-methylbenzenesulfonamide (12t)**

**$^1$H NMR** (600 MHz, Chloroform-*d*)

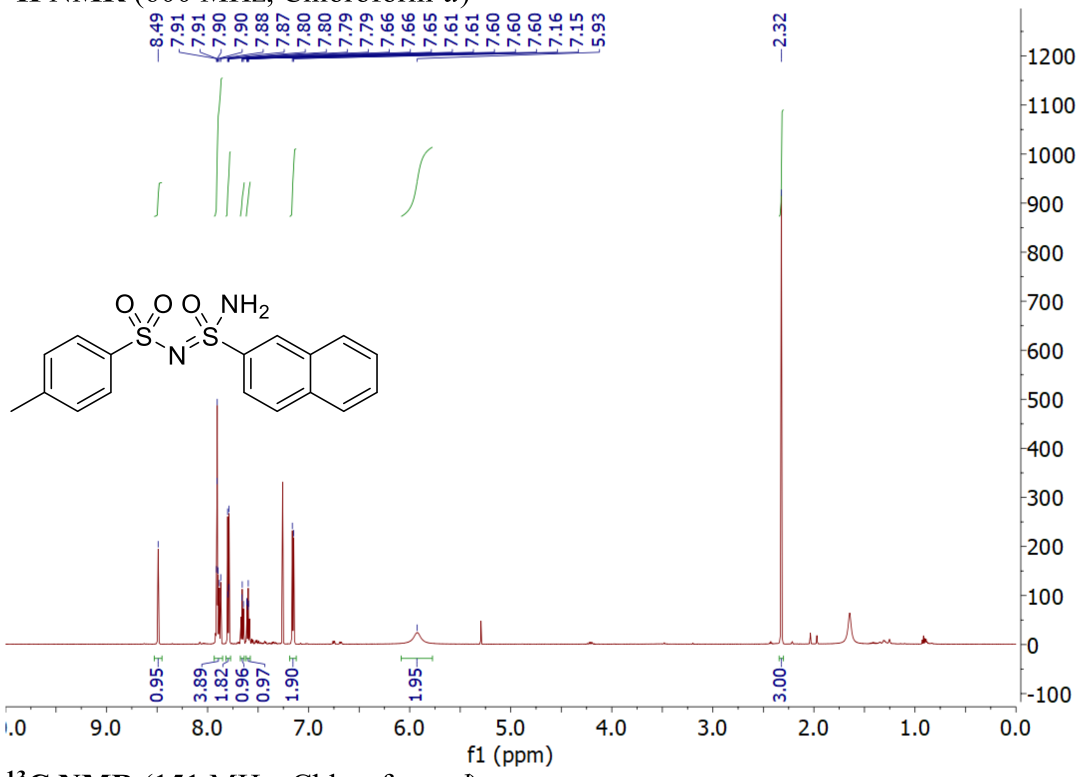


**$^{13}$C NMR** (151 MHz, Chloroform-*d*)

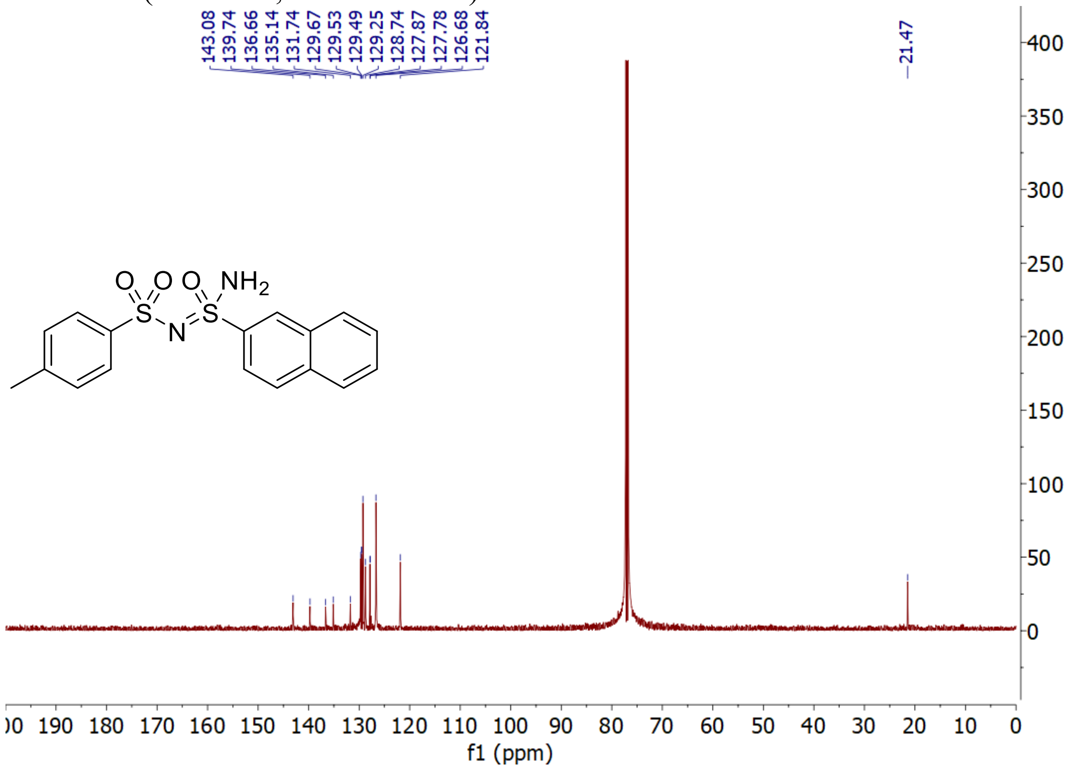

**N-(Amino(benzyl)(oxo)- λ[6]-sulfaneylidene)-4-methylbenzenesulfonamide (12u)**

**$^{1}$H NMR** (600 MHz, DMSO-$d_6$)

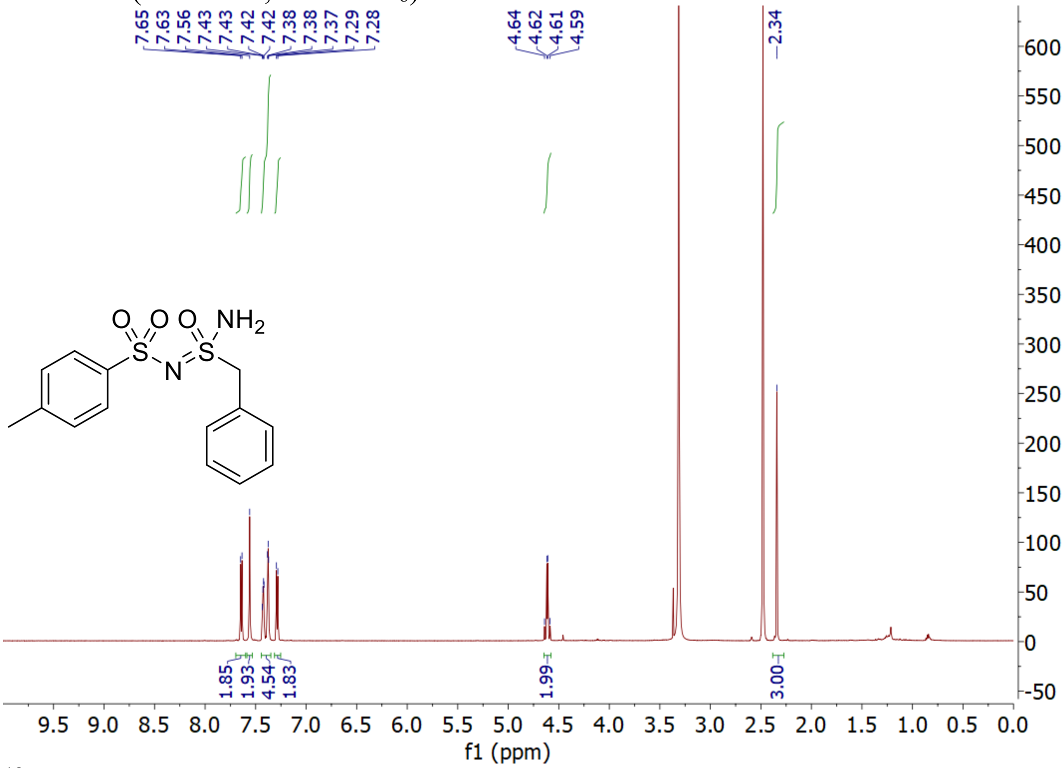

**$^{13}$C NMR** (151 MHz, DMSO-$d_6$)

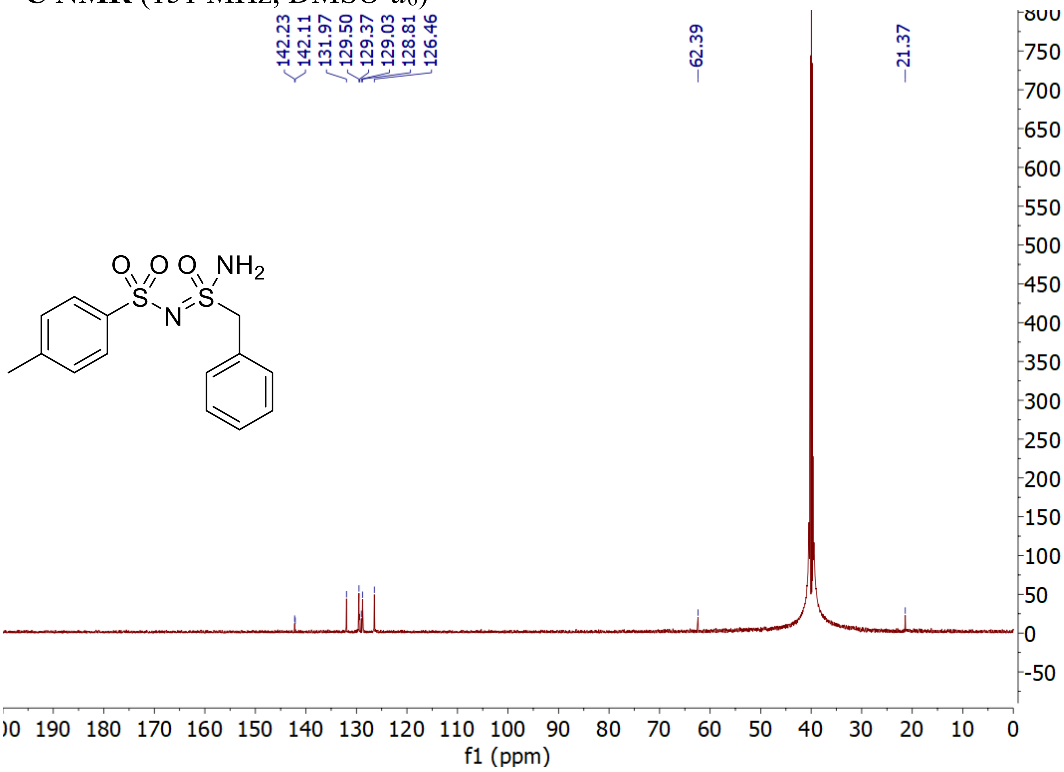

***N*-(Amino(oxo)(phenethyl)- λ⁶-sulfaneylidene)-4-methylbenzenesulfonamide (12v)**

**¹H NMR** (600 MHz, Chloroform-*d*)

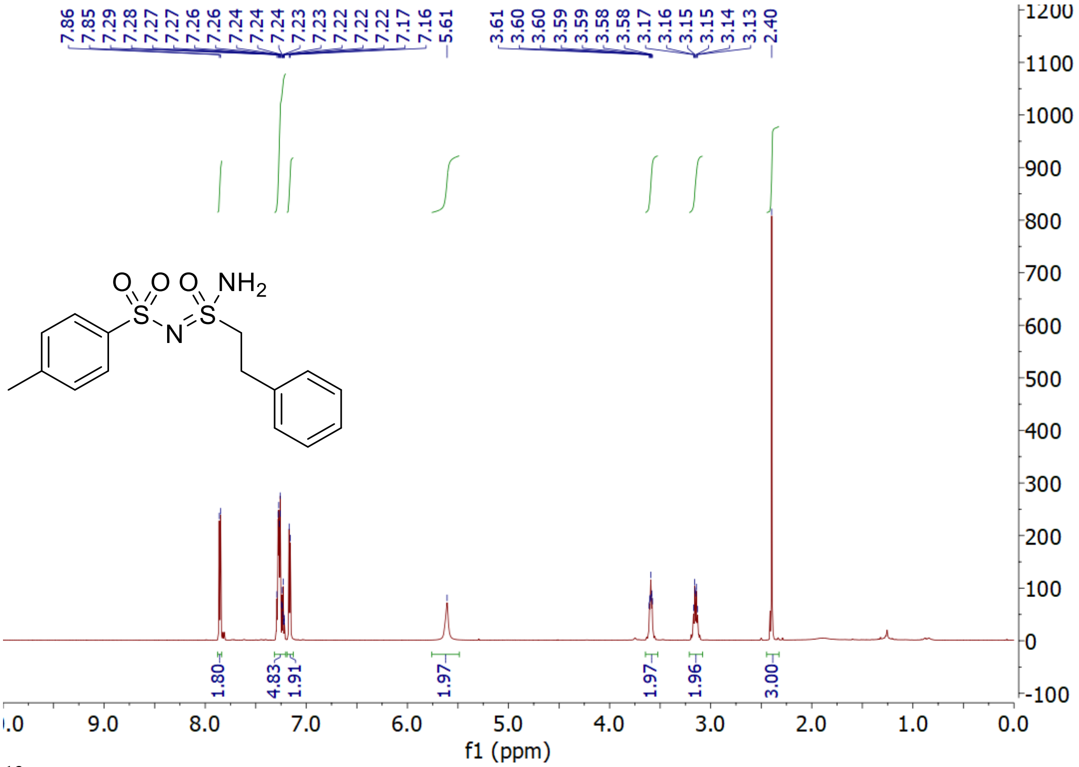


**¹³C NMR** (151 MHz, Chloroform-*d*):

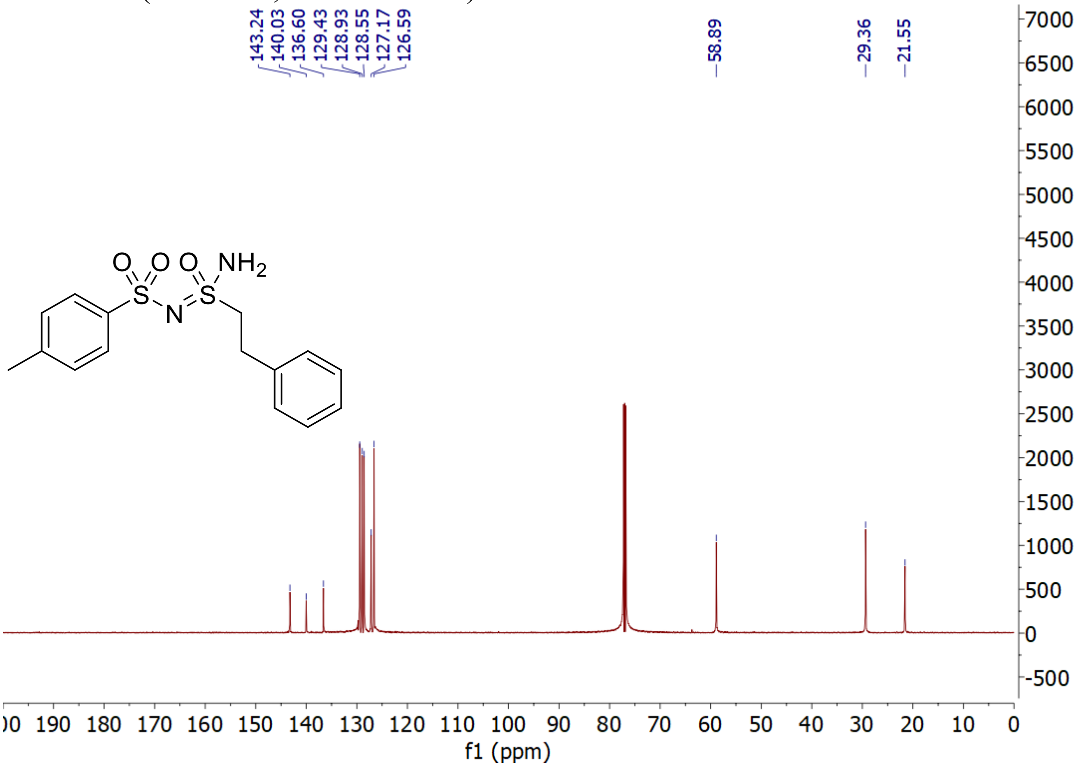

**(*R*)-*N*-(Amino(tert-butyl)(oxo)-l6-sulfaneylidene)-4-methylbenzenesulfonamide (12w)**

**$^{1}$H NMR**(600 MHz, Chloroform-*d*)

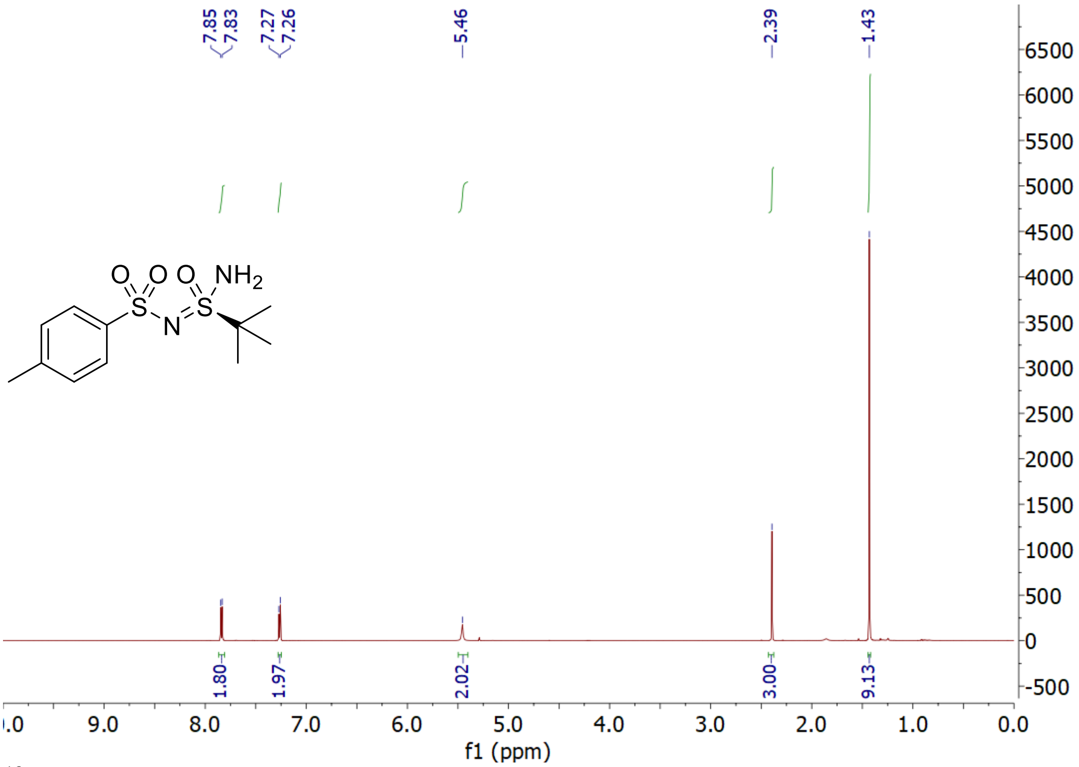


**$^{13}$C NMR** (151 MHz, Chloroform-*d*)

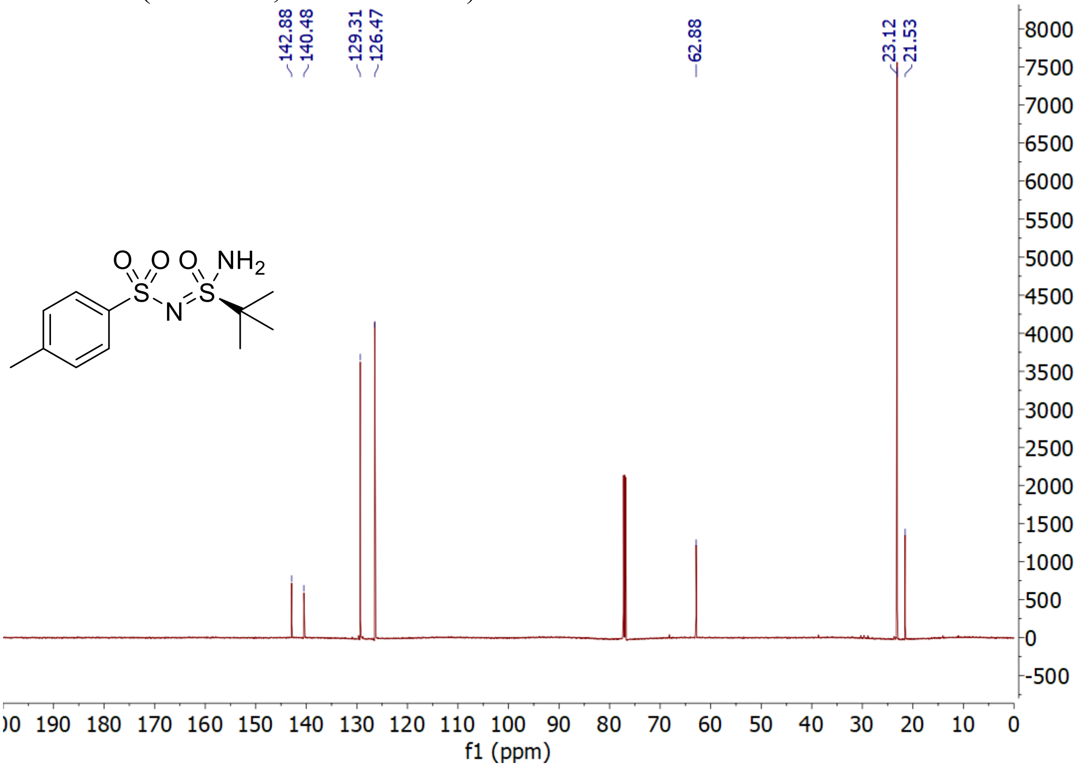

***N*-((Isopropylamino)(oxo)(*p*-tolyl)-λ$^6$-sulfaneylidene)-4-methylbenzenesulfonamide (12x)**

$^1$**H NMR** (600 MHz, Chloroform-*d*)

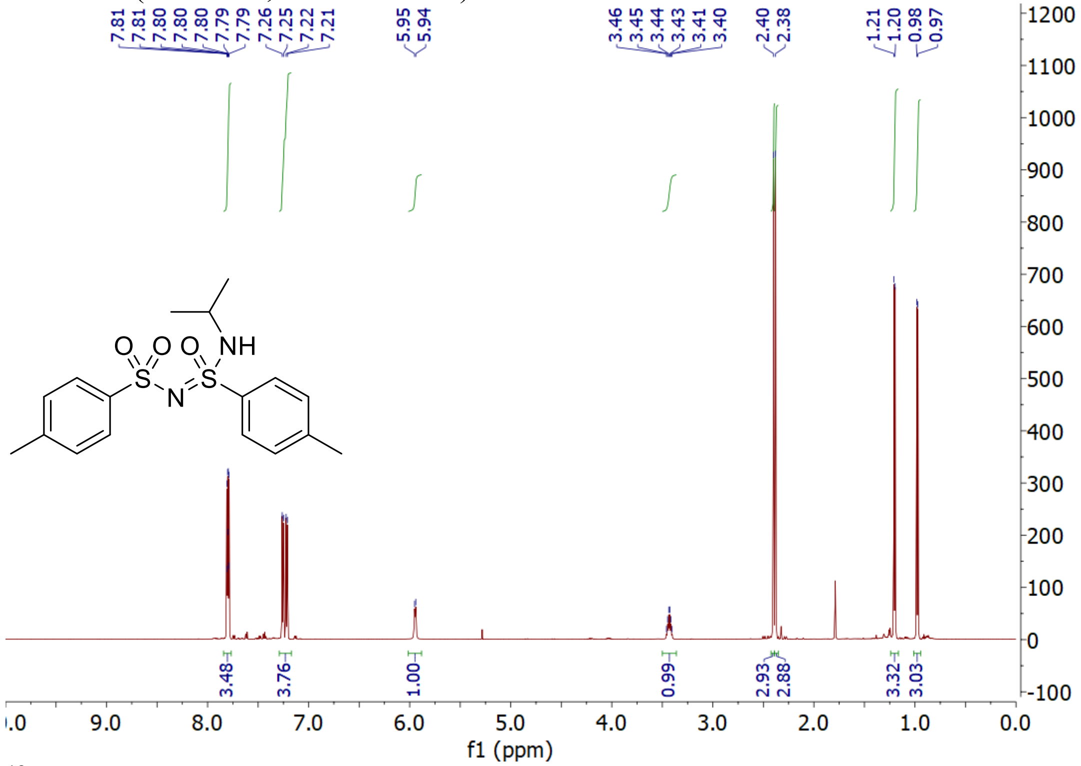


$^{13}$**C NMR** (600 MHz, Chloroform-*d*)

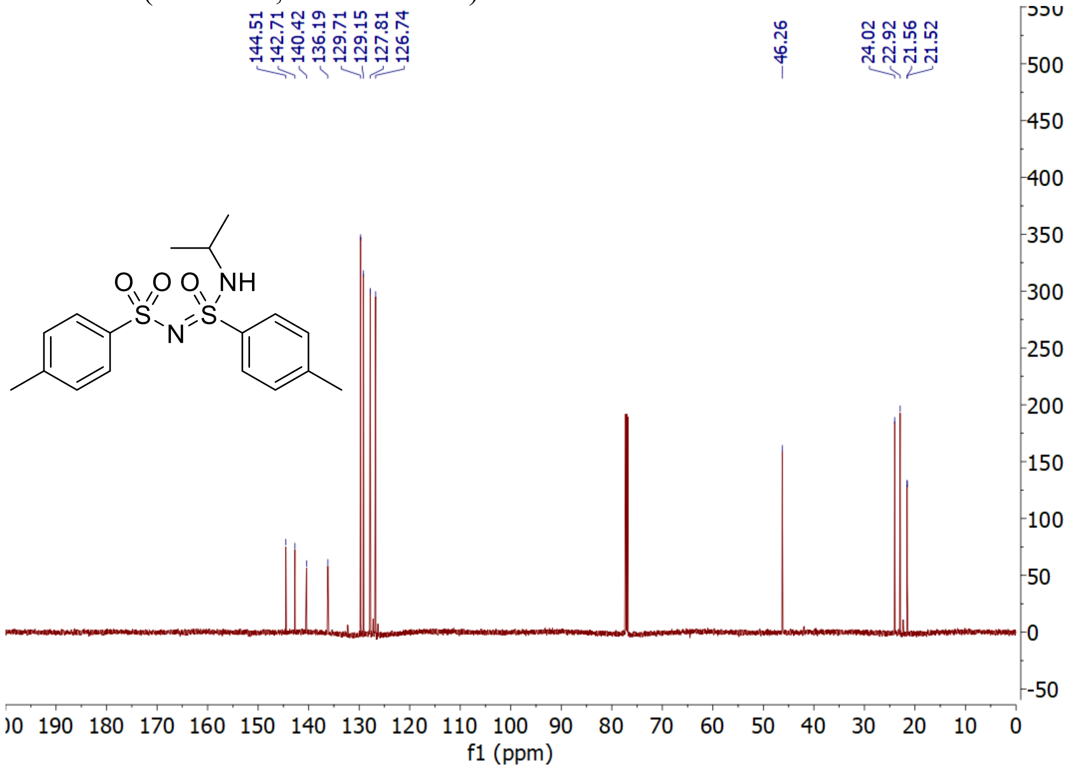

**_N_-((Cyclohexylamino)(oxo)(_p_-tolyl)-λ$^6$-sulfaneylidene)-4-methylbenzenesulfonamide (12y)**

$^1$**H NMR** (600 MHz, Chloroform-_d_)

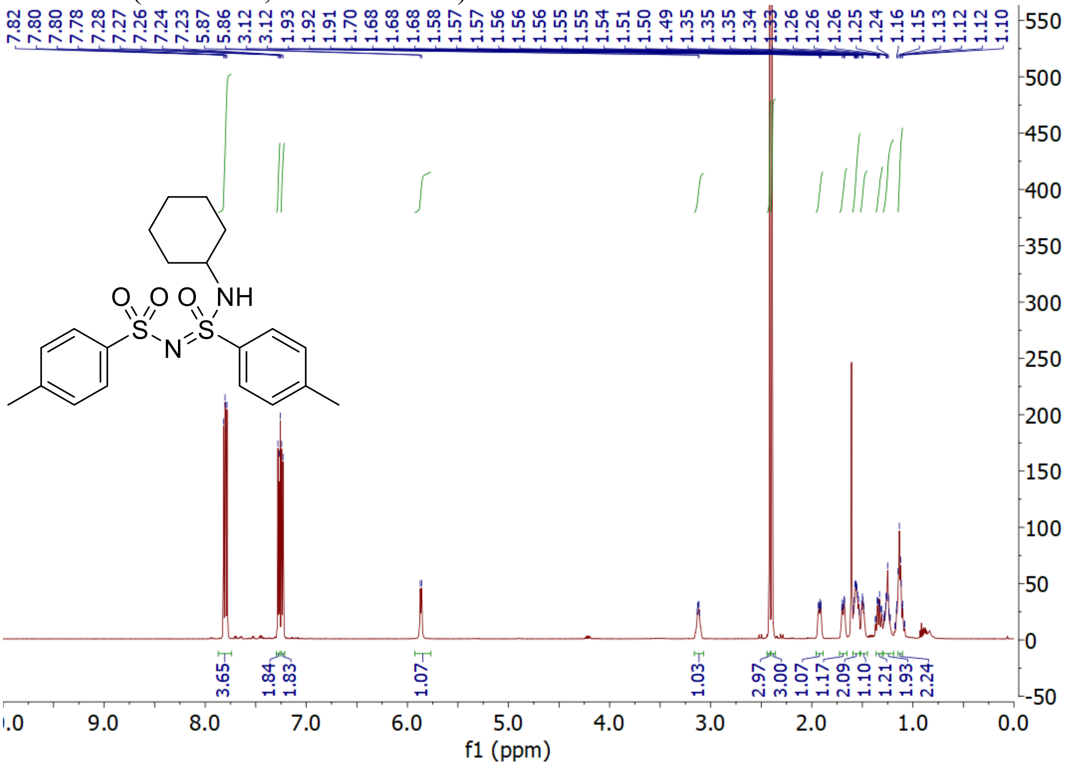


$^{13}$**C NMR** (151 MHz, Chloroform-_d_)

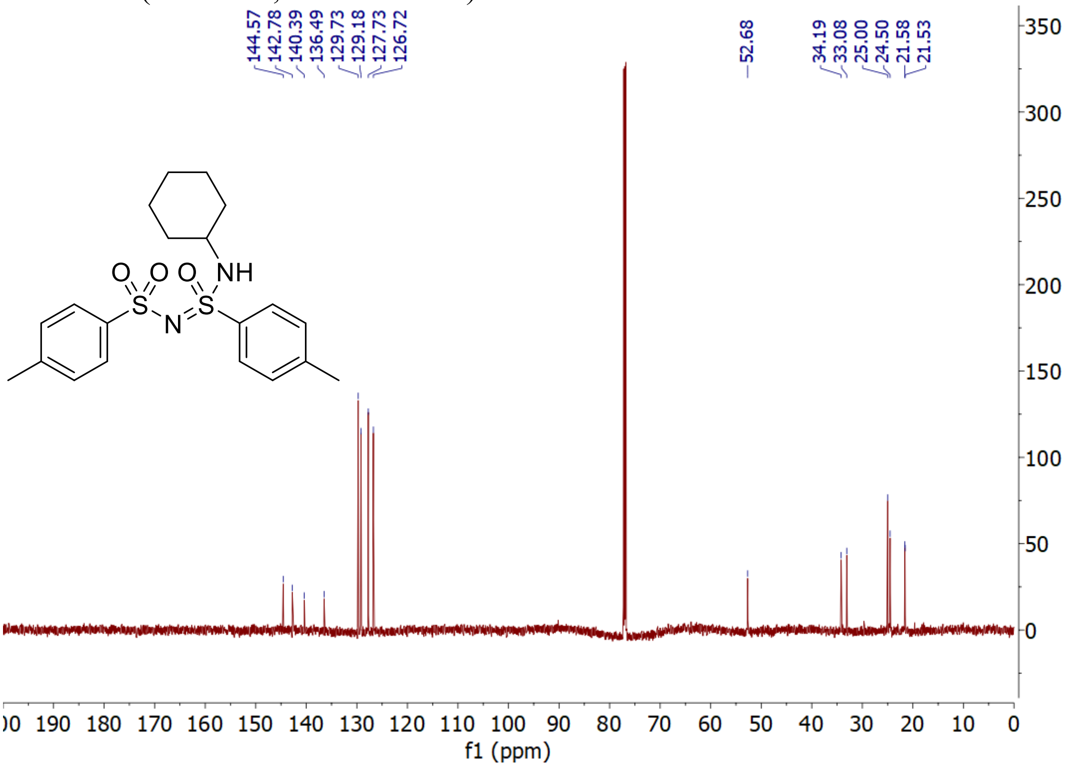

***N*-((Benzylamino)(oxo)(*p*-tolyl)-λ⁶-sulfaneylidene)-4-methylbenzenesulfonamide (12z)**

**¹H NMR** (600 MHz, Chloroform-*d*)

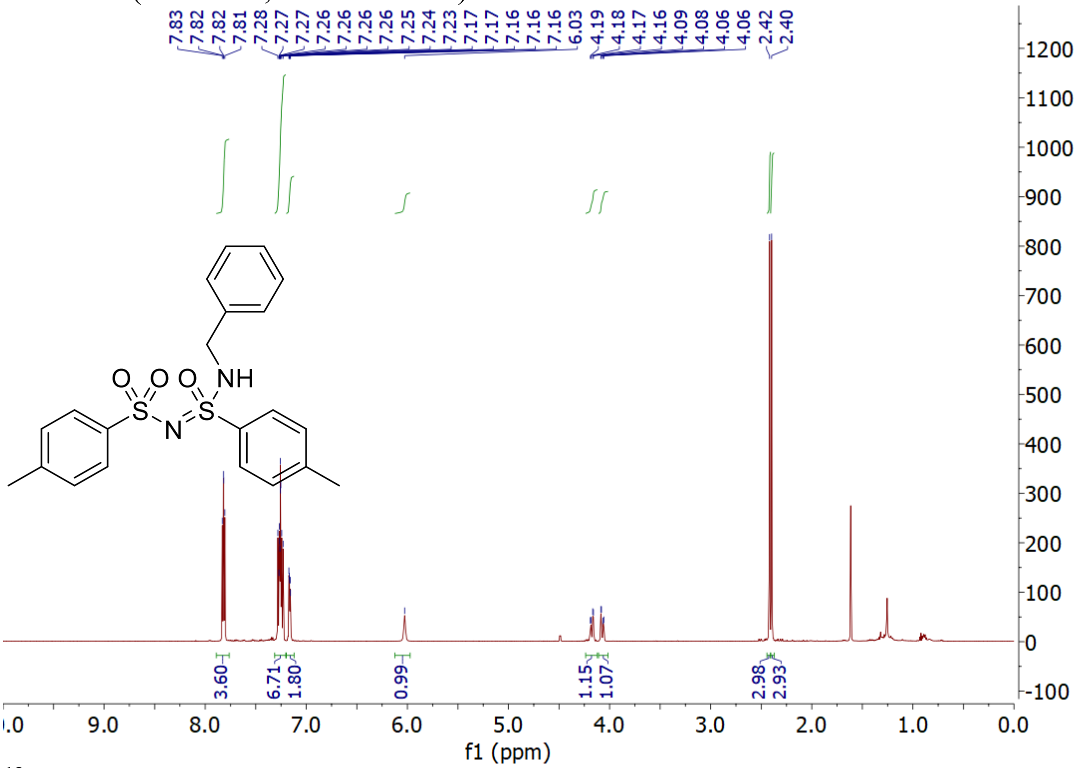


**¹³C NMR** (151 MHz, Chloroform-*d*)

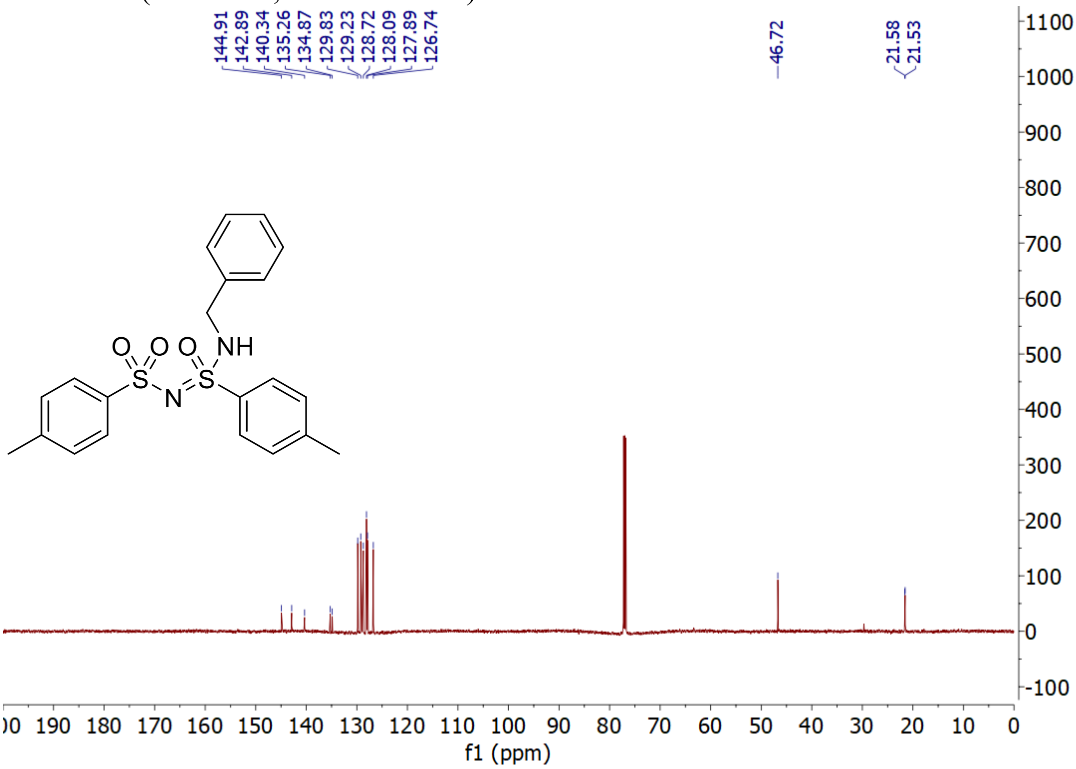

**4-Methyl-*N*-(oxo(phenethylamino)(*p*-tolyl)-λ$^6$-sulfaneylidene) benzenesulfonamide (12aa)**

**$^1$H NMR** (600 MHz, Chloroform-*d*)

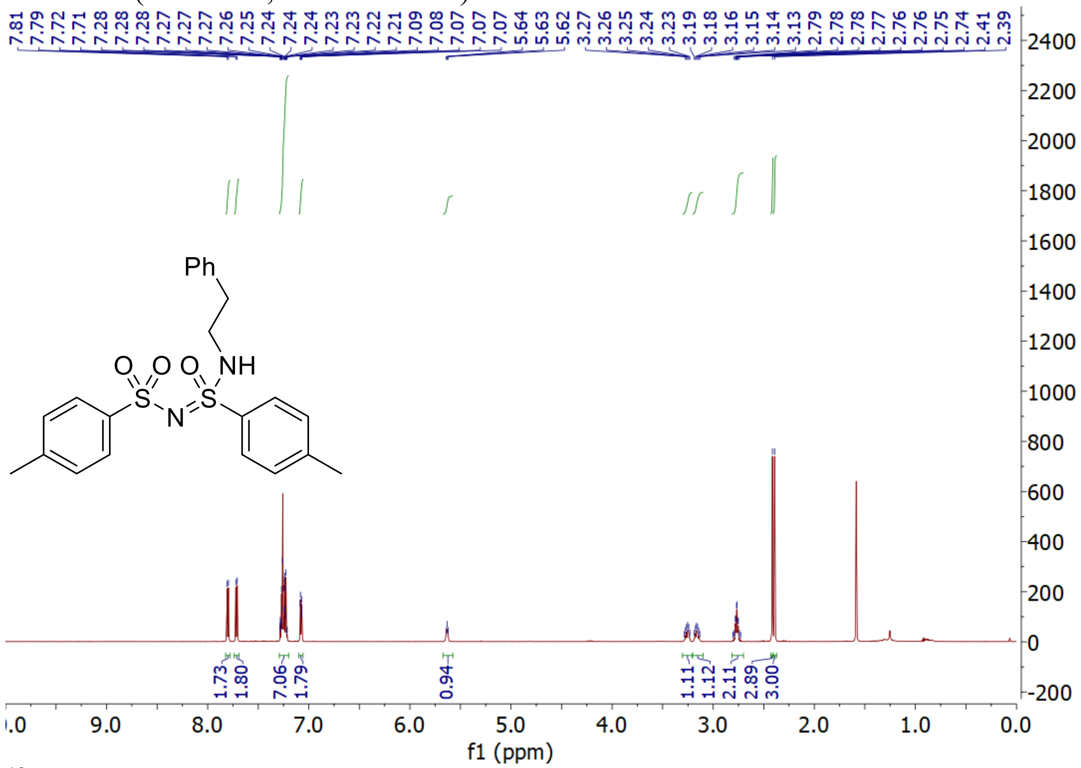

**$^{13}$C NMR** (151 MHz, Chloroform-*d*)

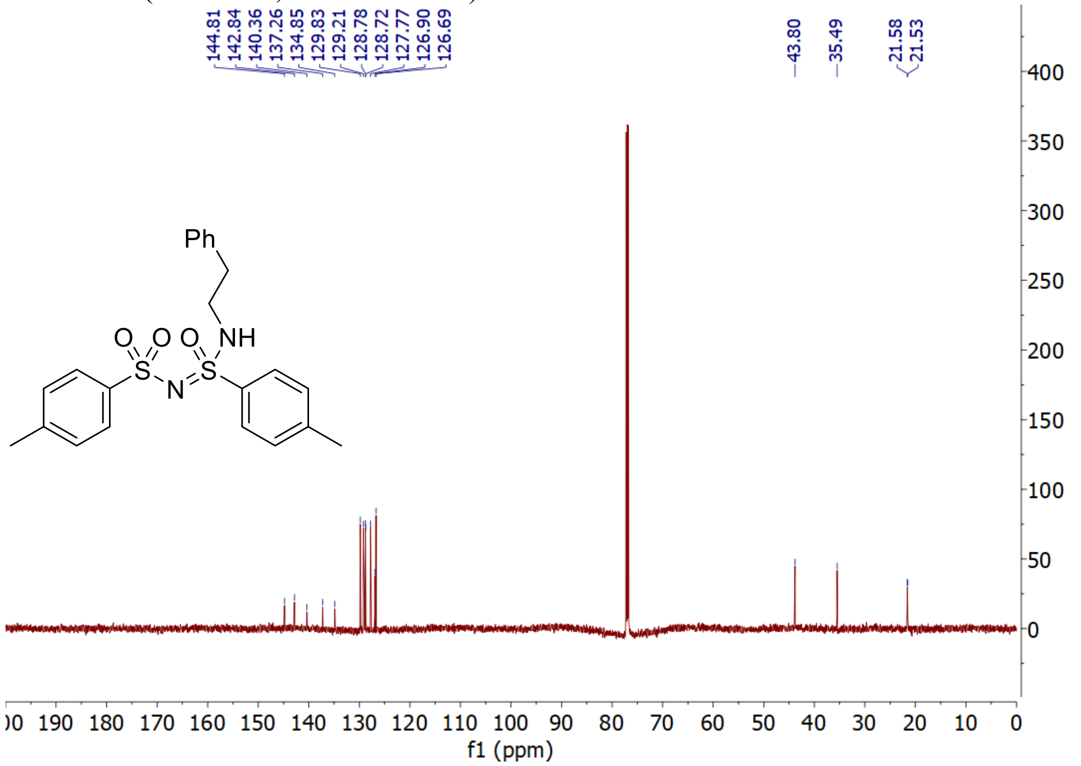

**4-Methyl-*N*-(oxo(*p*-tolyl)((2,2,2-trifluoroethyl)amino)-λ$^6$-sulfaneylidene)benzenesulfonamide (12ab)**

$^1$**H NMR** (600 MHz, Chloroform-*d*)

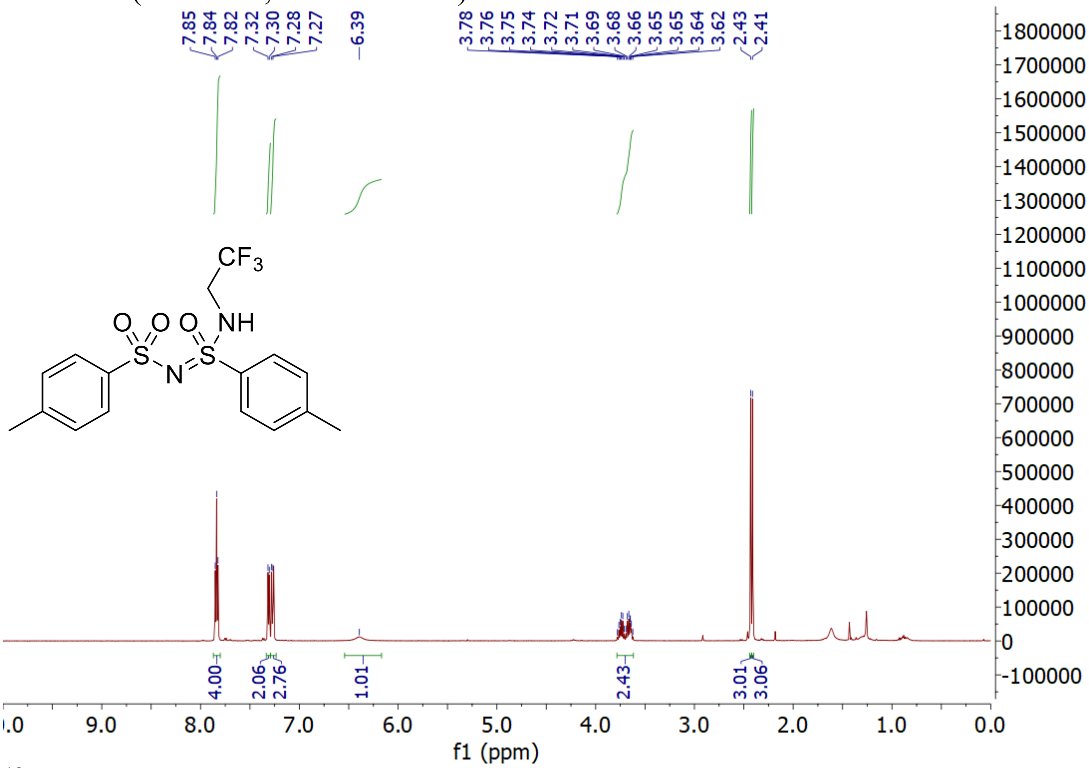


$^{13}$**C NMR** (151 MHz, Chloroform-*d*)

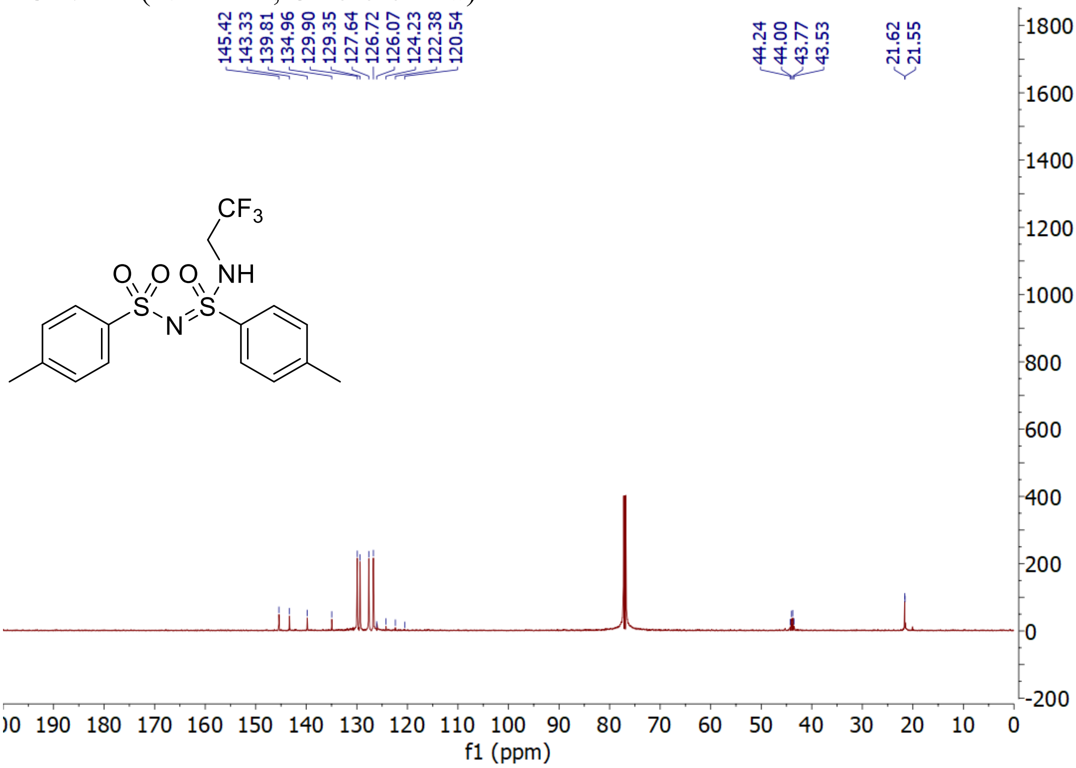

**$^{19}$F NMR** (565 MHz, Chloroform-*d*)

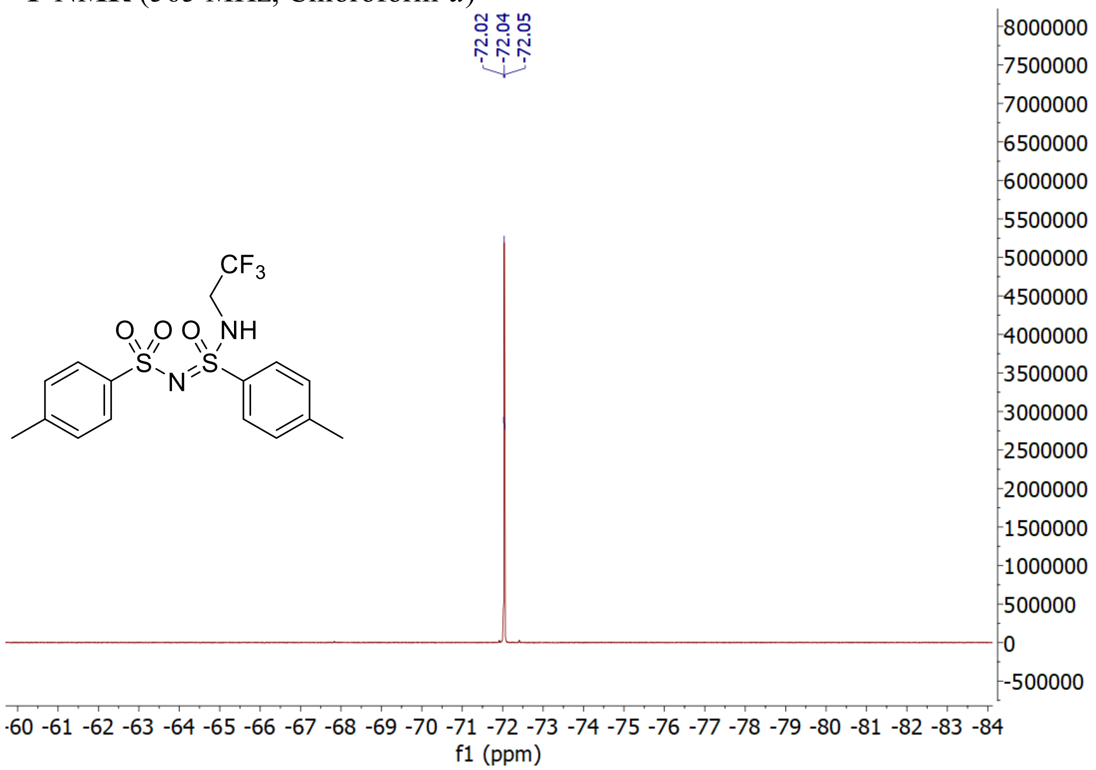

**$N$-(((Cyclopropylmethyl)amino)(oxo)(*p*-tolyl)-$\lambda^6$-sulfaneylidene)-4-methylbenzenesulfonamide (12ac)**

$^1$**H NMR** (600 MHz, Chloroform-*d*)

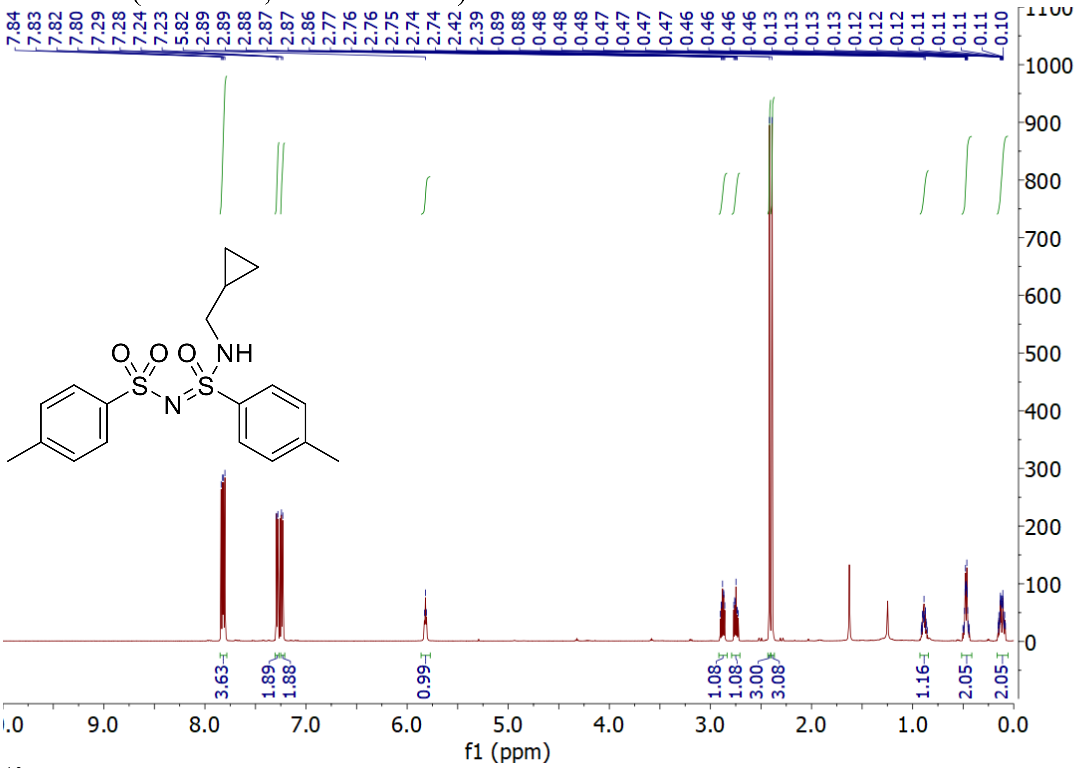


$^{13}$**C NMR** (151 MHz, Chloroform-*d*)

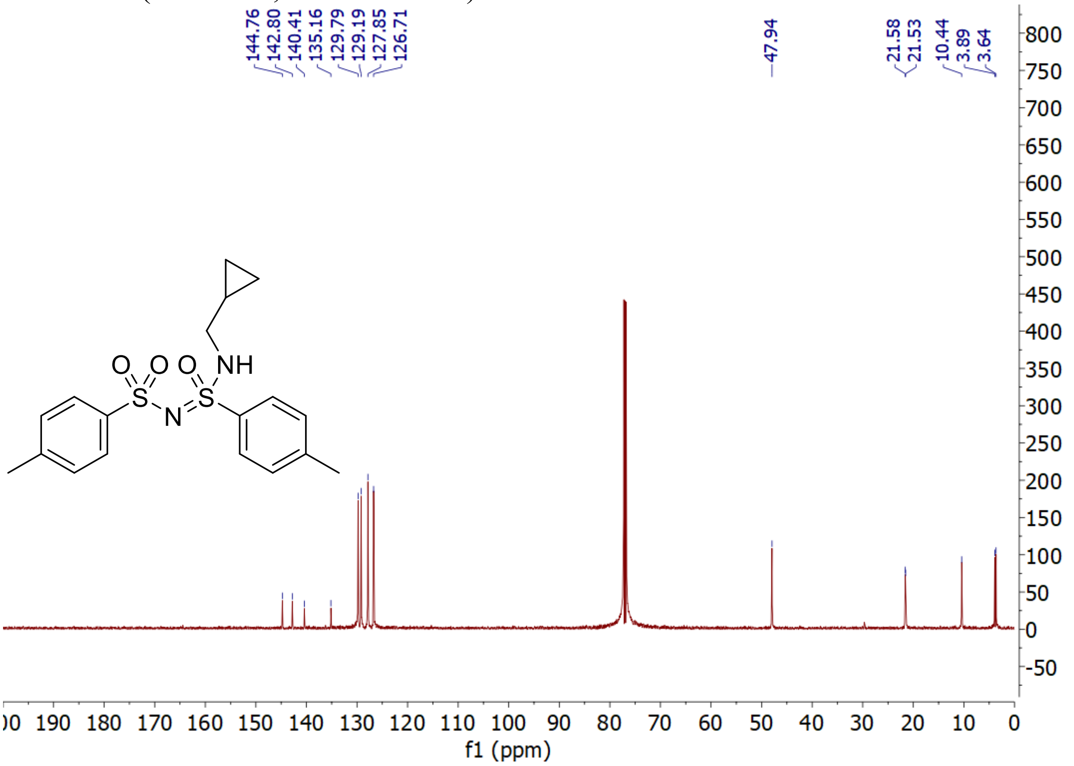

***N*-(((2-Methoxyethyl)amino)(oxo)(*p*-tolyl)-λ⁶-sulfaneylidene)-4-methylbenzenesulfonamide (12ad)**

[1]**H NMR** (600 MHz, Chloroform-*d*)

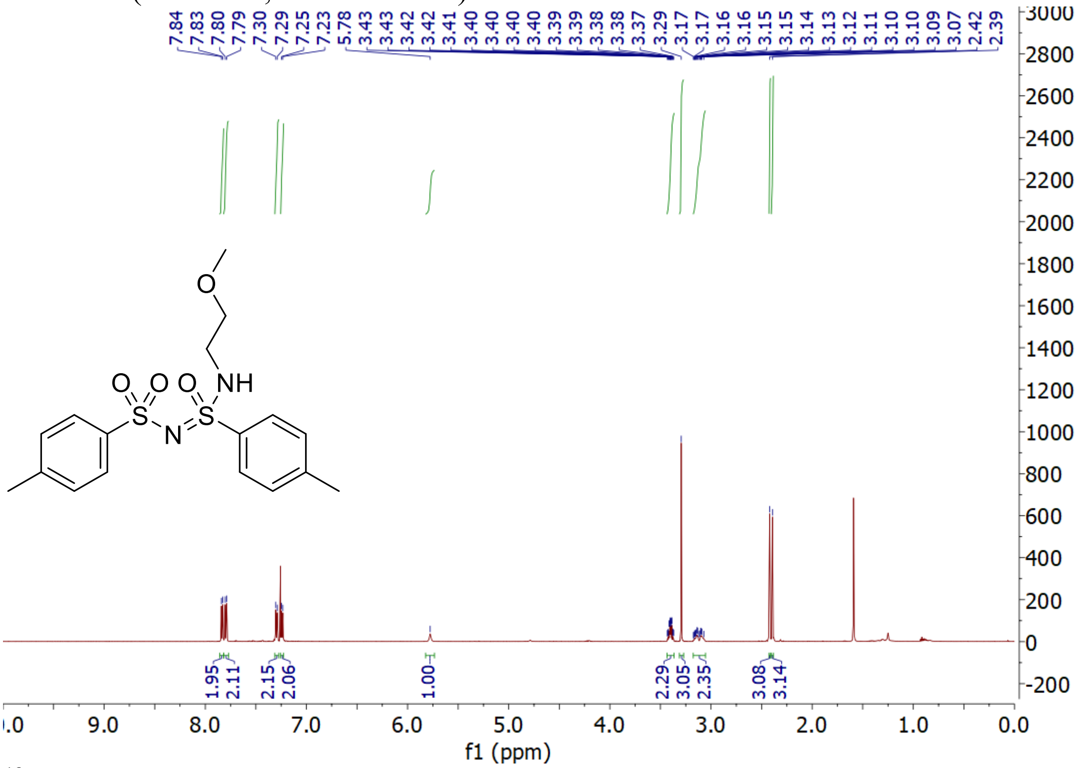

[13]**C NMR** (151 MHz, Chloroform-*d*)

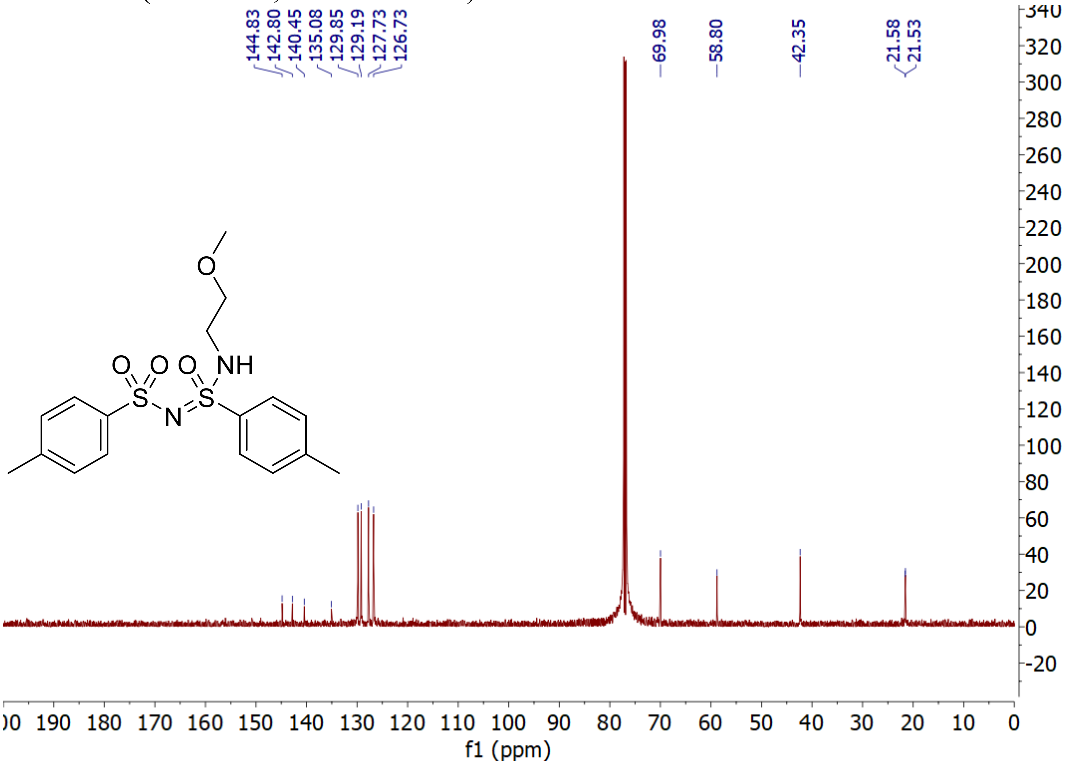

**N-(Amino(4-(5-methyl-3-phenylisoxazol-4-yl)phenyl)(oxo)-λ$^6$-sulfaneylidene)-4-methylbenzenesulfonamide (12ae)**

**$^1$H NMR** (600 MHz, DMSO-$d_6$)

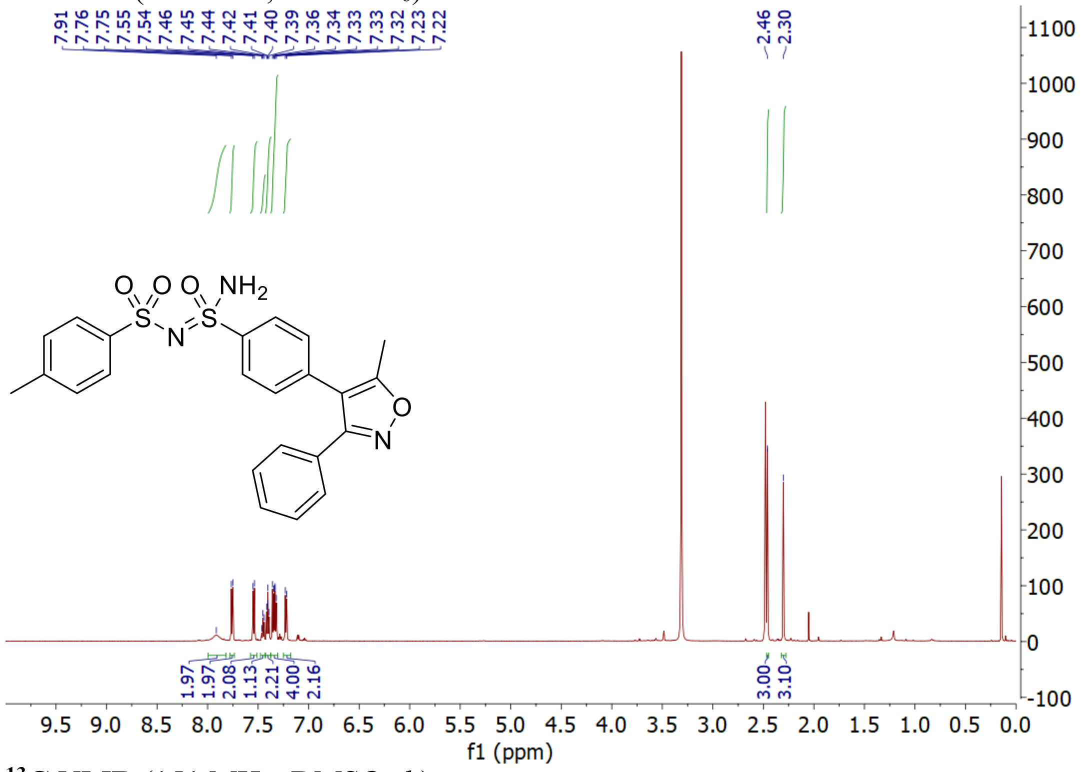

**$^{13}$C NMR** (151 MHz, DMSO-$d_6$)

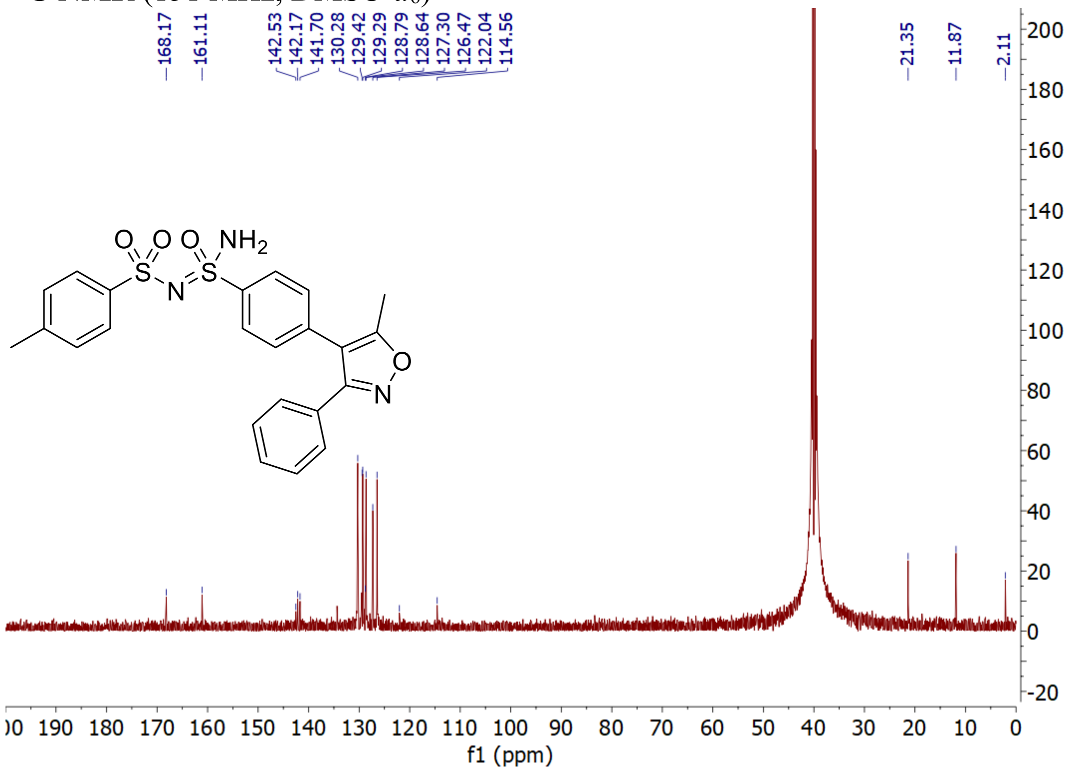

**N-(Amino(oxo)(phenyl)-λ⁶-sulfaneylidene)-4-(5-methyl-3-phenylisoxazol-4-yl)benzenesulfonamide (12af)**

**$^{1}$H NMR** (600 MHz, DMSO-$d_6$)

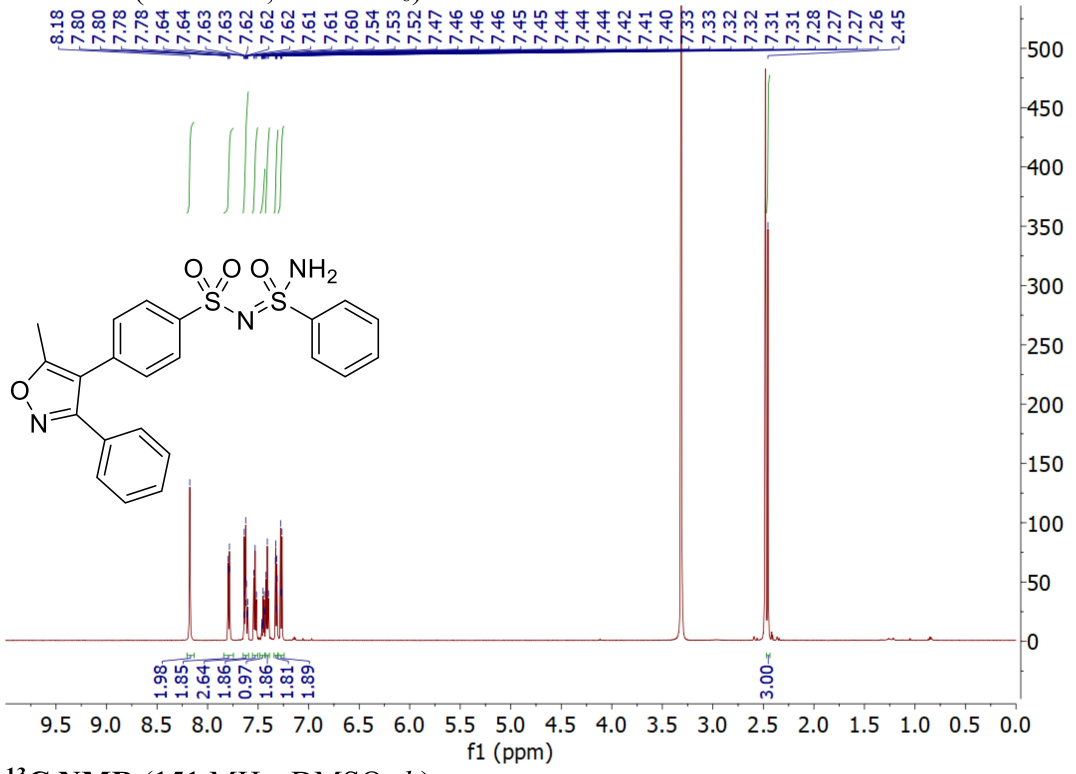


**$^{13}$C NMR** (151 MHz, DMSO-$d_6$)

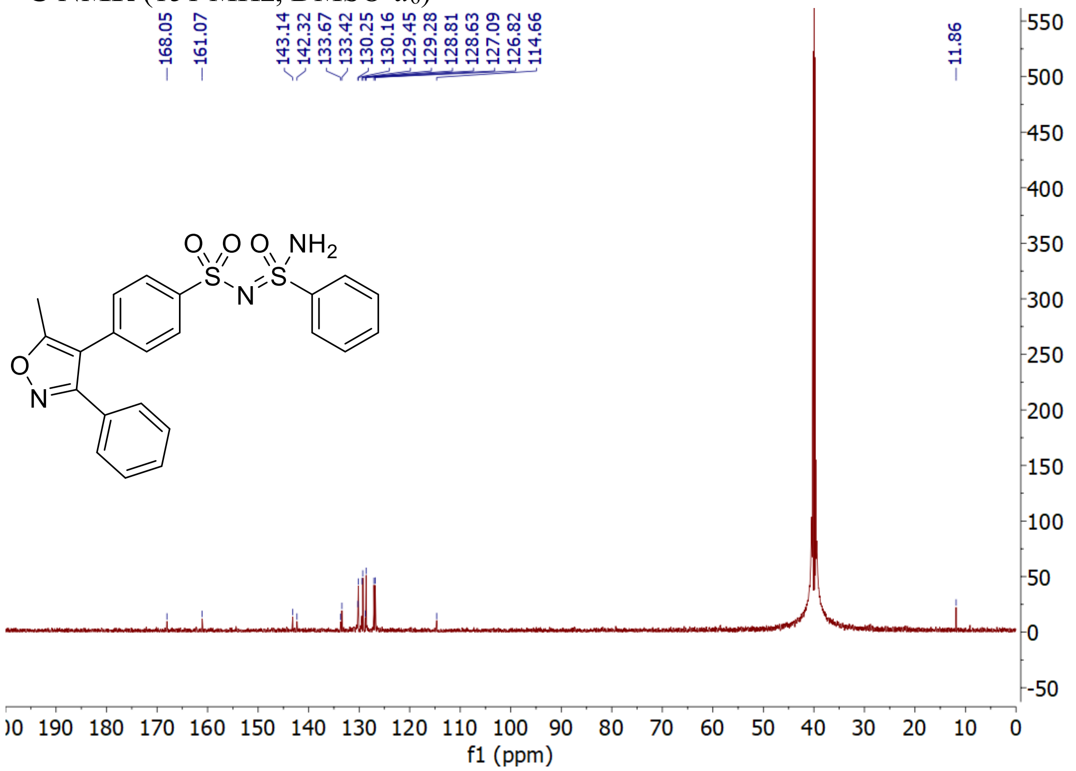

**N-(Amino(oxo)(phenyl)-λ⁶-sulfaneylidene)-4-(5-(*p*-tolyl)-3-(trifluoromethyl)-1*H*-pyrazol-1-yl)benzenesulfonamide (12ag)**

**$^{1}$H NMR** (600 MHz, DMSO-$d_6$)

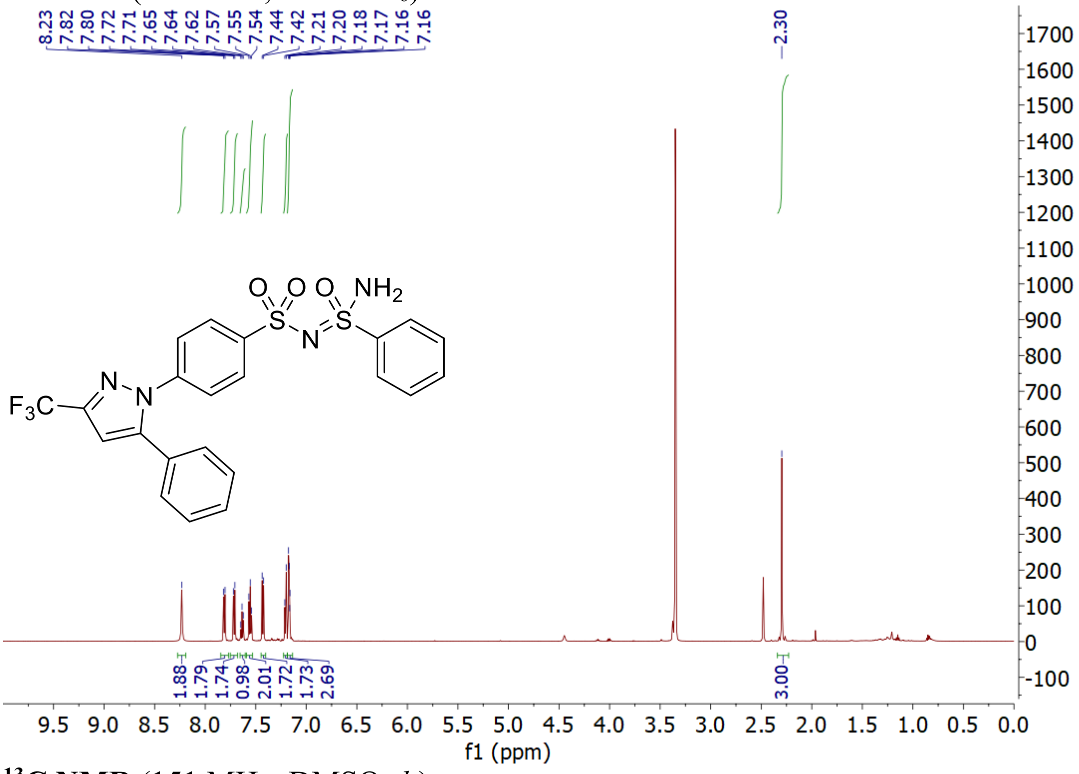


**$^{13}$C NMR** (151 MHz, DMSO-$d_6$)

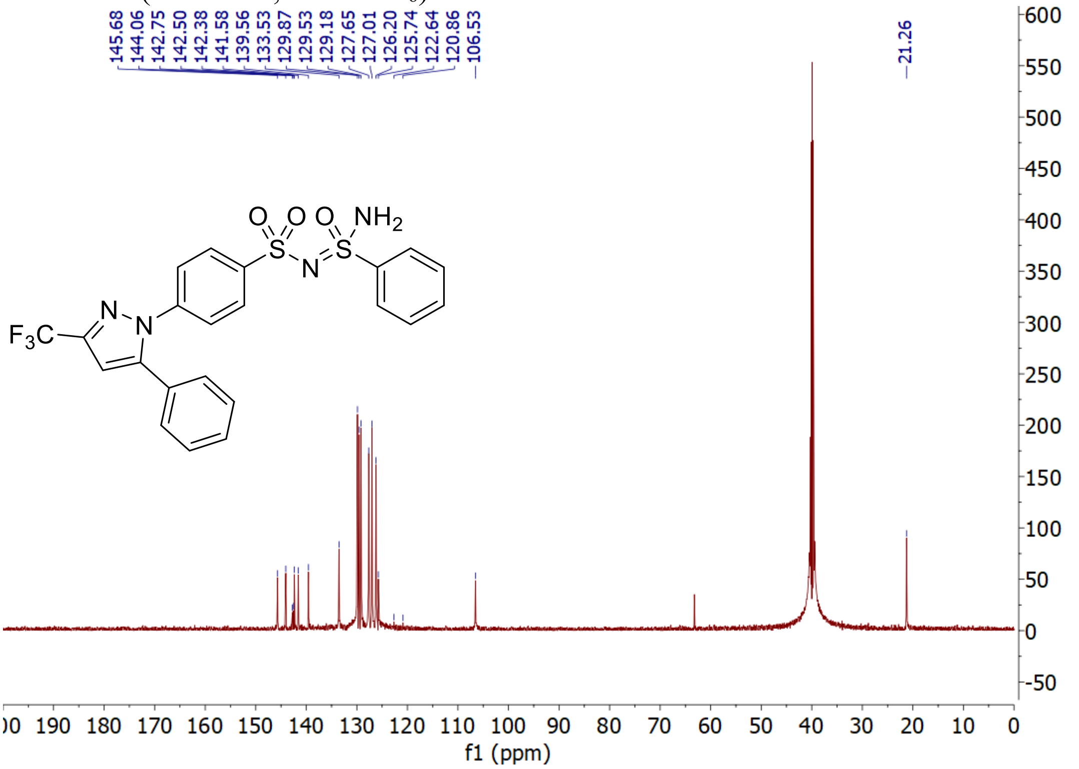

**$^{19}$F NMR** (564 MHz, DMSO-$d_6$)

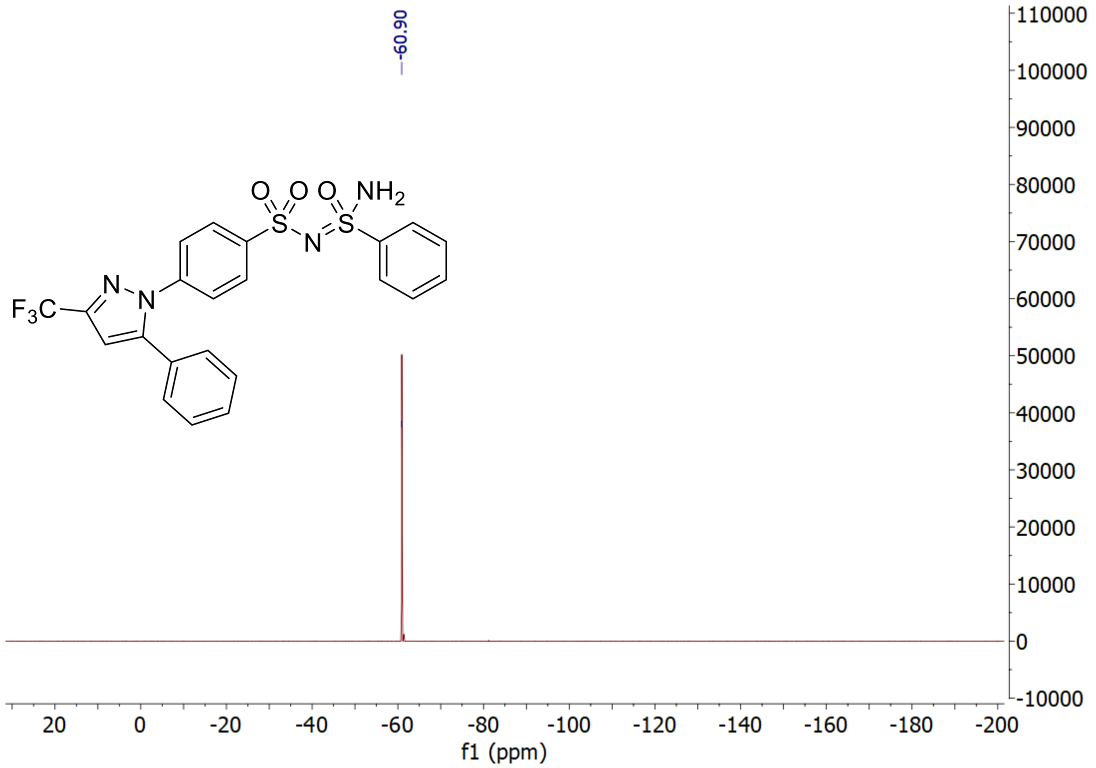

**4-Methyl-*N*-(((methyl(phenyl)-λ⁴-sulfaneylidene)amino)(oxo)(phenyl)-λ⁶-sulfaneylidene)benzenesulfonamide (14)**

**$^{1}$H NMR** (600 MHz, Chloroform-*d*)

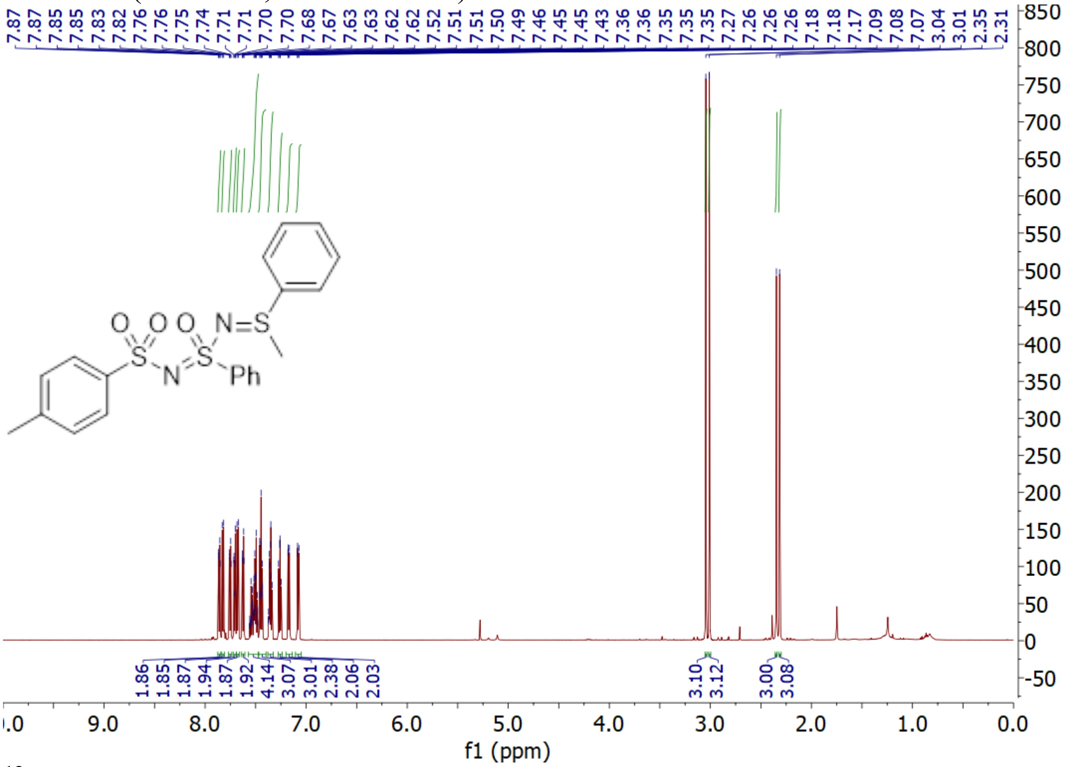


**$^{13}$C NMR** (151 MHz, Chloroform-*d*)

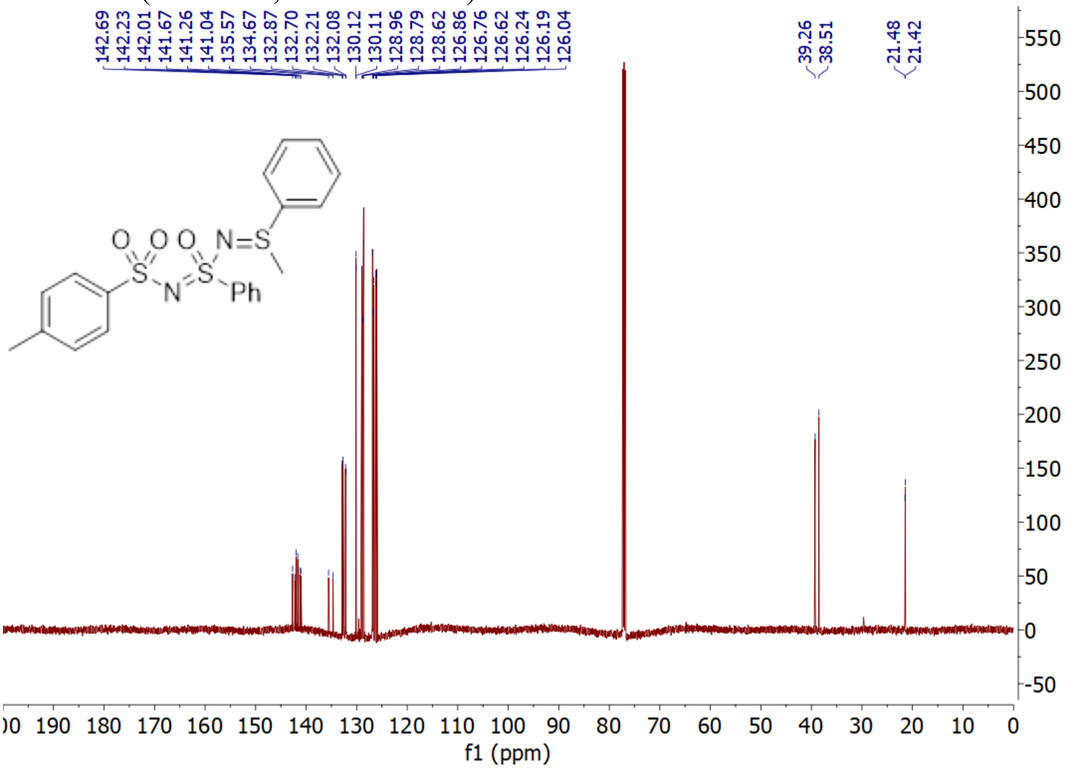

**$N$-(((Dimethyl(oxo)- λ$^{6}$-sulfaneylidene)amino)(oxo)(phenyl)- λ$^{6}$-sulfaneylidene)-4-methylbenzenesulfonamide (16)**

**$^{1}$H NMR** (600 MHz, Chloroform-*d*)

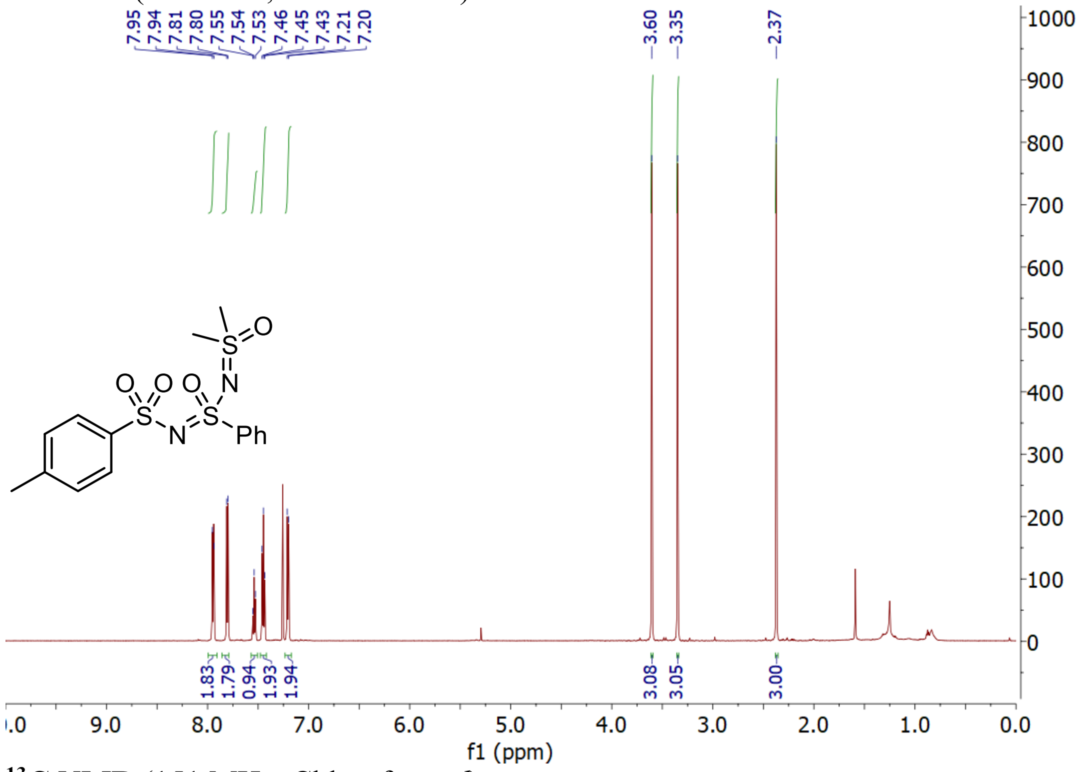

**$^{13}$C NMR** (151 MHz, Chloroform-*d*)

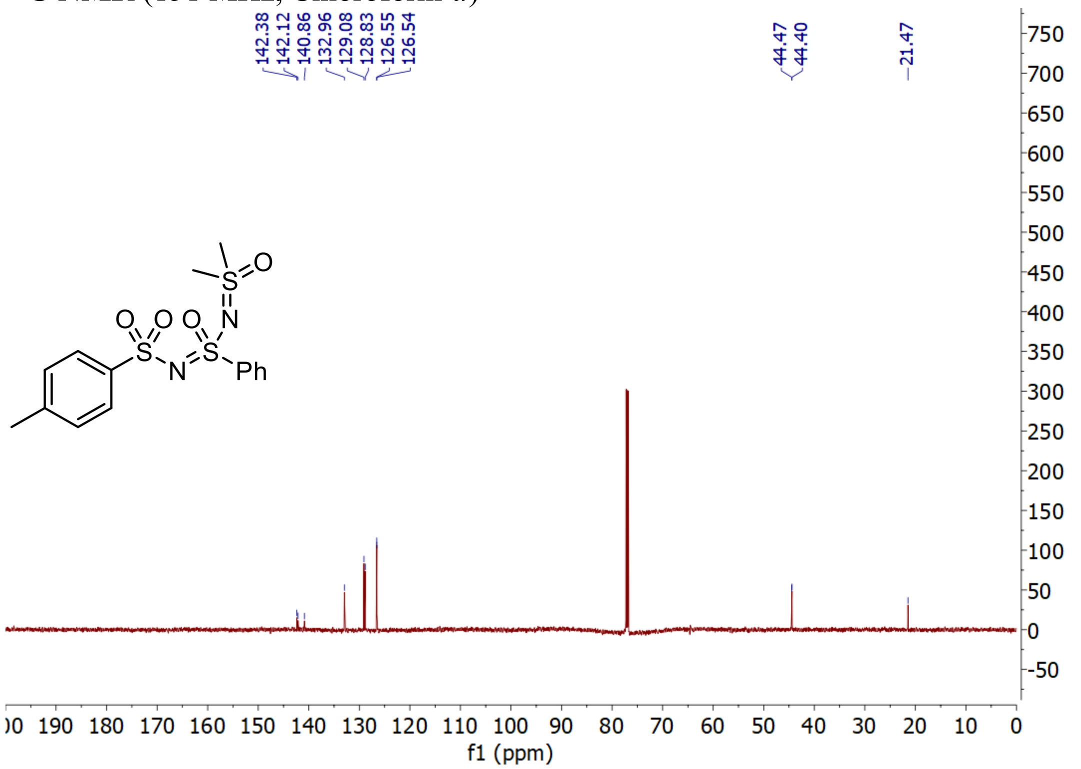

**4*N*-((Allylamino)(oxo)(phenyl)-λ⁶-sulfaneylidene)-4-methylbenzenesulfonamide (18)**

**$^{1}$H NMR** (600 MHz, Chloroform-*d*)

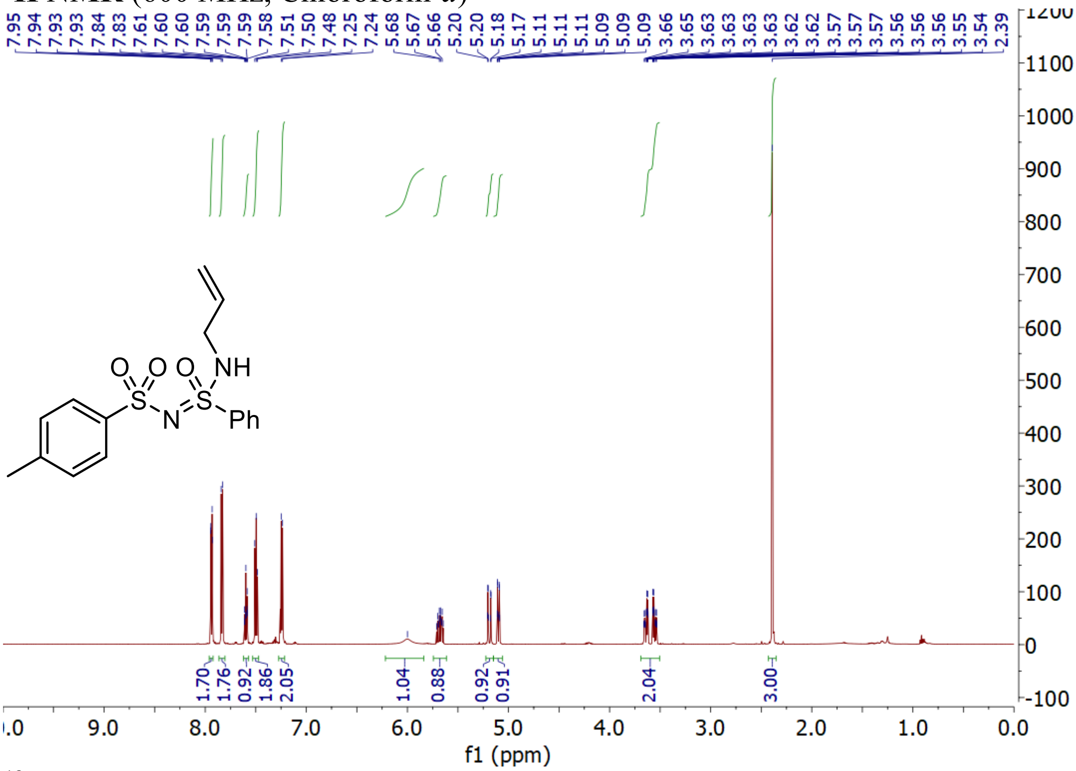


**$^{13}$C NMR** (151 MHz, Chloroform-*d*)

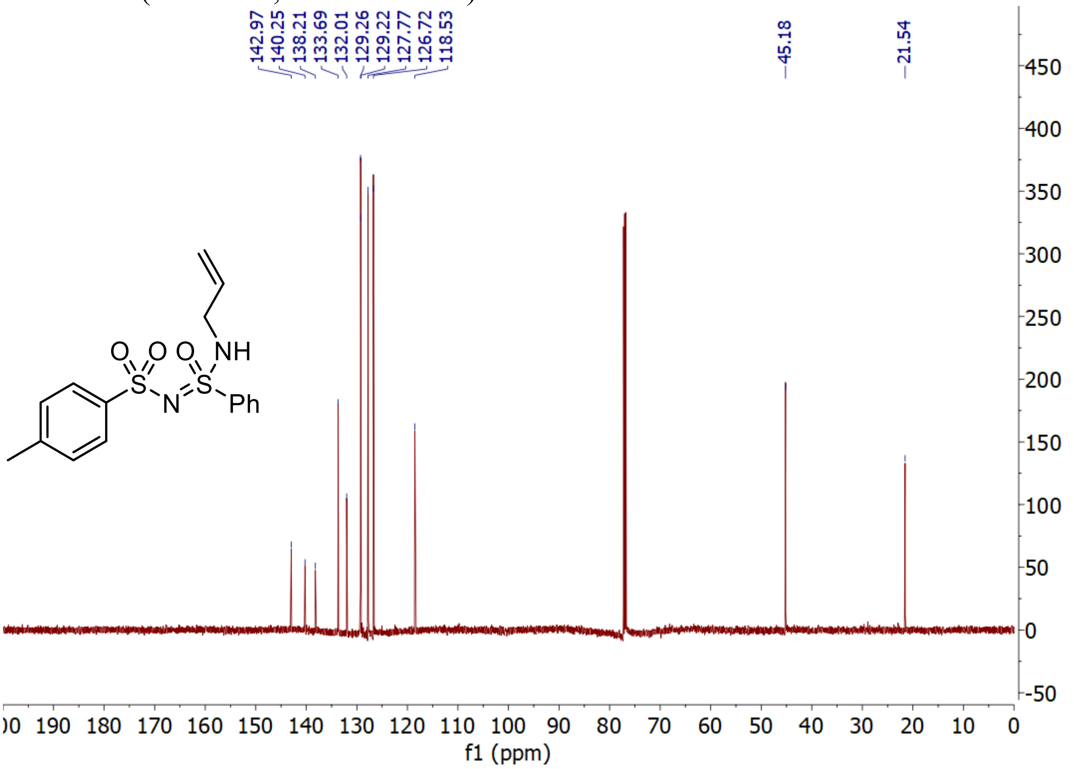

**4-Methyl-*N*-(oxo(phenyl)(2-phenylaziridin-1-yl)-λ$^6$-sulfaneylidene) benzenesulfonamide (19a)**

**$^1$H NMR** (600 MHz, Chloroform-*d*)

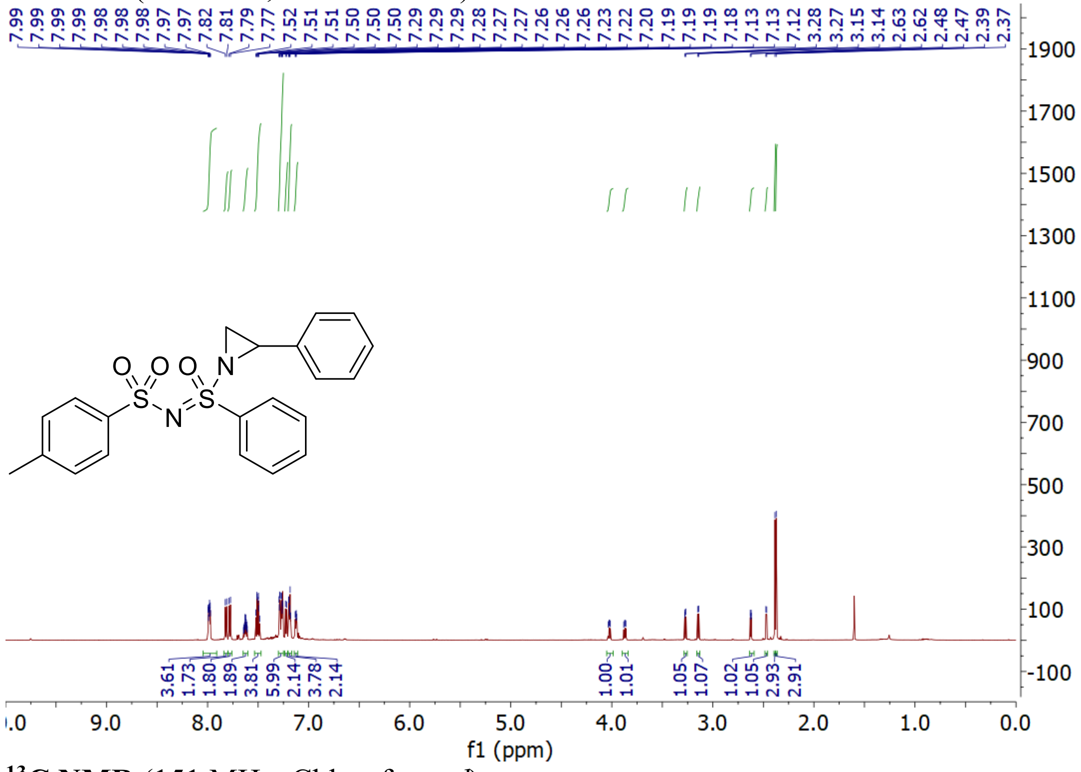


**$^{13}$C NMR** (151 MHz, Chloroform-*d*)

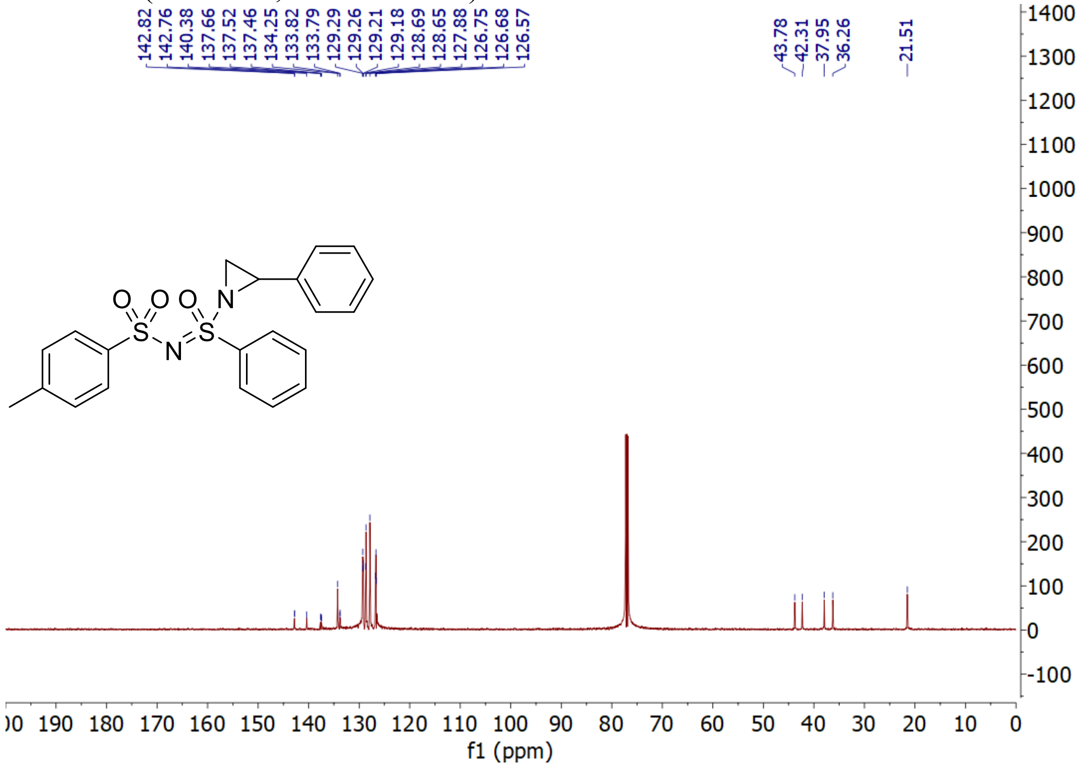

**4-(5-Methyl-3-phenylisoxazol-4-yl)-*N*-(oxo(phenyl)(2-phenylaziridin-1-yl)-λ⁶-sulfaneylidene)benzenesulfonamide (19b)**

**$^{1}$H NMR** (600 MHz, Chloroform-*d*)

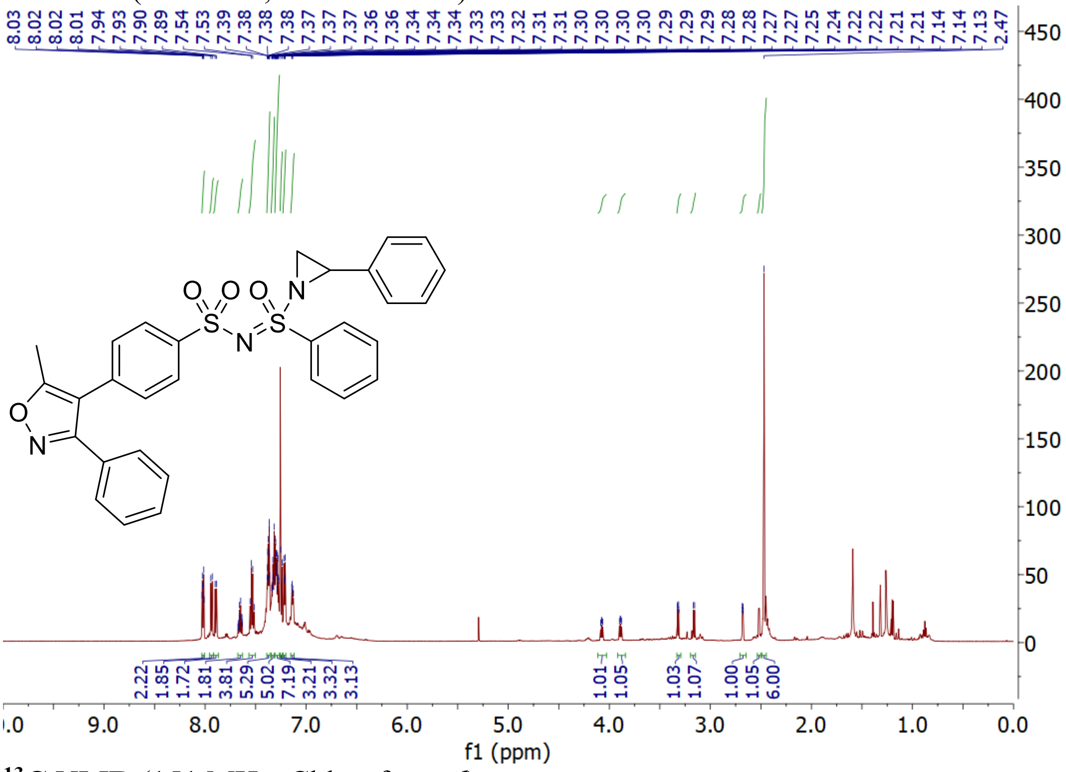


**$^{13}$C NMR** (151 MHz, Chloroform-*d*)

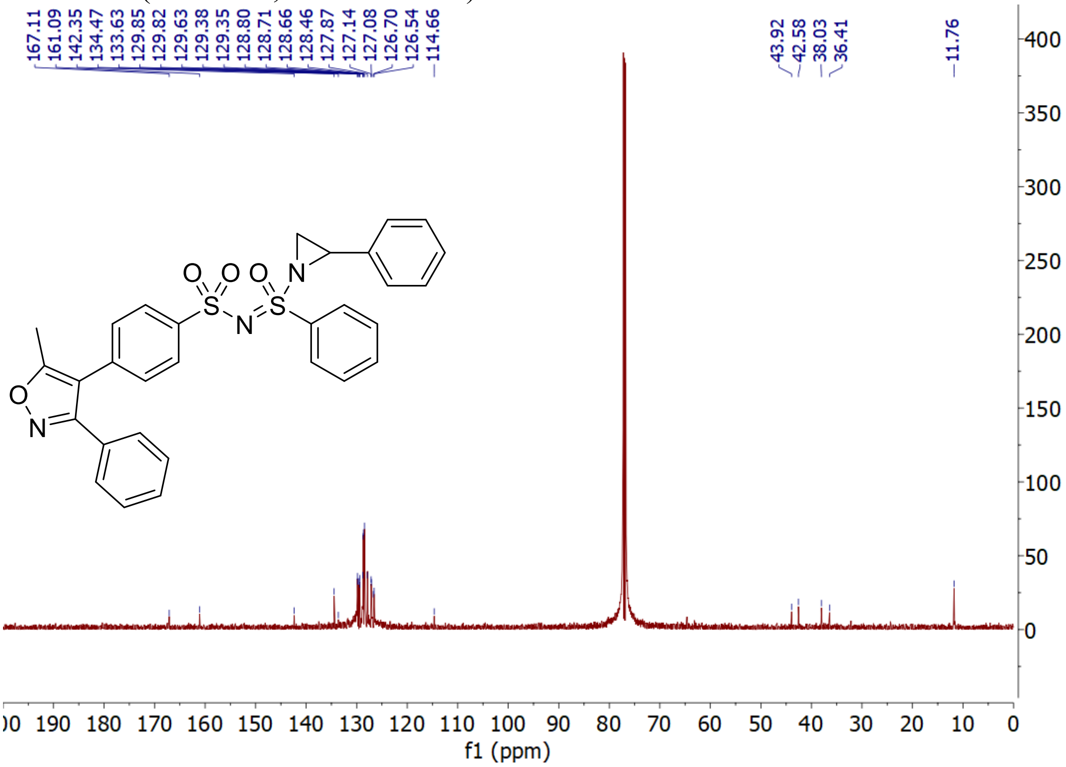

**Diethyl (3a*R*,6a*S*)-2-(*N*-tosylphenylsulfonimidoyl)hexahydrocyclopenta[*c*]pyrrole-5,5(1*H*)-dicarboxylate (20a)**

**$^{1}$H NMR** (600 MHz, Chloroform-*d*)

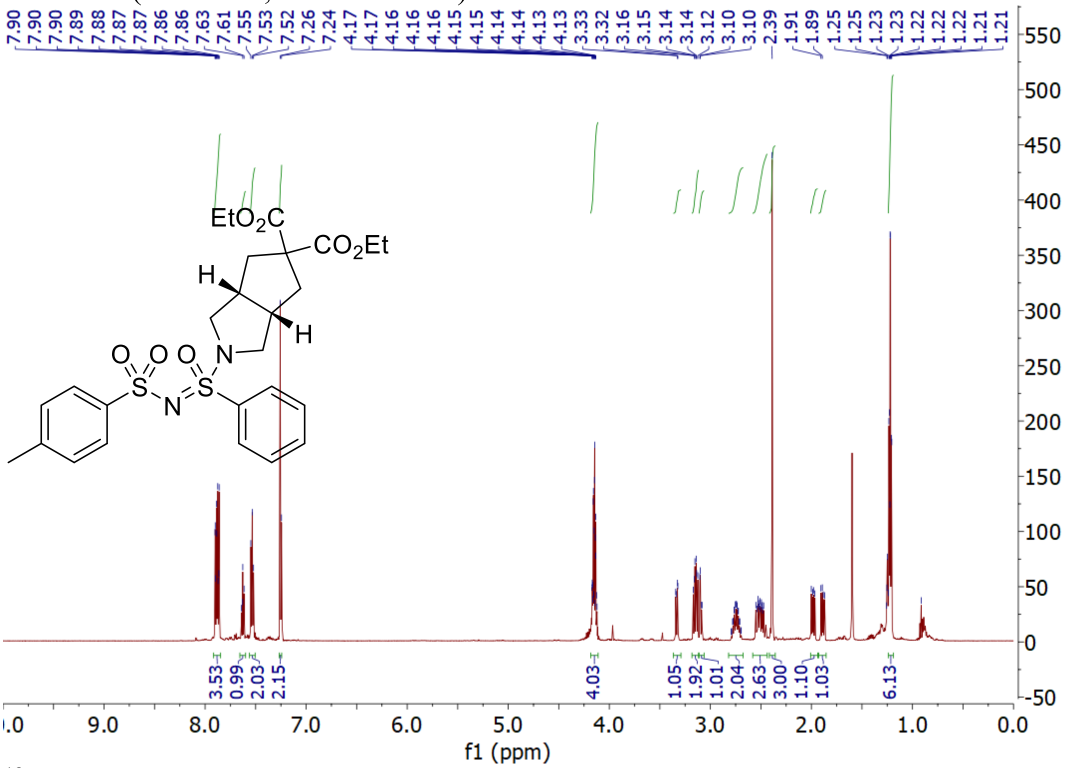


**$^{13}$C NMR** (151 MHz, Chloroform-*d*)

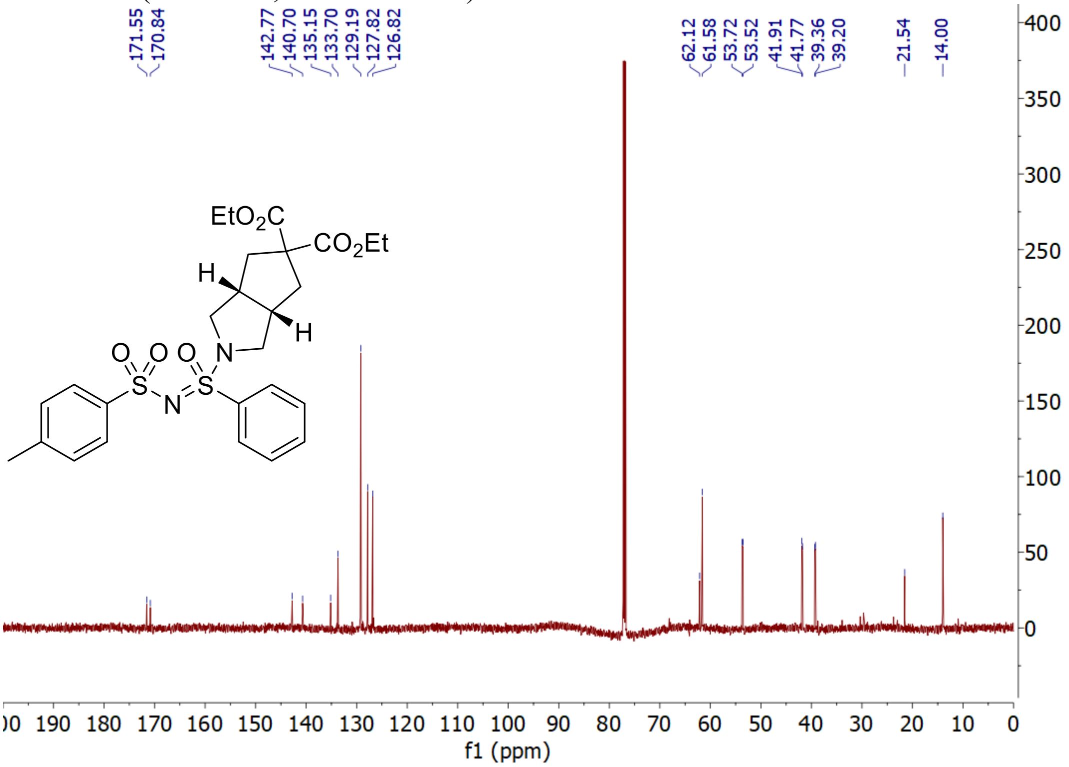

**_N_-(((3a_R_,6a_S_)-Hexahydrocyclopenta[_c_]pyrrol-2(1_H_)-yl)(oxo)(phenyl)-λ⁶-sulfaneylidene)-4-methylbenzenesulfonamide (20b)**

**¹H NMR** (600 MHz, Chloroform-_d_)

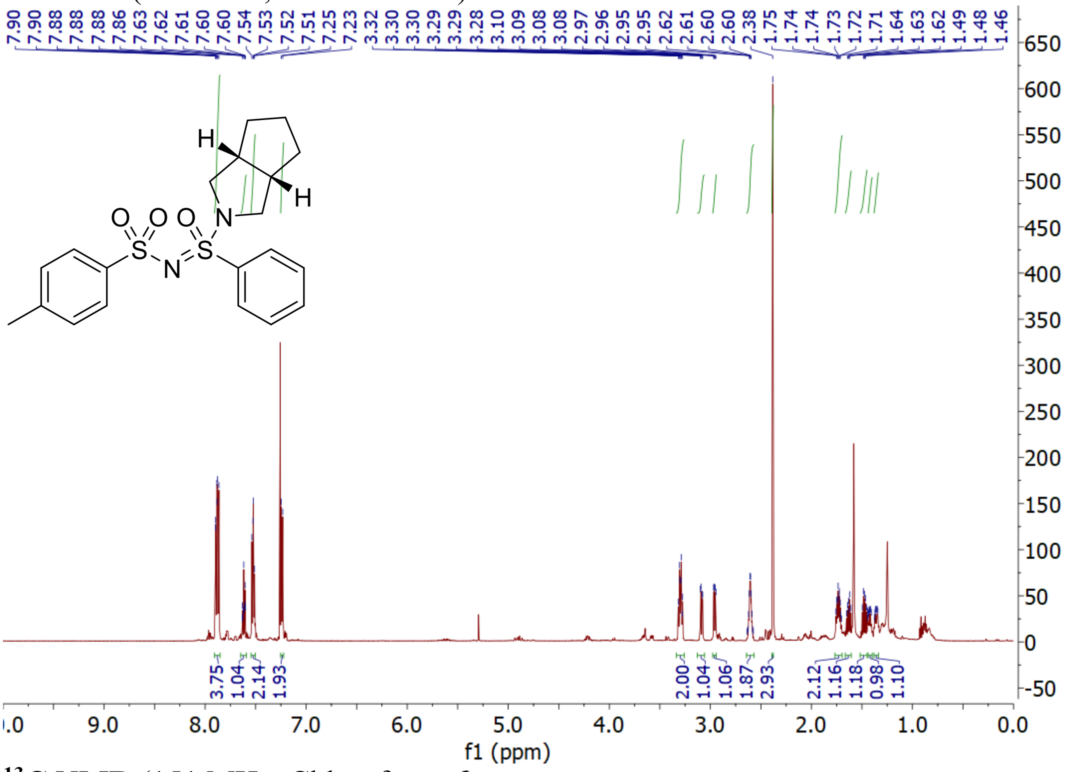


**¹³C NMR** (151 MHz, Chloroform-_d_)

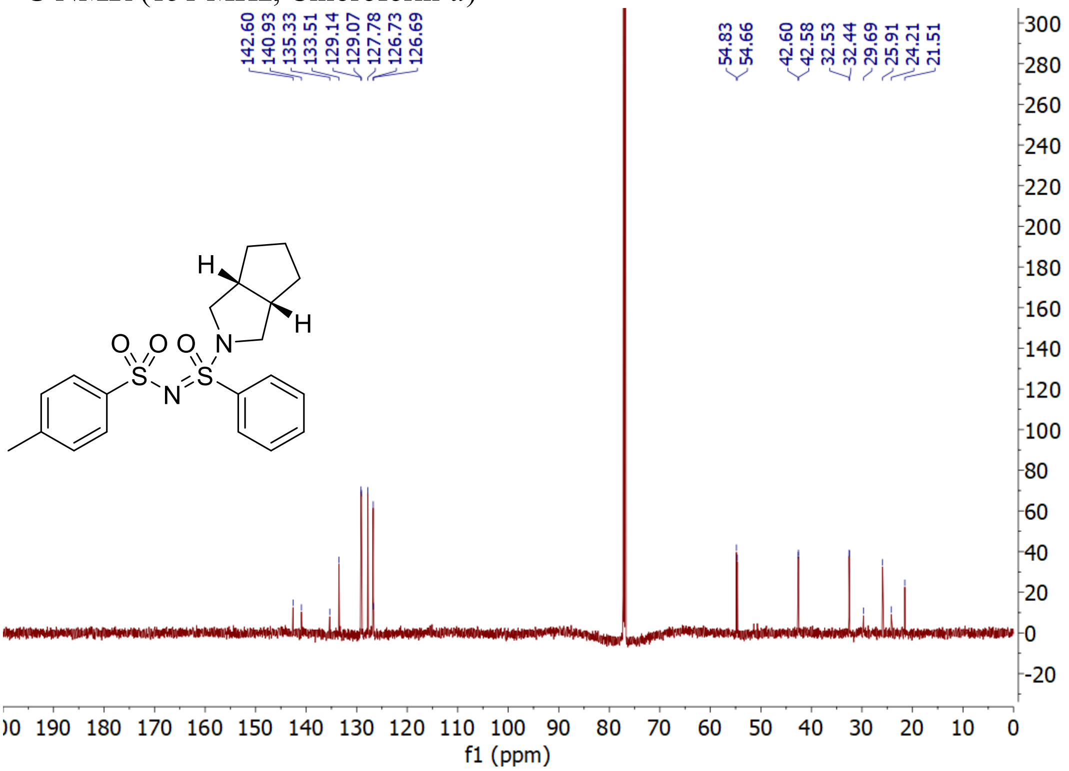

**Diethyl (3a*R*,6a*S*)-2-(*N*-((4-(5-methyl-3-phenylisoxazol-4-yl)phenyl)sulfonyl)phenylsulfonimidoyl)hexahydrocyclopenta[*c*]pyrrole-5,5(1*H*)-dicarboxylate (20c)**

[1]**H NMR** (600 MHz, Chloroform-*d*)

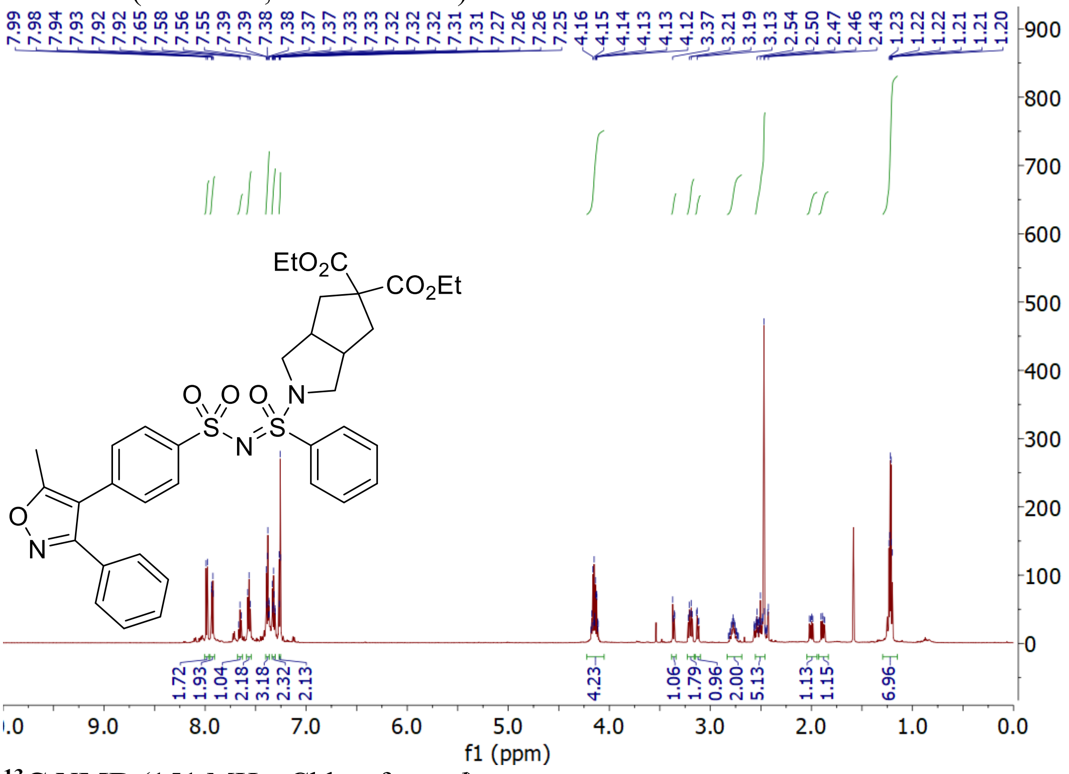


[13]**C NMR** (151 MHz, Chloroform-*d*)

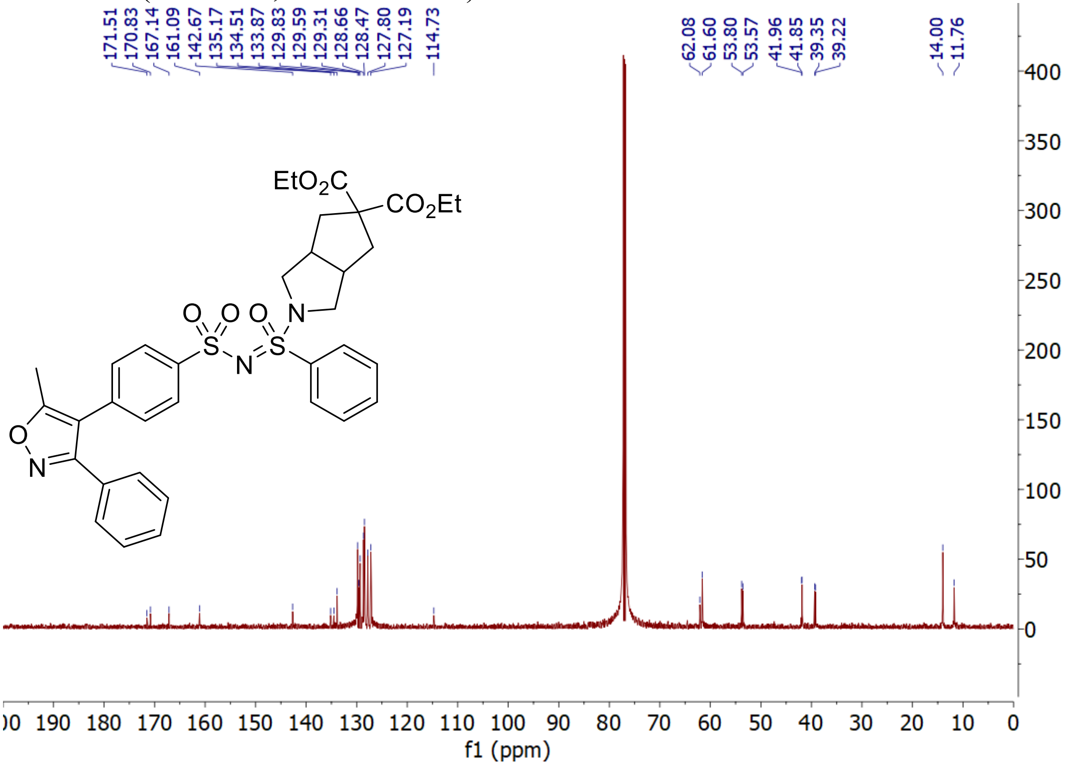

**Diethyl (3a*R*,6a*S*)-2-(4-bromo-*N*-((4-bromophenyl)sulfonyl)phenylsulfonimidoyl) hexahydrocyclopenta[*c*]pyrrole-5,5(1*H*)-dicarboxylate (20d)**

**$^{1}$H NMR** (600 MHz, Chloroform-*d*)

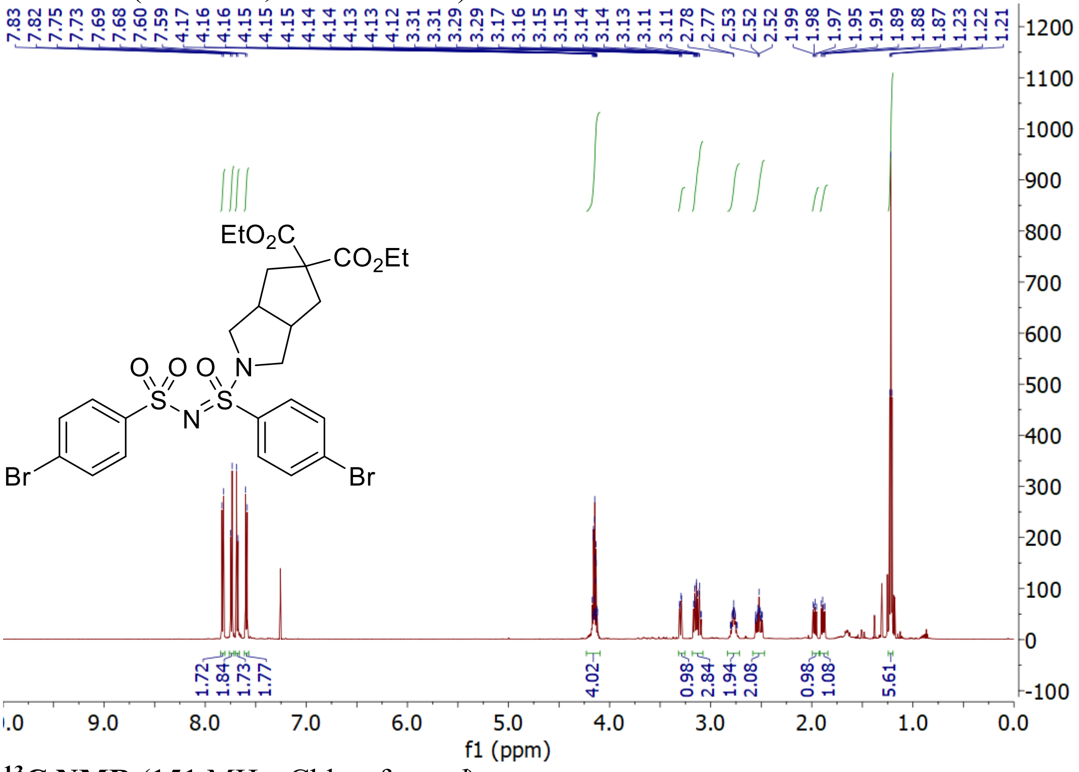


**$^{13}$C NMR** (151 MHz, Chloroform-*d*)

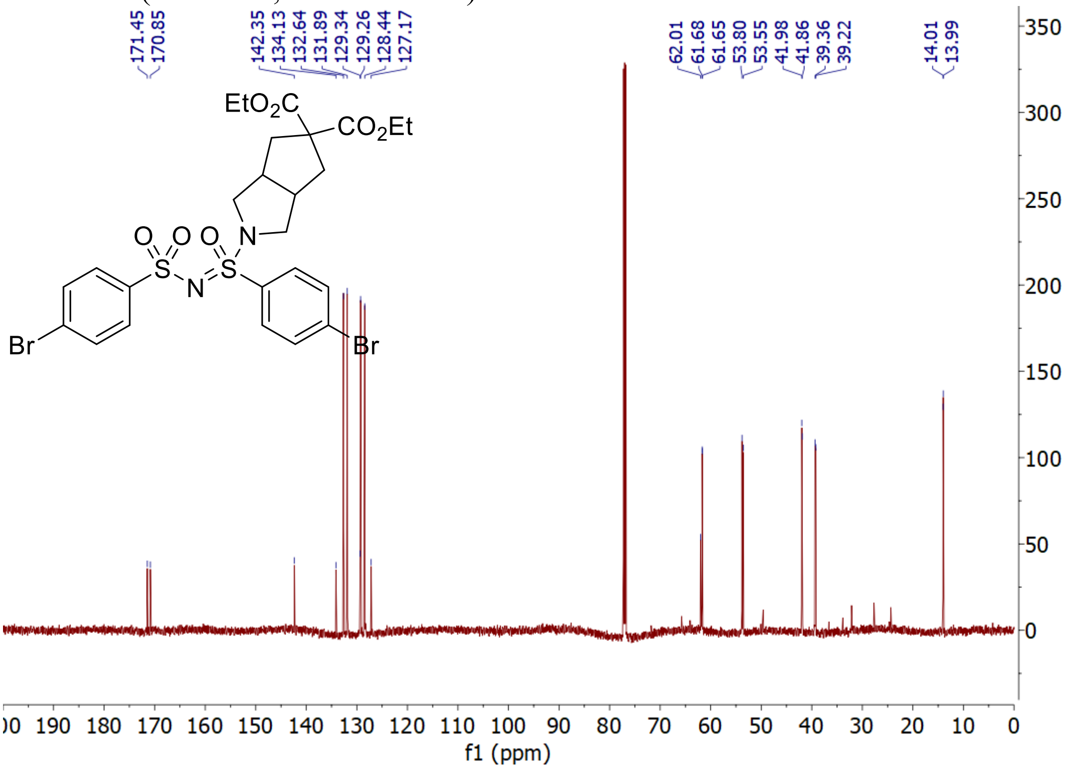

Supplement: Supplementary file 1 — Supporting Information [file ANIE-64-e202509870-s001.pdf]
